# Molecular mechanism of a large conformational change of the quinone cofactor in the semiquinone intermediate of bacterial copper amine oxidase†

**DOI:** 10.1039/d2sc01356h

**Published:** 2022-08-23

**Authors:** Mitsuo Shoji, Takeshi Murakawa, Shota Nakanishi, Mauro Boero, Yasuteru Shigeta, Hideyuki Hayashi, Toshihide Okajima

**Affiliations:** Center for Computational Sciences, University of Tsukuba 1-1-1 Tennodai Tsukuba 305-8577 Ibaraki Japan mshoji@ccs.tsukuba.ac.jp; JST-PRESTO 4-1-8 Honcho Kawaguchi 332-0012 Saitama Japan; Department of Biochemistry, Osaka Medical and Pharmaceutical University 2-7 Daigakumachi Takatsuki 569-8686 Osaka Japan; Institute of Scientific and Industrial Research, Osaka University 8-1 Mihogaoka Ibaraki 567-0047 Osaka Japan; University of Strasbourg, Institut de Physique et Chimie des Matériaux de Strasbourg, CNRS, UMR 7504 23 rue du Loess F-67034 France; Department of Chemistry, Osaka Medical and Pharmaceutical University 2-7 Daigakumachi Takatsuki 569-8686 Osaka Japan

## Abstract

Copper amine oxidase from *Arthrobacter globiformis* (AGAO) catalyses the oxidative deamination of primary amines *via* a large conformational change of a topaquinone (TPQ) cofactor during the semiquinone formation step. This conformational change of TPQ occurs in the presence of strong hydrogen bonds and neighboring bulky amino acids, especially the conserved Asn381, which restricts TPQ conformational changes over the catalytic cycle. Whether such a semiquinone intermediate is catalytically active or inert has been a matter of debate in copper amine oxidases. Here, we show that the reaction rate of the Asn381Ala mutant decreases 160-fold, and the X-ray crystal structures of the mutant reveals a TPQ-flipped conformation in both the oxidized and reduced states, preceding semiquinone formation. Our hybrid quantum mechanics/molecular mechanics (QM/MM) simulations show that the TPQ conformational change is realized through the sequential steps of the TPQ ring-rotation and slide. We determine that the bulky side chain of Asn381 hinders the undesired TPQ ring-rotation in the oxidized form, favoring the TPQ ring-rotation in reduced TPQ by a further stabilization leading to the TPQ semiquinone form. The acquired conformational flexibility of TPQ semiquinone promotes a high reactivity of Cu(i) to O_2_, suggesting that the semiquinone form is catalytically active for the subsequent oxidative half-reaction in AGAO. The ingenious molecular mechanism exerted by TPQ to achieve the “state-specific” reaction sheds new light on a drastic environmental transformation around the catalytic center.

## Abstract 2

The large conformational change of topaquinone in bacterial copper amine oxidase occurs through the TPQ ring rotation and slide, which are essential to stabilize the semiquinone form.

## Graphical abstract

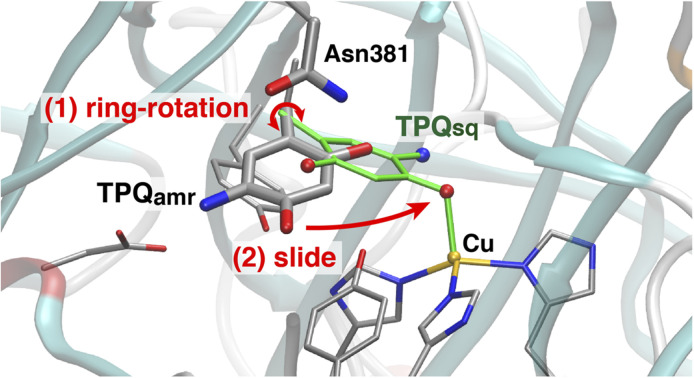

## 1 Introduction

A vast majority of enzyme-catalysed reactions proceed through multiple elementary processes realizing a series of catalytic intermediates.^1,2^ In every single process connecting the intermediates, various catalysed chemical events occur such as nucleophilic, electrophilic, and elimination reactions, and electron/proton transfer. These processes tend to induce conformational changes in the active-site residue(s)/cofactor, which in turn have the effect of enhancing the reactivity. Generally, conformational diversity and multiple states are key factors enhancing the catalytic functions;^3^ on the other hand, limiting the conformational changes is also important to achieve high catalytic activity.^4^ These enzymatic molecular mechanisms are further complicated by unclarified protonation and electronic states. All these uncertainties make enzymatic reactions very difficult to reveal. The catalytic intermediates can be detected spectrophotometrically, or by X-ray crystallography as freeze-trapped structures giving insightful information to unveil their reaction mechanisms, provided that they are transiently accumulated depending on the rate constants of the reaction steps. Nonetheless, both spectroscopic and structural characterization studies of transient conformational changes and unaccumulated intermediates remain elusive. To overcome this difficulty, computational approaches making use of hybrid quantum mechanics/molecular mechanics (QM/MM) and molecular dynamics (MD) simulations are nowadays a reliable tool to provide information not accessible to experimental probes. These well-assessed techniques allow for an unambiguous determination of reaction pathways, including conformational changes, and disclose the atomic-level structural details of the short-lived transition state of each elemental process.

Copper amine oxidases (CAOs) catalyse the oxidative deamination of primary amines into their corresponding aldehydes and exert fundamental functions in a wealth of aerobic organisms from bacteria to yeast, plants, and mammals.^5–7^ CAOs in microorganisms have a nutritional role in catabolizing primary amines.^7^ In higher eukaryotes, CAOs in animals participate in the degradation of bio-active amines, and regulation of cell adhesion, cell death, and collagen cross-linking.^7^ CAOs in plants exert an active role in wound healing, cell growth, and biosynthesis of various compounds including some alkaloids and lignin.^7^ CAOs have a homodimer structure with a subunit molecular mass of 70–95 kDa.^8–10^ The active site is buried inside a large β-sandwich domain, containing one divalent copper ion (Cu(ii)) and a redox-active organic cofactor, topaquinone (TPQ)^11,12^ originating from the post-translational modification of a specific tyrosine residue *via* a copper and oxygen-dependent autocatalytic reaction.^13^

**Fig. 1:**
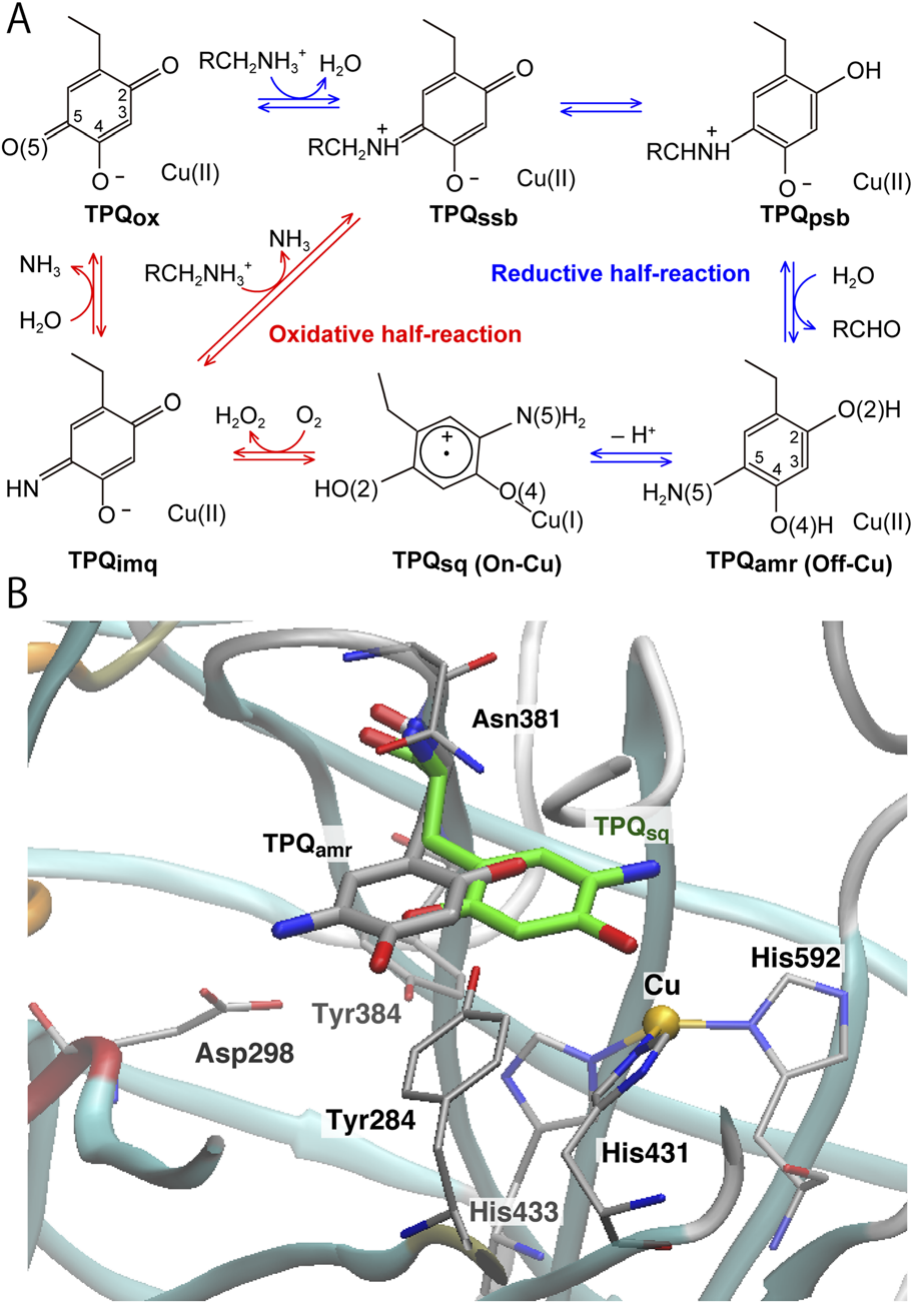
(A) The proposed catalytic cycle of AGAO.^14^ The stable and intermediate states are the oxidized form (TPQ_ox_), the substrate Schiff base (TPQ_ssb_), the product Schiff base (TPQ_psb_), aminoresorcinol (TPQ_amr_), the semiquinone radical (TPQ_sq_), and iminoquinone (TPQ_imq_). Among all these, only TPQ_sq_ is detected in the on-copper conformation. (B) The conformational changes of TPQ in the aminoresorcinol and semiquinone radical states. TPQ_amr_ is located away from the Cu coordination site (off-copper), while TPQ_sq_, colored in green, approaches the Cu site (on-copper). Two X-ray crystal structures of AGAO are superimposed (PDB ID: 3X3X, 3X3Z).^14^

The catalytic reaction of CAO is composed of two half-reactions, one reductive and the other oxidative, as shown in Fig. 1A.^14,15^ During the reductive half-reaction, an initial oxidized form of TPQ (TPQ_ox_) is converted into the substrate Schiff base (TPQ_ssb_) through the nucleophilic attack of a substrate amine on the O5 carbonyl group. TPQ_ssb_ is further converted into the product Schiff base (TPQ_psb_) through stereospecific proton transfer. Then, TPQ_psb_ is hydrolyzed to the corresponding aldehyde and aminoresorcinol (TPQ_amr_). The latter is equilibrated with a semiquinone radical (TPQ_sq_) plus monovalent Cu(i) that is formed by a single electron transfer from TPQ_amr_ to Cu(ii).

Using CAO of the soil bacterium *Arthrobacter globiformis* (AGAO), we have performed transient kinetic experiments for the reductive half-reaction to spectrophotometrically detect the TPQ_ssb_, TPQ_psb_, TPQ_amr_, and TPQ_sq_ intermediates.^16,17^ Furthermore, we have determined the X-ray crystal structures of all these intermediate states trapped in AGAO crystals. In the initial TPQ_ox_ state, the TPQ ring is located away from Cu(ii) (“off-copper” conformation) with the O4 of the TPQ ring forming a hydrogen bond (H-bond) with a highly conserved Tyr residue (Tyr284). This off-copper conformation is preserved in TPQ_ssb_, TPQ_psb_, and TPQ_amr_. Interestingly, a large conformational change of the TPQ ring is observed in the reaction step going from TPQ_amr_ to TPQ_sq_;^14,15^ through this process, the O4 of the TPQ ring is ligated axially to the Cu in TPQ_sq_ (“on-copper” conformation). For the conformational change of TPQ_amr_, the TPQ ring needs to undertake three motions: sliding (rotation of 53° around the Cα–Cβ bond), tilting up (20° rigid body rotation centered on the Cα atom), and phenol ring rotation (rotation of 180° around the Cβ–Cγ bond).^14^ This structurally challenging mechanism is further complicated by the occurrence of a one-electron transfer from TPQ_amr_ to Cu(ii) and a concomitant deprotonation of the O4 of TPQ_amr_, which are all required for TPQ_sq_ formation. Since the TPQ ring in the off-copper conformation is surrounded by a number of amino acid residues including Asp298, Asn381, and Tyr384, it has so far been expected that the bulky TPQ ring does not have enough free space to undergo a rotation, and that the TPQ ring should first slide out from the off-copper position, and then revolve into the on-copper position.^14^ However, this pathway has never been detailed. Among the residues in proximity of the off-copper TPQ ring, Asn381 is highly conserved in CAOs, and the side chain carboxamide group is situated on the TPQ ring of TPQ_ox_.^18,19^ The spectroscopic study presented in ref. 18 for *Hansenula polymorpha* CAO (HPAO) suggests that the Asn residue, analogous to Asn381 in AGAO, prevents TPQ_ox_ from taking a nonproductive orientation by suppressing the mobility of the cofactor. The Asn381 residue is located close to the TPQ ring in both off-copper and on-copper conformations. As a result, the side chain is likely to affect the TPQ conformational change from TPQ_amr_ to TPQ_sq_ (Fig. 1B). When the other active-site residues are mutated by site-directed mutagenesis, various effects concerning the conformational changes or thermal flexibility were reported in former studies.^17,20,21^ However, the specific contribution of Asn381 to this process is still unclear, as, to date, there are only static structural data on the active-site structure of AGAO.

Several former studies focused on the flexibilities of the TPQ cofactor in TPQ_ox_, and on-copper, off-copper active and off-copper flipped conformations were confirmed.^8,9,22–24^ These conformational flexibilities of TPQ_ox_ represent an important feature in the early stage of the process after TPQ biogenesis, where TPQ in CAO is produced from a Tyr residue on the Cu site and TPQ has to change the conformation to the catalytic site. Conversely, for the catalytic intermediates, 3D structures in non-off-copper and on-copper conformations are very limited in TPQ_sq_.^14,25^ To preserve the optimal catalytic activity of TPQ, accurate control of the TPQ conformation is essential, and a conformational change along with an alternation of TPQ contributes directly to the inactivation.^18,23^ We remark that TPQ_sq_ formation depends on the CAO types and the source organisms, and the formation of TPQ_sq_ is not always observed for all CAOs.^14,25^ AGAO, *Pisum sativum* CAO (PSAO) and *Escherichia coli* CAO (ECAO) undergo the TPQ conformational change during TPQ_sq_ formation, whereas bovine serum CAO (BSAO) and HPAO do not undergo any TPQ conformational change and TPQ_sq_ is not formed during the catalytic cycle.^14,25^ For AGAO, PSAO and ECAO, spectroscopy studies have shown that TPQ_sq_ is generated in equilibrium with TPQ_amr_,^26,27^ and their TPQ_sq_ are essential intermediates in the catalytic cycle. The formation of TPQ_sq_ influences the reoxidization step by O_2_ to generate an iminoquinone intermediate (TPQ_imq_) and hydrogen peroxide.^14,27,28^ The reaction steps in the oxidative-half reaction still remain an open issue, and may also vary depending on TPQ_sq_ formation. Give this scenario, it is crucial to address the questions of why and how TPQ_sq_ formation and the large conformational change of TPQ occur in the AGAO catalytic cycle.

In the present study, we investigated the conformational changes in the quinone cofactor in AGAO using a synergy of experimental and theoretical methods. The conformational changes of the Asn381Ala (N381A) mutant were determined by X-ray crystallography and kinetic analyses. Then QM/MM methods were employed to unravel the conformational change pathways and electron transfer mechanism in the TPQ_amr_ to TPQ_sq_ transition. The contributions of the active-site residues close to TPQ and Cu(ii) along the TPQ conformation are also inspected within the same computational approach. The insight provided by this work evidences the active role of Asn381 in the exceptionally large conformational change in TPQ_amr_ and elucidates the electron transfer mechanism from TPQ_amr_ to Cu(ii) which is strictly dependent on the conformation and deprotonation of the TPQ ring. The large conformational change in the quinone cofactor observed in AGAO is a clear and detailed example of the conformational control exerted by the active-site residues in promoting and enhancing multistep and multi-conformational enzymatic reactions.

## 2 Materials and methods

### 2.1 Preparation, characterization and X-ray crystallography of the N381A mutant of AGAO

Details for site-directed mutagenesis (N381A), enzyme preparation, and kinetics analyses for TPQ biogenesis and enzyme catalysis are reported in the ESI.† A holo form of the N381A mutant was crystallized by a microdialysis method with the crystallization buffer, 1.05 M potassium-sodium tartrate in 25 mM HEPES buffer, pH 6.8, as described in a former work for the wild-type (WT) enzyme.^17^ For the determination of the crystal structures of the catalytic intermediates anaerobically reduced using 2-phenylethylamine (2-PEA), the holo-form crystals in the dialysis button were transferred into the new reservoir solution containing 45% (v/v) glycerol in an anaerobic glove box (SGV-65V glove box, AS ONE corporation) at 16 °C for 24 h. Then, the crystals were soaked in a new reservoir solution containing 45% (v/v) glycerol and 4 mM 2-PEA at 16 °C for 60 min and subsequently frozen by rapid cooling in liquid CF_4_. Before exposure to X-rays, these crystals were analysed by single-crystal microspectrophotometry at 100 K as previously reported.^17^ The X-ray diffraction data were collected at 100 K with synchrotron X-radiation (*λ* = 0.9 Å) using an MX-225HE detector (Rayonix, L.L.C.) in the BL44XU beamline station at the SPring-8 facility (Hyogo, Japan). The collected data were processed and scaled using Mosflm^29^ and Scala in CCP4,^30^ respectively. Molecular replacement for phase determination was done by Phaser^31^ using the WT AGAO structure (PDB ID: 1IU7)^13^ as a search model. The obtained initial structure was subjected to rigid-body refinement and was further refined with Phenix.^32^ The manual model building and its validation were performed with Coot.^33^ Ramachandran plots were calculated using MolProbity^34^ for structure validation. The details and statistics of crystallographic refinement are summarized in Table S2.† Atomic coordinates and structure factors of the holo form and the substrate-reduced form of N381A AGAO were deposited in the Protein Data Bank with the accession codes 7WIR and 7WIS, respectively.

### 2.2 Computational details

The X-ray crystal structure of AGAO, determined with a resolution of 1.51 Å, was obtained from the Protein Data Bank (PDB ID: 3X3Z).^14^ This crystal structure corresponds to the TPQ_amr_ state in the off-copper conformation. An inhibitor Cl^−^ anion coordinated to the Cu(ii) in the original PDB was removed. Among all the available conformers, residues with the highest occupancy were selected and titratable residues were protonated according to their state in the corresponding neutron crystal structure (PDB ID: 6L9C).^35^ A dimer model was solvated into a water droplet with a 60 Å radius and the whole system was kept in a neutral charge state by replacing some of the water molecules with 36 Na^+^ ions. The whole system was equilibrated *via* classical MD within the Amberff99 force field framework.^36^ This equilibration process was achieved by an annealing MD at 250 K lasting for 10 ps to relax the solvent water molecules and all the added H atoms in AGAO. The coordinates of the heavy atoms determined by X-ray crystallography were kept fixed during this MD stage.

**Fig. 2:**
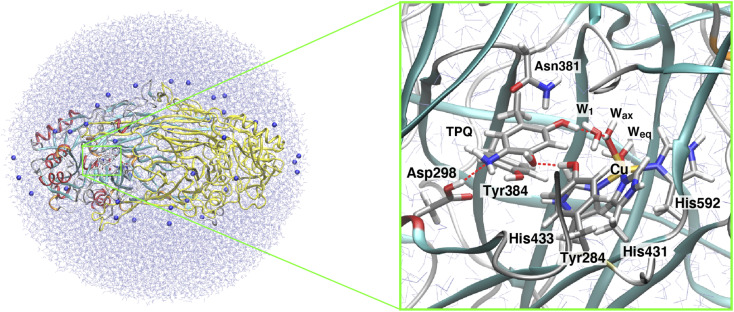
The QM/MM model used in our simulations for the AGAO dimer. In the figure, the AGAO monomers, the Na^+^ ions, and the solvent water molecules are colored differently. The highlighted panel shows the details of the active site. Atoms in the QM region are shown in licorice representation with the main residues and water molecules labeled according to the discussion in the main text.

After this annealing step, we moved to QM/MM simulations. The selected QM subsystem consists of side chains including the residues Tyr284, Asp298, Tyr384, Asn381, TPQ, His431, His433, and His592, plus the Cu(ii) and water molecules in the active site (Fig. 2). For the electronic structure description of the QM region, we resorted to the density functional theory (DFT) in a spin-unrestricted scheme at the UB3LYP-D3/DZVP level. The remaining classical part of the system was treated at the same MM level, with the Amberff99 force field, as used in the equilibration step. The hybrid exchange-correlation functional B3LYP was complemented by Grimme's D3 dispersion correction.^37^ The basis set adopted to describe the electronic structure consists of valence double zeta plus polarized (DZVP) functions, specifically, LANL-2DZ for Cu and 6-31G* for the other atoms.^38–40^ This computational set-up has already been assessed in terms of the accuracy of the structures and energetics of the relevant enzymatic reactions.^41–44^ Geometry optimizations were performed for all the atoms within a 15 Å radius from the centre in the QM region. An electronic embedding scheme and link hydrogen atoms were adapted for the cut across covalent bonds at the QM/MM interface, and QM/MM non-bonded interactions were explicitly computed without introducing a cutoff distance for all the energy calculations. The sampling of the reaction pathways and the location of the transition states was done with the nudged elastic band (NEB) method.^45–47^ We used 13 images for the first rough searches, and in the high energy regions close to the barriers where transition states are expected to be located, we further refined the sampling by performing NEB calculations with an additional 13 images.

The N381A mutant model was constructed by replacing the Asn381 residue with Ala. TPQ_ox_ models for the WT AGAO and the N381A mutant were obtained by replacing the cofactor moieties in the reduced state (TPQ_amr_) with those in the oxidized form (TPQ_ox_).

All the MD and QM/MM calculations were performed using the NWChem 6.8 program package.^48^ The molecular structures shown in the figures were drawn using the VMD program.^49^

## 3 Results and discussion

### 3.1 Characteristics of the N381A mutant

Asn381 is highly conserved among CAOs and is located at the closest position to the TPQ ring. For this reason, it plays a key role in the AGAO catalytic cycle. To evaluate the contribution of Asn381 to the TPQ conformational change and catalytic reactions, we replaced the Asn381 residue with Ala and conducted a kinetic and structural analysis. The WT and the N381A mutant of AGAO were expressed in *E. coli* and purified to homogeneity (Fig. S1†). The fundamental properties of the N381A mutant enzyme are summarized in Table 1 together with those of the WT for comparison. As with TPQ in the WT, TPQ was generated from the precursor Tyr residue in N381A. However, the rate of TPQ biogenesis was reduced to 1/180-fold that in the WT (Fig. S2†). A steady-state kinetic analysis of the overall catalytic reaction with 2-PEA showed a *K*_m_ value for the N381A (1.9-fold) rather similar to that of the WT, but a very low *k*_cat_ value (1/160-fold) for N381A compared with that of the WT enzyme (Table 1). To identify the reaction steps accounting for the significant decrease in the catalytic activity of the N381A mutant, changes in the UV-vis absorption spectra initiated by the addition of 2-PEA were measured under single-turnover (anaerobic) conditions with a stopped-flow spectrometer (Fig. S3†). During the reductive half-reaction of the WT enzyme with 2-PEA, the absorption peak at 480 nm of TPQ_ox_ disappeared in about 4 ms. Then, the two peaks at 316 nm and 410 nm, assigned to TPQ_ssb_ and TPQ_psb_, respectively, arose. Accompanied by a concomitant decay of these two peaks, new peaks assigned to TPQ_sq_ (peaks at 365, 438, and 465 nm) eventually appeared during about 200 ms as shown in Fig. S3B†.^17^ Conversely, for the N381A mutant, the TPQ-derived 495 nm absorption band gradually decreased within 100 ms upon anaerobic mixing with 2-PEA, and no new absorption peak above 300 nm was observed during the reaction (Fig. S3A†). The final spectrum detected in the measurement showed no absorption in the visible region, indicating that TPQ_amr_ is the final product and that the equilibrium between TPQ_amr_ and TPQ_sq_ significantly shifts toward the former. Thus, the absence of the detection of any intermediate state indicates that the initial TPQ_ssb_ formation from TPQ_ox_ is in the rate-limiting step in the reductive half-reaction of the N381A mutant enzyme with the substrate amine.

**Table 1:** Characteristics of the WT and the N381A mutant of AGAO^a^

	Rate of TPQ biogenesis (min^−1^)	Steady-state kinetic parameters	
		*K*_m_ (μM)	*k*_cat_ (s^−1^)
WT	2.300 × 10^−1^ ± 3.4 × 10^−3^ (1)	4.80 ± 0.43 (1)	98.2 ± 5.4 (1)
N381A	1.3000 × 10^−3^ ± 5.7 × 10^−6^ (1/180)	8.90 ± 0.35 (1.9)	0.610 ± 0.012 (1/160)

a Values in parentheses indicate the ratio with the WT AGAO.

### 3.2 X-ray crystallographic analysis of the N381A mutant

**Fig. 3:**
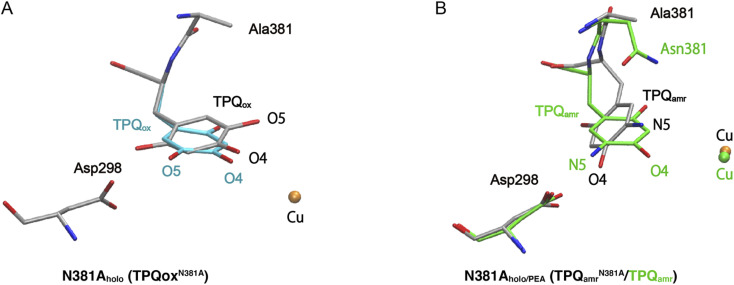
X-ray structures of the holo N381A crystal in the TPQ_ox_ and TPQ_amr_ conformations. The TPQ rings are flipped in both states. (A) The TPQ ring-flipped conformation of TPQ^N381A^_ox_ is colored by element, in which the non-ring-flipped conformation is colored in cyan. (B) The N381A structure of TPQ_amr_ is superimposed on the WT structure (PDBID: 3X3Z),^14^ colored in green for a direct comparison.

The X-ray crystallographic structures were determined for the TPQ_ox_ and TPQ_amr_ forms in the N381A mutant, hereafter indicated as N381A_holo_ and N381A_holo/PEA_, respectively (Table S1†). In the single-crystal UV-vis absorption spectra, N381A_holo_ was characterized by a peak centered at 480 nm corresponding to TPQ_ox_, and N381A_holo/PEA_ exhibited a spectrum similar to that of TPQ_amr_ formed during the reaction with 2-PEA (Fig. S4†), confirming that the TPQ_amr_ intermediate has been successfully freeze-trapped after 2-PEA soaking. As a noticeable feature, the electron density of the active-site structure of N381A_holo_ shows that TPQ can take two conformations as depicted in Fig. 3A. The occupancies of the two conformers, *a* and *b*, of TPQ are 0.47 and 0.53, respectively (Fig. S5†). The conformer *a* of TPQ has essentially the same conformation as the WT TPQ (normal form), whereas the conformer *b* is rotated by about 170° around the Cβ–Cγ axis (flipped form). For both conformers, the C4 hydroxyl group of TPQ forms H-bonds with the highly conserved Tyr residue (Tyr284 in AGAO) with a distance of 2.7 Å and 2.5 Å for the normal and flipped forms, respectively. The conformer *b* has the O5 carbonyl of TPQ not directed toward the substrate-binding site, indicating an inactive conformation.

Concerning the structure of N381A_holo/PEA_, based on single-crystal micro-spectrophotometry and the electron density maps, we modelled TPQ_amr_ for the cofactor at the position of the amino acid residue 382. In the structure, the electron density corresponding to the product phenylacetaldehyde was observed at the active site. A remarkable feature is a fact that TPQ_amr_ presents a flipped form with rotations of about 20° around the Cα–Cβ bond and about 150° around the Cβ–Cγ bond with respect to the off-copper conformation of the WT structure^14^ (Fig. 3B). Details of the active site and TPQ_amr_ in the N381A mutant are shown in Fig. S6.† The O4 atom of the phenol of TPQ_amr_ in the N381A mutant approached Asp298, or rather moved away from Cu. This TPQ_amr_ still does not reach an on-copper conformation. This is consistent with the evidence that the final conformer identified by the spectral change of the reductive half-reaction of the N381A mutant enzyme with 2-PEA is assigned to TPQ_amr_ but not to TPQ_sq_. It is evident that the N381A mutant enzyme lacks the ability for the cofactor to retain an appropriate conformation in both TPQ_ox_ and the substrate-reduced forms (TPQ_amr_/TPQ_sq_) for the catalytic reaction. In particular, the alternative position of TPQ_amr_ in the N381A mutant suggests that specific and selective TPQ conformational regulations are required for the WT-AGAO catalytic reactions, especially for TPQ_sq_ formation. Hence, by resorting to detailed QM/MM simulations, presented in the following paragraphs, we aim to elucidate the conformational change pathway from TPQ_amr_ to TPQ_sq_ associated with electron transfer to the Cu centre, focusing on the N381A mutant lacking the WT catalytic ability because of its peculiar conformation.

### 3.3 Relation between TPQ protonation and TPQ_sq_ formation

**Fig. 4:**
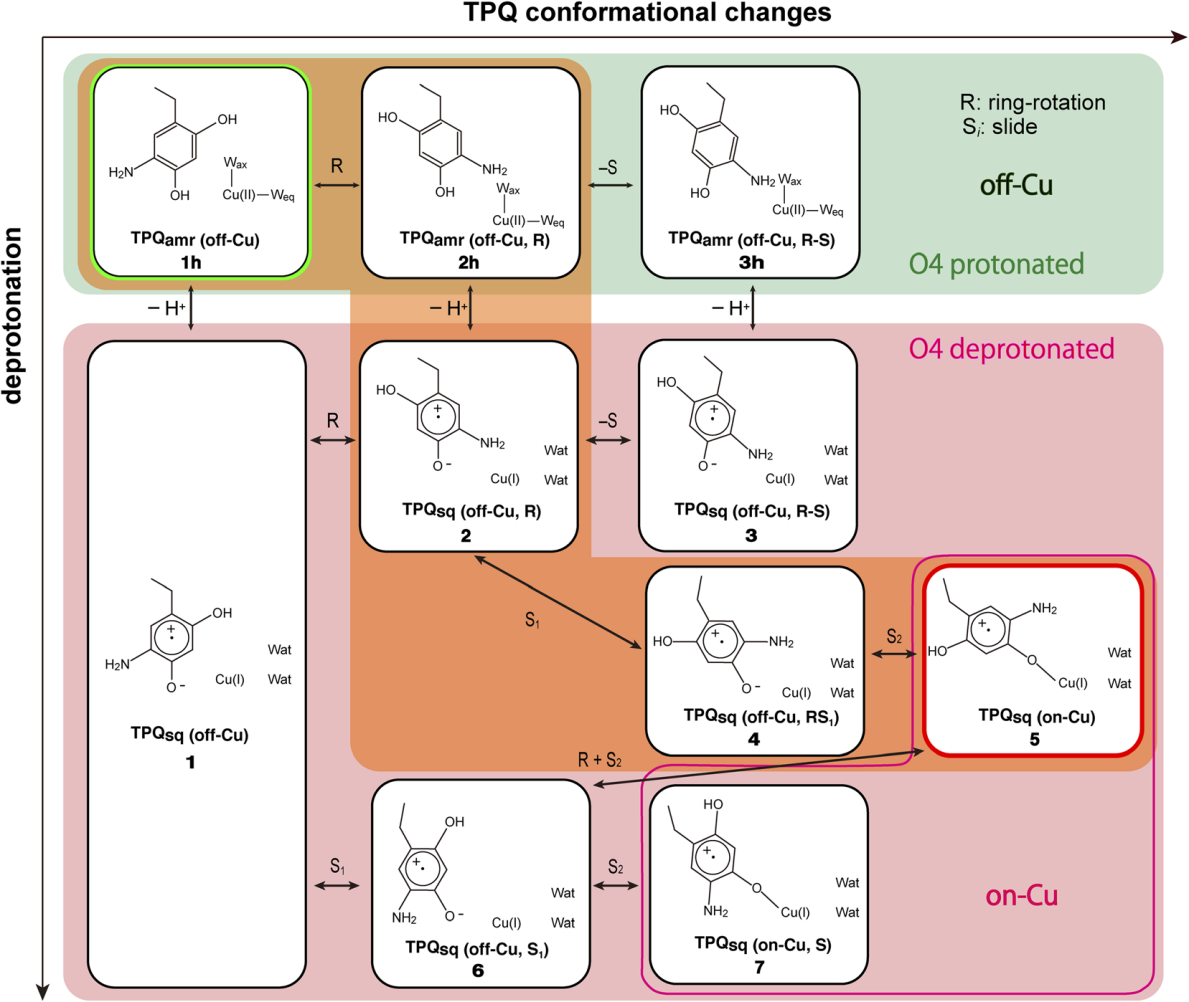
A comprehensive view of the intermediate states on which the present work is focused for the TPQ_amr_ to TPQ_sq_ transition. The letter “h” added in the labeling as in 1h–3h stands for the protonated state on O4-TPQ. The orange background highlights the main reaction pathway, whereas the green background refers to the O4-TPQ protonated state, and the red one refers to the deprotonated one.

We started our computational study by inspecting the possible protonation states of AGAO in the TPQ_amr_ and TPQ_sq_ states. We recall that the characteristic UV-vis absorption spectrum with maxima at around 440 and 470 nm^14^ originates from TPQ_sq_. This spectrum is quite different from the broad and peak less spectra of TPQ_amr_, which can be obtained as an intermediary state in solution upon anaerobic substrate reduction. The crystal structure was also determined in the presence of NaCl or NaBr by anaerobic soaking with the substrate.^14^ The halide ions are bound to the axial position of the Cu active site and act as anionic inhibitors. The characteristic absorption peaks of the TPQ_sq_ state are ascribed to an electron transfer from TPQ_amr_ to Cu(ii), leading to the formation of Cu(i). Electronic structure calculations for the different protonation states of TPQ_amr_ have shown that the protonation of both the O2 and O4 sites of TPQ is required to prevent oxidation by the Cu(ii) centre. Indeed, the energy differences for a single electron transfer from the protonated and deprotonated forms at the O4 site in TPQ_amr_, corresponding to 1h and 1 in Fig. 4, turned out to be 18.8 eV (433 kcal mol^−1^) and −32.9 eV (−758 kcal mol^−1^), respectively. From this energetics, we can infer that a single electron transfer from TPQ_amr_ to Cu(ii) is more favoured in 1 and is strictly related to the deprotonation of TPQ_amr_. Furthermore, after a structural optimization of these conformations, the Cu coordinating water molecules, W_ax_ and W_eq_, are released by the formation of Cu(i), and this also holds for the off-copper conformation. We also found that the dissociation of the water ligands (W_ax_ and W_eq_) is energetically unfavourable by more than 25 kcal mol^−1^ in the Cu(ii) state. These results suggest that the TPQ coordination to the Cu site must occur after the formation of a TPQ semiquinone radical and Cu(i). Complementary calculations have shown that O4-TPQ_amr_ deprotonation is energetically more favourable than the deprotonation of O2-TPQ_amr_ by Δ*E* = 12.3 kcal mol^−1^. Based on a synthetic model compound (aminophenol), the experimentally determined p*K*_a_ values are 9.59 and 11.62 for O4 and O2,^50^ respectively, which are consistent with the present QM/MM results. The overall picture provided by these analyses is that TPQ_amr_ is fully protonated in TPQ_amr_, and that TPQ_sq_ is formed upon an electron transfer to the Cu centre when TPQ_amr_ is deprotonated at the O4 site. Moreover, the simulations are fully consistent with the experimental detection of TPQ_amr_ when the O4 of TPQ_amr_ is protonated in the off-copper conformation.

### 3.4 Viable transition pathways of the TPQ conformational change

This paragraph is focused on the possible reaction pathways from the off-copper TPQ_amr_ to the on-copper TPQ_sq_. The TPQ conformational change involves two main motions, (i) slide and (ii) ring-rotation. The slide motion of the TPQ ring takes place when a rotation of about 50° occurs around the Cα–Cβ bond along with tilting up in a rigid-body motion of ∼20° with respect to the centre represented by the Cα atom.^14^ The ring-rotation of this same TPQ, instead, occurs by a rotation of 180° around the Cβ–Cγ bond. By considering the ordered sequence of the two main motions and the two possible rotational directions of the TPQ ring-rotation, clockwise and counter-clockwise, four alternative pathways can be identified:

(I) Clockwise-ring-rotation and slide.

(II) Counter-clockwise-ring-rotation and slide.

(III) Slide and clockwise-ring-rotation.

(IV) Slide and counter-clockwise-ring-rotation.

In a former study,^14^ we suggested that the TPQ ring needs to slide out from the off-copper position to perform a rotation and that pathway (III) is preferable to the alternative ones.^14^ Yet, a thorough and deeper analysis was not possible at that time. We filled this gap here. Specifically, we could observe that unless a conformational change of the surrounding residues occurs (Fig. 1B and S7†) these pathways are hindered to various extents. More precisely, in pathway (I), the side chains of Asn381 and Tyr384 prevent TPQ_amr_ ring-rotation. In pathway (II), the main chain of TPQ and the side chain of Val282 blocks the 2-hydroxyl group of TPQ_amr_ and the 5-amino group of TPQ_amr_, respectively. Concerning pathway (III), the main chain of Phe407 and the side chain of Tyr384 prevent ring-rotation. Finally, in pathway (IV), the side chains of Asn381 and His433 come in close contact with the TPQ moiety, thus hindering any further motion. Among these four pathways, the steric hindrance in pathway (II) seems inevitable because the hindrance during the first counter-clockwise-ring-rotation occurs within the TPQ_amr_ residue (Fig. S8A†). The steric hindrance in pathway (IV) for the counter-clockwise-ring-rotation is also unavoidable because TPQ_sq_ and the side chain of His433 are both tightly coordinated to Cu(i) (Fig. S8B†). For these reasons, we focus here on the pathways (I) and (III).

Our QM/MM simulations allowed us to identify several intermediate states with specific conformations and protonation states. All the intermediate states are sketched and shown in Fig. 4, where the protonated states of the O4-TPQ are labelled by adding a second letter “h”. 1h and 5 denote the conformations assumed upon off-copper and non-ring-rotation of one of TPQ_amr_ and the on-copper one of TPQ_sq_, respectively. These correspond to the AGAO structures determined by X-ray crystallography.^14^ States 2h and 2 have TPQ rings that are rotated with respect to 1h and 1, respectively, and take the off-copper conformation. In the middle of the slide step in pathway (I) (2 → 5 in Fig. 4), a stable intermediate state, labelled as 4, was found. In this state, the rotation angles around the Cα–Cβ bond of TPQ along 2 → 4 and 4 → 5 are 16.1° and 37.3°, respectively.

On these grounds, the TPQ slide movement can be seen as a combination of two subsequent steps, an initial slide motion (S_1_) followed by a second one (S_2_). We could also identify a state labelled as 6 generated by the slide motion from state 1 without rotating the TPQ_sq_ ring corresponding to the slide motion S_1_. The state labelled as 7 is defined as the one in which the ring takes the on-copper conformation but is turned over with respect to structure 5 and can be formed from 6*via* the S_2_ slide.

Another important issue is the moment in which the deprotonation of O4-TPQ occurs. There are three possibilities for this process to happen: the first one (A) is before the TPQ conformational change; the second one (B) is during the TPQ conformational change; and the third one (C) is after the TPQ conformational change. We remind that the deprotonation of O4-TPQ_amr_ induces the change Cu(ii) → Cu(i) and that the ligand exchange at the Cu(ii) site is energetically demanding. This result suggests that pathway (C) can be ruled out since the TPQ on-copper conformation does not proceed because of the presence of W_ax_. Similarly, in the first slide movement of the TPQ conformational change, such as for instances (III) and (IV), O4-TPQ must be displaced close to the Cu axial position in the presence of W_ax_. This means that the first ring-rotation motion of TPQ_amr_ is favoured before the TPQ_amr_ deprotonation in the case of (B). Hence, the pathways to be considered can be summarized as:

(IA): deprotonation, TPQ_sq_ clockwise-ring-rotation and slide (1h → 1 → 2 → 4 → 5).

(IB): clockwise-ring-rotation of TPQ_amr_, deprotonation, and TPQ_sq_ slide (1h → 2h → 2 → 4 → 5).

(IIIA): deprotonation, TPQ_sq_ slide, and clockwise-ring-rotation (1h → 1 → 6 → 5).

and are sketched and shown in Fig. 4.

States 3h and 3 in Fig. 4 can also be formed as alternative TPQ conformational states originating from 2h and 2 by slide motions with rotations of −88.4° and −85.1°around the Cα–Cβ bond, respectively. The conformation indicated as 3h corresponds to the TPQ_amr_ conformation observed in the X-ray crystal structure of the N381A mutant (N381A_holo/PEA_). Reaction steps 2h → 3h and 2 → 3 correspond to the negative slide motion (−S) in Fig. 4. All the states in the O4-TPQ protonated form take an electronic structure typical of TPQ_amr_, whereas all the states in the deprotonated form present the electronic structure of TPQ_sq_. The X-ray crystal structures of the WT TPQ_amr_ (PDB ID:3X3Z), N381A_holo/PEA_ (this work), and WT TPQ_sq_ (PDB ID: 3X3X) are well reproduced by states 1h, 3h^NA^, and 5, whose root-mean-square-deviation (RMSD) of the atoms inside the QM region are 0.291, 0.392, and 0.516 Å, respectively (see Table S3†).

The energies of the TPQ O4-protonated states in the WT and N381A mutant were evaluated with respect to the energies in states 1h and 1h^NA^, respectively. For O4-deprotonated states in the WT, the energies were calculated by taking as a reference the energy of state 5. This corresponds to the observed deprotonation equilibrium between TPQ_amr_ and TPQ_sq_. The validity and advantage of adopting these reference energy values, closely related to the assignment of p*K*_a_, are discussed in Section 3.17. For the N381A mutant, the relative energy in deprotonated state X^NA^ (Δ*E*(X^NA^)) was converted by referring to the energy differences in the WT. The energy correction was expressed as Δ*E*(X^NA^) = *E*(X^NA^) − *E*(1h^NA^) + *E*(1h) − *E*(5), where *E*(X^NA^) represents the total energy in state X^NA^.

### 3.5 Ring-rotation of TPQ_amr_ in the protonated form (1h → 2h)

**Fig. 5:**
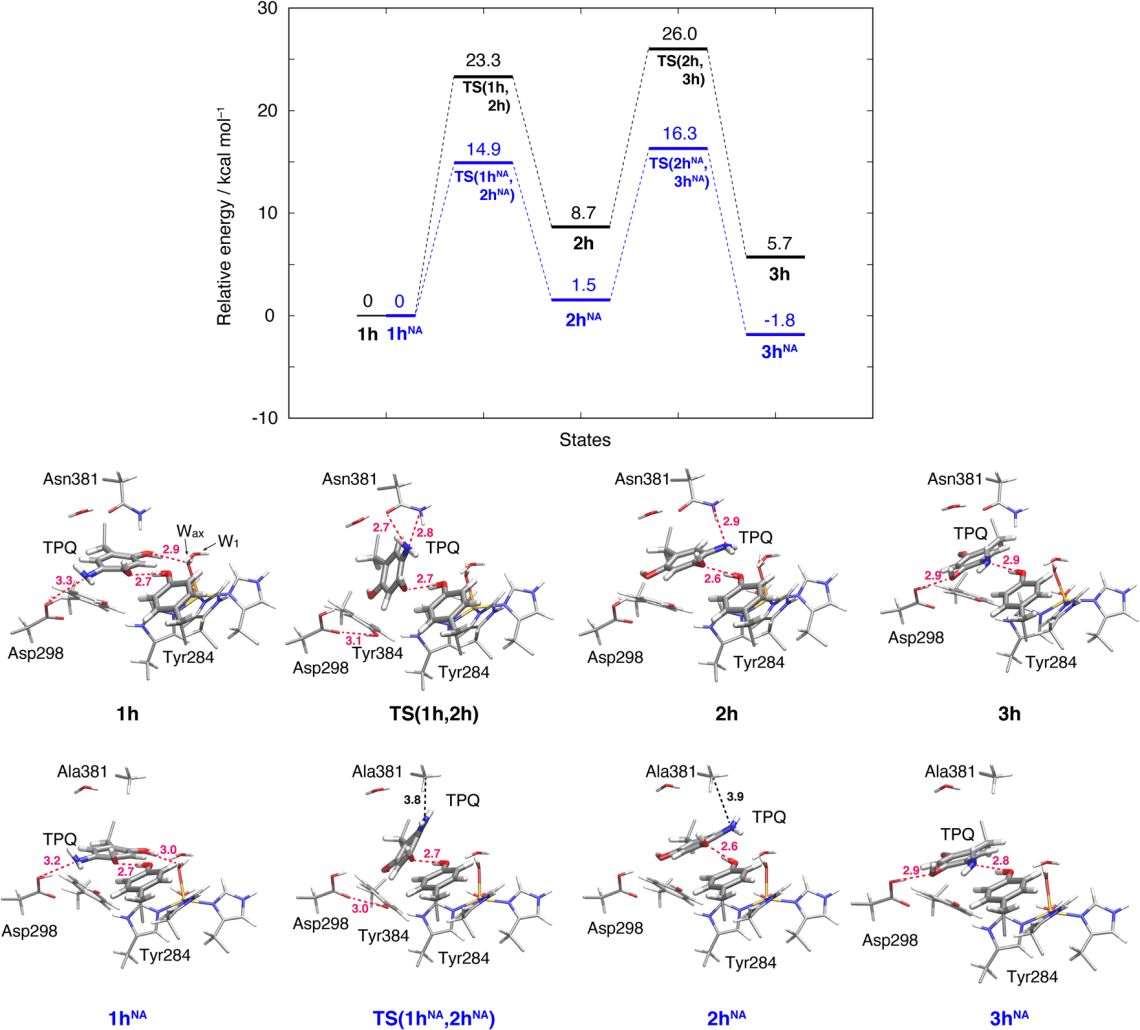
Relative energies associated with the TPQ conformational change in the protonated state. Results for the WT and the N381A mutant (NA) are shown as black and blue lines, respectively. Molecular structures of the intermediate and transition states are reported with the most important distances between heavy atoms.

The initial step of pathway (IB) is the ring-rotation of TPQ_amr_ at the off-copper position. This originates from a clockwise rotation around the Cβ–Cγ bond (1h → 2h). The calculated energy profile for the WT along with the optimized structures are shown in Fig. 5. In the initial TPQ_amr_ state (1h), the TPQ_amr_ ring forms H-bonds with Asp298, Tyr284, and W_ax_ with typical (non-hydrogen) distances of 3.3, 2.7, and 2.9 Å, respectively; the most relevant atomic distances are summarized in Table S1.† An NEB calculation was used to sample the reaction path and locate the transition state of the TPQ_amr_ ring-rotation (TS(1h,2h)). The relative energy of the TS(1h,2h) with respect to 1h is Δ*E*(TS(1h,2h)) = 23.3 kcal mol^−1^. The side chains of Asn381 and Tyr384 in TS(1h,2h) are located in the proximity of TPQ_amr_ at the distances of 2.7 and 2.7 Å, to be compared with the distances of 4.2 and 4.6 Å in 1h. These same side chains move away giving room to TPQ_amr_ rotation. The 5-amino group of TPQ_amr_ forms new H-bonds with Oδ and Nδ of Asn381 with distances of 2.7 and 2.8 Å, respectively (Table S1†). We remark that the phenol group of Tyr384, pushed by TPQ_amr_, can form one H-bond with Oδ1 of Asp298 (Table S1†). These interactions contribute to reducing the energy barrier for TPQ_amr_ ring-rotation (Fig. 4).

To evaluate the effects of the carboxamide side-chain of Asn381, we also considered the N381A mutant and inspected the conformational change on the off-copper and protonated TPQ_amr_ state (1h^NA^) to the ring-rotated form (2h^NA^) within our QM/MM computational approach (Fig. 5). For the N381A mutant model, no steric hindrance by the small side chain of Ala381 was observed, and the activation barrier of the 1h^NA^ → 2h^NA^ transition was Δ*E*(TS(1h^NA^,2h^NA^)) = 14.9 kcal mol^−1^ relative to 1h^NA^. The carboxamide group of Asn381 was expected to hinder TPQ rotation. However, as shown by our simulations, TPQ_amr_ can rotate by overcoming a rather modest barrier formed by the carboxamide group, calculated as Δ*E*(TS(1h,2h)) − Δ*E*(TS(1h^NA^,2h^NA^)) = 8.4 kcal mol^−1^, through forming a specific H-bond between the carboxamide group of Asn381 and the amino group of the TPQ ring in the WT.

The ring-rotated state (2h) is rather unstable with respect to the initial state of TPQ_amr_ (1h) by Δ*E*(2h) = 8.7 kcal mol^−1^ for the WT (Fig. 5). As W_ax_ in the hydration shell of the Cu centre is close to the 5-amino group of TPQ_amr_, the ring-rotation of TPQ around the Cβ–Cγ bond cannot be completed (compare 1h and 2h in the lower panels of Fig. 5). The dihedral angle Δ*A*(Cα, Cβ, Cγ, N5) = 142.7° is clearly lower than the expected 180° value for a full rotation. The conformation of the carboxamide group of Asn381 in 2h of the WT does not completely revert to the original position in 1h. These interactions keep the 5-amino group trapped between the carboxamide group of Asn381 and W_ax_ at distances of 2.9 and 3.3 Å, respectively, through H-bonds (Table S1†). In the N381A mutant, the state corresponding to 2h^NA^ was stabilized with respect to the WT state 2h (Δ*E*(2h^NA^) = 1.5 kcal mol^−1^) (Fig. 5). 2h^NA^ maintains a short H-bond of 2.6 Å between TPQ_amr_ and Tyr284. The molecular structures of the WT AGAO (2h) and N381A mutant (2h^NA^) are practically identical apart from the geometry of the mutated carboxamide group of Asn381 and the 5-amino group of TPQ_amr_. The destabilization of 2h can be ascribed to the interaction of TPQ_amr_ with Asn381 that has a closer position to TPQ_amr_ than in 2h^NA^.

### 3.6 Alternative TPQ_amr_ ring-rotated state in the protonated form (3h)

The TPQ_amr_ ring-rotated state of AGAO can also take an alternative conformation, labelled as 3h, in AGAO. Such a conformation of TPQ_amr_ was experimentally found by X-ray crystallography in the TPQ_amr_ form of the N381A mutant (Fig. S6†). The energy profile leading to 3h was sampled for both the WT and N381A mutant. State 3h can be formed from 2h by a slide motion of −88.4° of TPQ_amr_, and this occurs in the opposite direction with respect to the on-copper formation. Pathway 2h → 3h can be summarized as a negative slide motion (−S) (see Fig. 4). In 3h, TPQ_amr_ forms short H-bonds (2.9 Å) with Asp298 and Tyr284 (3h in Fig. 5). In spite of this H-bond stabilized 3h structure, compared to 2h, the system is still energetically located above 1h by Δ*E*(3h) = 5.7 kcal mol^−1^ (Fig. 5). Our NEB sampling of the reaction path indicates that the barrier for the 2h → 3h transition (Δ*E*(TS(2h,3h)) = 26.0 kcal mol^−1^) is higher than the one for 1h → 2h. Thus, we can infer that the formation of 3h is slower than that of 2h in the WT. In the N381A mutant, 3h^NA^ becomes more stable than the initial TPQ_amr_ state (1h^NA^) (Δ*E*(3h^NA^) = −1.8 kcal mol^−1^) and an NEB calculation showed a relatively low barrier of Δ*E*(TS(2h^NA^,3h^NA^)) = 16.3 kcal mol^−1^, which is likely to allow for rapid conversion of 2h^NA^ into 3h^NA^ in TPQ_amr_.

### 3.7 TPQ_sq_ ring-rotation in the deprotonated form (1 → 2)

The TPQ_sq_ ring-rotation of the deprotonated form is a crucial process in pathway (IA). Upon the deprotonation of O4-TPQ_amr_ in 1h, TPQ_amr_ and Cu(ii) are spontaneously converted to TPQ_sq_ and Cu(i) by single-electron transfer, and the two water molecules coordinated to the Cu(i) centre are released as discussed in Section 3.3. The hydrating water molecules can move rather easily and have enough free space to escape. For this reason, we removed redundant H_2_O in all the deprotonated states defined in the present study (1–7). This allows for easier evaluation and comparison of the relative energies of different structures in the deprotonated state.

**Fig. 6:**
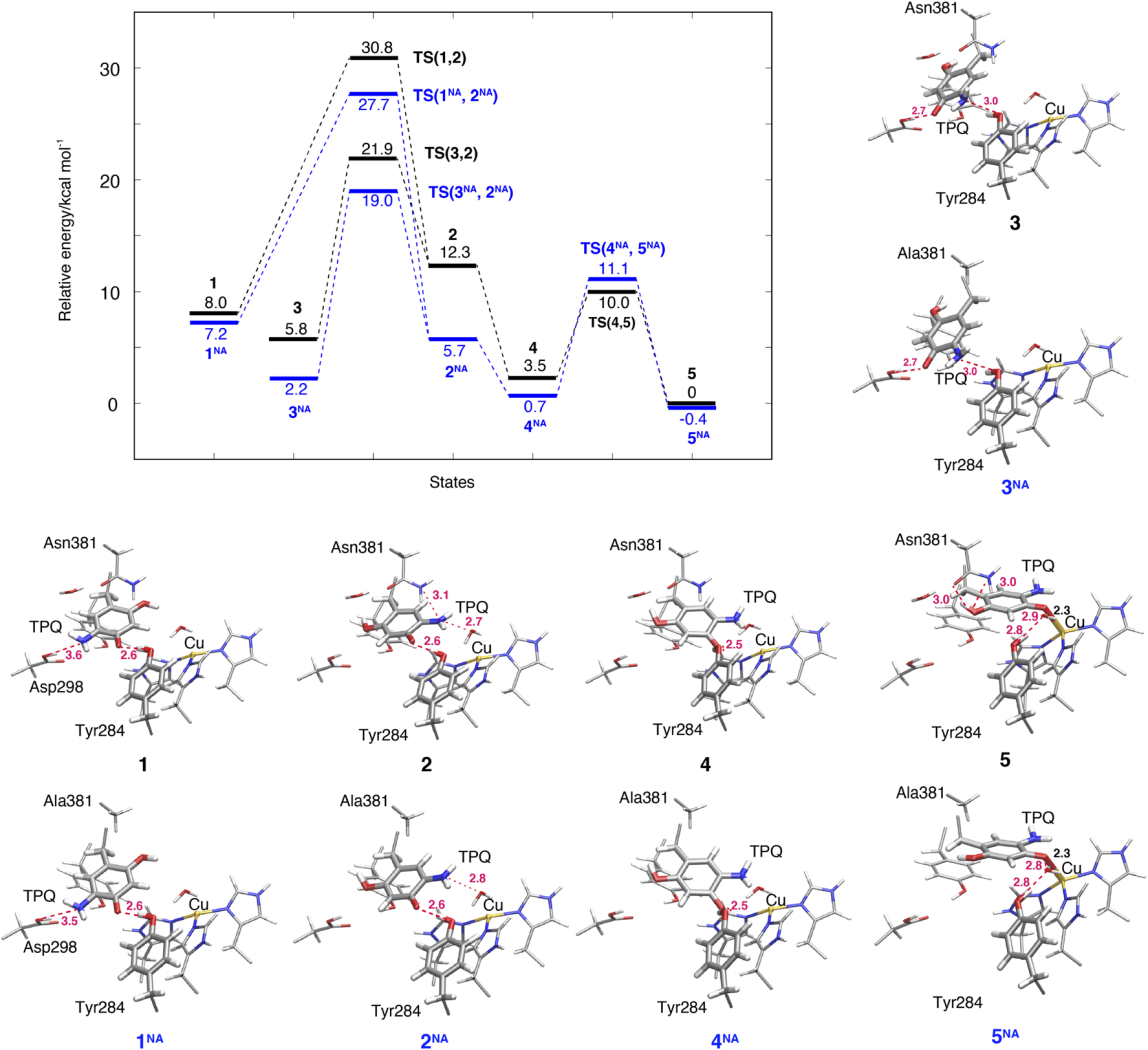
Relative energies associated with the TPQ conformational change over 1–5 states in the deprotonated form. Results for the WT and the N381A mutant (NA) are shown as black and blue lines, respectively. Molecular structures of the intermediate states are reported with the most important distances between heavy atoms.

The NEB energy profile and the main intermediate states are shown in Fig. 6 and the structure of the transition state is reported in Fig. S9 of the ESI.† In a way analogous to step 1h → 2h in the protonated form, the TPQ_sq_ ring-rotation 1 → 2 step occurs by drifting of the side chains of Asn381 and Tyr384, thereby giving room to the formation of a specific H-bond between TPQ and Asn381 in TS(1,2). As 1 and 2 have higher relative energies, Δ*E*(1) = 8.0 kcal mol^−1^ and Δ*E*(2) = 12.3 kcal mol^−1^, the NEB estimated barrier is Δ*E*(TS(1,2)) = 30.8 kcal mol^−1^. In 2, the 5-amino group of TPQ_sq_ is close to the Cu(i) site (4.6 Å), to be compared with a distance of 5.1 Å in 2h, and TPQ forms H-bonds with Asn381, Tyr284, and W1 with distances of 3.1, 2.6 and 2.7 Å, respectively. Superimposed structures of 2*vs.*2h and 1*vs.*1h shown in Fig. S10† show the small changes affecting these H-bonds. In the N381A mutant, states 1^NA^, 2^NA^ and the transition state TS(1^NA^,2^NA^) are all higher in energy than the corresponding WT states by 4 kcal mol^−1^ (Fig. 6). These energy profiles clearly suggest that TPQ ring-rotation is unfavourable in the deprotonated state of TPQ_sq_ compared to the protonated state of TPQ_amr_ in both the WT and N381A mutant.

### 3.8 TPQ_sq_ slide in the deprotonated form (3 → 2)

Within our simulation protocol, we inspected the conformational change and energetics of the deprotonated TPQ_sq_ from 3 to 2 (Fig. 6). States 3 and 3^NA^ turned out to be located at the relative energies of 5.8 and 2.3 kcal mol^−1^, respectively. The 2 and 2^NA^ structures have higher relative energy, namely 12.3 and 5.7 kcal mol^−1^, respectively. In the WT, the transition state energy for the TPQ_sq_ slide motion along the reaction path from 3 to 2 was Δ*E*(TS(3,2)) = 21.9 kcal mol^−1^. This is lower than the energies of transition states in the protonated state (Δ*E*(TS(1h,2h)) = 23.3 and Δ*E*(TS(2h,3h)) = 26.0 kcal mol^−1^). On the other hand, in the N381A mutant, the corresponding energy was Δ*E*(TS(3^NA^,2^NA^)) = 19.0 kcal mol^−1^. This energy is higher than those of transition states in the protonated state (Δ*E*(TS(1h^NA^,2h^NA^)) = 14.9 and Δ*E*(TS(2h^NA^,3h^NA^)) = 16.3 kcal mol^−1^).

### 3.9 Slide motion of the deprotonated form after the ring-rotation (2 → 4 → 5)

The steps of 2 → 4 → 5 correspond to the final slide motion in both pathways (IA) and (IB). Through this pathway, state 2 with the ring-rotated TPQ_sq_ undergoes two subsequent slide movements, S_1_ (2 → 4) and S_2_ (4 → 5), and eventually forms state 5 representing the on-copper conformation (Fig. 4). This pathway is highlighted in orange in Fig. 4. During the TPQ_sq_ slide occurring after the realization of state 2, O4-TPQ_sq_ approaches the Cu(i), as evidenced by a significant reduction of the distance from 6.3 Å to 2.1 Å in the on-copper state of 5. A stable intermediate state 4 is present before 5 (*d*(O4, Cu) = 4.1 Å). A H-bond of 2.5 Å is formed between O4-TPQ_sq_ and Tyr284 in state 4 (see Table S2†), but is absent in 5 (Fig. 6). The relative energy of state 4, Δ*E*(4) = 3.5 kcal mol^−1^, indicates higher stability with respect to the previous states 1–3. On-copper state 5 is the most stable state (Δ*E*(5) = 0 kcal mol^−1^) in the deprotonated form (Fig. 6 and Table S2†). Along the reaction path obtained from an NEB calculation, we could not locate any additional transient state for the slide motion S_1_ (2 → 4), while a TS(4,5) of 10.0 kcal mol^−1^ (Fig. 6) has to be overcome to perform the slide motion S_2_ (4 → 5). In the transition state (TS(4,5)), TPQ_sq_ is located in the proximity of His431 and characterized by distances of 3.4 Å between O4-TPQ_sq_ and Cε-His431 and 3.4 Å between C4-TPQ_sq_ and Cε-His431.

In on-copper state 5, the amino group of TPQ_sq_ is at typical H-bond distances from the main chain carbonyl of Thr403 (N5-TPQ_sq_, O-Thr403: 3.3 Å) and the Sδ atom of Met602 (N5-TPQ_sq_, Sδ-Met602: 3.3 Å) (Table S2†). The 2-OH group of TPQ_sq_ interacts with the carboxamide group of Asn381 *via* two H-bonds, specifically O2-TPQ_sq_ with Nδ-Asn381 (3.0 Å), and O2-TPQ_sq_ with Oδ-Asn381 (2.96 Å). The short H-bond between Tyr284 and TPQ_sq_ changes from a direct interaction to an indirect H-bond mediated by the bridging water molecule W1 at a distance of 2.9 Å from O4-TPQ_sq_ in 5. These H-bond interactions are also preserved in the X-ray crystal structure (PDB ID: 3X3X) and contribute to stabilizing states 5 (WT) and 5^NA^ (N381A mutant). We observed that the H-bond between O2-TPQ_sq_ and Asn381 is lost in 5^NA^, and as a result, the distance between O2-TPQ_sq_ and Cβ-Ala381 increases to 4.1 Å. The H-bond interaction between O2 of TPQ_sq_ and Asn381 in the WT can be formed only in state 5. Similar to the WT, we did not find any energy barrier for the TPQ_sq_ slide motion S_1_ (2^NA^ → 4^NA^) in the N381A mutant, while a transition state energetically located at 11.1 kcal mol^−1^ characterizes the slide motion S_2_ from 4^NA^ to 5^NA^ (Fig. 6). As with the WT, the on-copper state in the N381A mutant is most stable in TPQ_sq_ (Δ*E*(5^NA^) = −0.4 kcal mol^−1^) compared to other deprotonated states such as 4^NA^ (Δ*E*(4^NA^) = 0.7 kcal mol^−1^) and 3^NA^ (Δ*E*(3^NA^) = 2.2 kcal mol^−1^). From these results, we can infer that in both the WT and the N381A mutant, pathway 2 → 4 → 5 is favoured and represents a viable reaction channel (Fig. 4 and 6). A feature worthy of note is that TPQ_sq_ of 5^NA^ is less stable than TPQ_amr_ of 3h^NA^ in the N381A mutant due to the stabilization of 3h^NA^ as described in Section 3.6. These relative energies show that the energetic driving force required to generate TPQ_sq_ from TPQ_amr_ is reduced by ΔΔ*E*(5^NA^, 3h^NA^) − ΔΔ*E*(5, 3h) = 7.1 kcal mol^−1^ in the N381A mutant.

### 3.10 Slide motion of TPQ_sq_ after the deprotonation in the 1 → 6 pathway

**Fig. 7:**
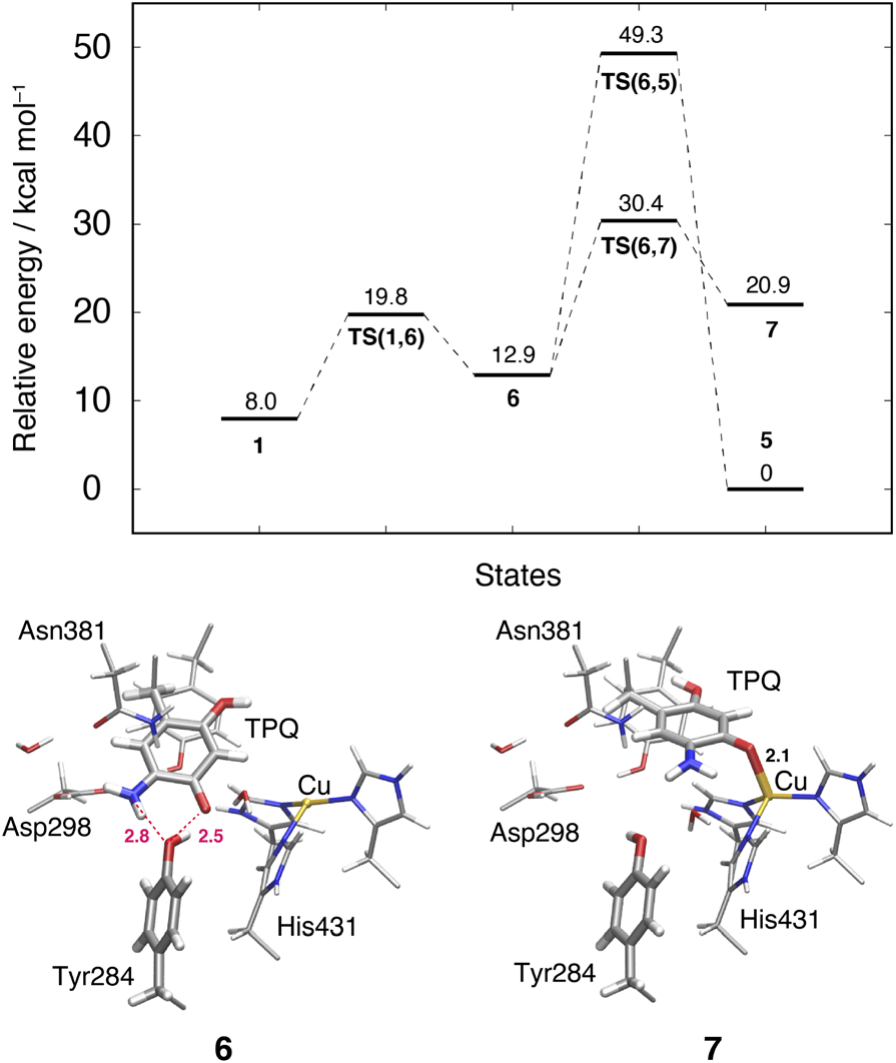
Relative energies of alternative TPQ conformational changes in the deprotonated state, including the TPQ slide motion (1 → 6 → 7) and the rotation ( → 5). The conformations of the intermediate states are reported along with the most relevant distances between heavy atoms.

This TPQ_sq_ slide motion (1 → 6) (Fig. 4) is the first conformational change occurring along pathway (IIIA). The energy profiles are shown in Fig. 7. During the TPQ_sq_ slide, the H-bond between TPQ_sq_ and Tyr284 is preserved. In state 6, after the slide motion, two H-bonds are formed, one of 2.5 Å between O4-TPQ_sq_ and O-Tyr284, and the second one of 2.8 Å between N5-TPQ_sq_ and O-Tyr284 (6 in Fig. 7 and Table S2†). The H-bond between the carboxy group of Asp298 and the amino group of TPQ_sq_ formed in 1 is lost in 6 (3.6 and 6.4 Å in 1 and 6, respectively). The relative energy of the transition state is Δ*E*(TS(1,6)) = 19.8 kcal mol^−1^, and the slide state 6 is more destabilized compared to state 1 (Δ*E*(6) = 12.9 kcal mol^−1^) (Fig. 7).

### 3.11 Subsequent TPQ_sq_ ring-rotation in the deprotonated form (6 → 5)

The ring-rotation of TPQ_sq_ (6 → 5) after the slide motion (1 → 6) is the subsequent conformational change along pathway (IIIA), whose energy profile is shown in Fig. 7. State 6 of TPQ_sq_ converts to 5 upon a rotation of the TPQ_sq_ ring around the Cβ–Cγ bond, corresponding to a change from 101.2° to −72.5° in terms of the dihedral angle Cα–Cβ–Cγ–Cδ2, thus bringing TPQ_sq_ closer not only to the side chains of Asn381 and Tyr384, but also to the main chains of Val406 and Phe407 and the side chain of His433. Compared to the atoms in the side chains, those in the main chain are more rigid and less likely to make room for the ring-rotation of the bulky TPQ_sq_. In fact, we could observe that no H-bonds are formed between TPQ_sq_ and Asn381 during the 6 → 5 transition, and TPQ_sq_ needs to be close to the side chains of Tyr384 (O-Tyr384, O2-TPQ_sq_; 2.6 Å) and Val406 (Cα-Val406, N5-TPQ_sq_; 3.1 Å) at the transition state TS(6,5). The motion of His433 also induces dissociation of the coordinated imidazole side chain from the Cu(i). Therefore, the energy of the transition state for the TPQ_sq_ ring-rotation becomes rather high, Δ*E*(TS(6,5)) = 49.3 kcal mol^−1^ (Fig. 7). Due to this large barrier, the reaction channel 6 → 5 is not easily realizable.

### 3.12 TPQ additional slide in the deprotonated form (6 → 7)

An additional slide motion of TPQ_sq_ can take place, bringing the system from state 6 to 7, in which the TPQ ring has the on-copper but ring-rotated conformation (Fig. 4 and 7). The outcome of our calculations indicated that state 7 is energetically unstable, with Δ*E*(7) = 20.9 kcal mol^−1^ (Fig. 7). From a structural standpoint, in 7, the O4-TPQ_sq_ can coordinate to the Cu(i) with a distance of 2.1 Å. Yet, TPQ_sq_ forms new H-bonds with the main chain of Asp383 and the phenol group of Tyr284, more precisely, N-Asp383, O2-TPQ_sq_ (3.5 Å) and O-Tyr284, N5-TPQ_sq_ (3.2 Å). In the transition state, the O4 atom of TPQ_sq_ is located at a position suitable to overcome the phenol group of Tyr284 (O-Tyr284, O4-TPQ_sq_ equal to 2.5 Å). The relative energy of the transition state for this slide, corresponding to the second TPQ_sq_-slide (S_2_) from 4 to 5, is Δ*E*(TS(6,7)) = 30.4 kcal mol^−1^. This value is not excessively high, but because of the higher energy of state 7 compared to state 5 (Fig. 7), step 6 → 7 does not seem to be a viable reaction channel in the AGAO catalytic cycle.

Taken together, the results presented in Sections 3.11 and 3.12 indicate that there is no low barrier route in pathway (III) with slide and clockwise-ring-rotation steps. The relative energy of the transition state (TS(6,5)) for the TPQ_sq_ ring-rotation after the slide motion is too high for the reaction to proceed, and the high relative energy of state 7 that is defined as another on-copper form (Fig. 4 and 7) after the complete TPQ_sq_ slide indicates the intrinsic instability of the system. Furthermore, attempts to find the 7 → 5 NEB pathways showed these were very unstable and are not converged to a low-barrier pathway. We can then rule out pathway (III) for the conformational change process from off-copper TPQ_amr_ to on-copper TPQ_sq_ (1h → 5).

### 3.13 TPQ_ox_ ring-rotation in the oxidized form

**Fig. 8:**
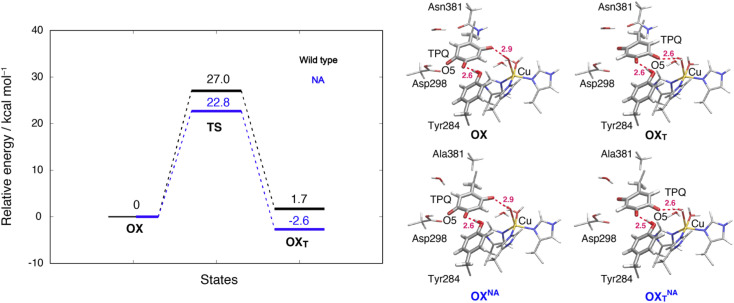
Relative energies and related conformations for TPQ rotation in the oxidized form. Results for the WT and the N381A mutant are shown in black and blue, respectively. Most relevant distances between the heavy atoms are explicitly indicated.

To inspect the TPQ_ox_ rotation, we constructed the oxidized states in the WT and in the N381A mutant by replacing the TPQ_amr_ moiety with TPQ_ox_. The 180°-flipped conformation of TPQ_ox_ was found stable in both the WT and N381A mutant. The two conformers *a* and *b* observed in the X-ray structures of N381A_holo_ were reproduced by our N381A mutant models (OX^NA^, OX_T_^NA^) within an RMSD tolerance of 0.577 and 0.610 Å, respectively (Table S4†). The role played by the residue Asn381 was investigated within our QM/MM approach and the resulting energy profiles and intermediate states for the WT and N381A mutant are shown in Fig. 8.

We noticed that the ring-rotated state of TPQ_ox_ (OX_T_) is slightly unstable with respect to the state in the unrotated conformation (Δ*E*(OX_T_) = 1.7 kcal mol^−1^) for the WT, while in the N381A mutant, OX_T_^NA^ is more stable than OX^NA^ by Δ*E*(OX_T_^NA^) = −2.6 kcal mol^−1^. The energy barrier of the N381A mutant for the TPQ_ox_ ring rotation (Δ*E*(OX_T_^NA^) = 22.8 kcal mol^−1^) is lower by 4.2 kcal mol^−1^ than that of the WT, suggesting that the process can occur at room temperature. These features are consistent with the X-ray crystal structure of N381A_holo_ where the rotated conformation of TPQ_ox_ could be observed only for the N381A mutant. The H-bond between O4-TPQ_ox_ and the side-chain OH group belonging to Tyr284 is preserved during the TPQ rotation, with length variations in the range 2.5–2.7 Å in the N381A mutant (see Table S2†). In a way analogous to TPQ_amr_, the TPQ_ox_ group is stabilized upon TPQ_ox_ rotation by forming an H-bond of 2.8 Å with the carboxamide group of Asn381 (O5-TPQ_ox_, Nδ-Asn381) in the TS(OX,OX_T_) of the WT (Table S2†). During TPQ_ox_ rotation, the methyl group of Ala381 retains its position in the N381A mutant, indicating that steric hindrance between TPQ and Asn381 is significantly reduced because of the small size of the side chain. From these results, we can infer that the rotated conformation of TPQ (OX_T_^NA^) is more favourable in the N381A mutant than in the WT. Thus, the residue Asn381 contributes to the stabilization of the unrotated TPQ_ox_ conformation and limits the unproductive rotation of the TPQ_ox_ plane in the oxidized form, whereas the TPQ ring rotation and sliding during TPQ_sq_ formation are not hindered or suppressed by the side chain of Asn381. Furthermore, Asn381 contributes to preventing an overstabilization of the rotated intermediate states in TPQ_amr_ such as 3h^NA^.

### 3.14 Comparative experimental and QM/MM analyses of TPQ_amr_ in the N381A mutant and WT

The X-ray crystal structure of N381A_holo/PEA_ provided evidence for the unusually flipped conformation of TPQ_amr_, with respect to the off-copper structure of the WT,^14^ as shown in Fig. 3B. The energy profile in Fig. 5 showed that 3h^NA^ is more stable than 1h^NA^ and 2h^NA^, and that the highest barrier connecting them is 16.3 kcal mol^−1^, which is sufficiently low to be overcome at room temperature. The stable intermediate of TPQ_amr_ switches from 1h^NA^ to 3h^NA^ in the N381A mutant, contrary to what has been observed in the protonated TPQ_amr_ of the WT, where the relative energy of 1h is significantly lower than that of 2h and 3h (Fig. 5). Among the TPQ_sq_ states in Fig. 6, product TPQ_sq_ states (5, 5^NA^) are most stable, but 3h^NA^ is more stable than 5^NA^. These energy profiles in Fig. 5 and 6 are qualitatively consistent with the fact that only the TPQ_amr_ state could be observed in the N381A mutant by using UV-vis spectra and X-ray crystallography.

### 3.15 Role of Asn381

In principle, the rather high degree of rigidity of the carboxamide group of Asn381 would jeopardize the rotation of TPQ_amr_. Nonetheless, we could show that this is not necessarily true. In fact, the Asn381 carboxamide group can be displaced and give room to TPQ_amr_ to rotate in the WT, since the energy contributions of the group to the rotation barriers do not exceed 10 kcal mol^−1^, as assessed by the decrease in the TS energy levels by the N381 mutation (Fig. 5). The energy profile of the N381A mutant indicates that the ring-rotated state 3h^NA^ of TPQ_amr_ is more stable than the non-ring-rotated one 1h^NA^ (Δ*E* = −1.8 kcal mol^−1^, Fig. 5). This indicates that the intermediate state during the catalytic reaction of the N381A mutant is trapped at TPQ_amr_ (3h^NA^) suppressing the accumulation of TPQ_sq_ during the reductive half-reaction. The net effect is that Asn381 destabilizes the nonproductive TPQ ring-rotated conformations of both TPQ_ox_ and TPQ_amr_ states, thus promoting only the desired catalytic reaction. We can also remark that the ring-rotation of TPQ in the off-copper conformation is allowed by Asn381.

### 3.16 Role of TPQ_sq_ in the AGAO catalytic cycle

For AGAO, a fast oxidation of Cu(i)-TPQ_sq_*via* a direct reaction with O_2_ was detected from kinetics analysis of the transient absorption spectra.^14,27^ Nonetheless, to react with O_2_, a conformational change from the on-copper to an off-copper of TPQ_sq_ was required.^25^ The computed energy profile in the deprotonated form (Fig. 6) suggests that the on-copper conformation (5) is easily converted into a transient off-copper conformation (4) in TPQ_sq_, and a direct coordination of O_2_ to the Cu(i) axial position becomes possible in the oxidative half-reaction. The kinetics of TPQ_sq_ oxidation in AGAO could not be explained in terms of an outer-sphere mechanism,^14,27^ in which TPQ_amr_ reacts with O_2_ without forming TPQ_sq_ and Cu(i). All these results support the notion that TPQ_sq_ with a large conformational change and Cu(ii) are exploited in AGAO.

Conversely, for BSAO, HPAO-1 and HPAO-2, no TPQ conformational change has been evidenced and TPQ_sq_ is not formed during the catalytic cycle. The catalytic rates of Co(ii)-substituted CAOs are similar to the wild-type copper containing one for these CAOs,^51–53^ while AGAO, PSAO and ECAO significantly reduce their catalytic activities to 2.2%, 4.7% and 12%, respectively, upon Co(ii)-substitution.^12,54,55^ These results suggest that the former CAOs undergo oxidative half-reactions *via* an outer-sphere mechanism, which is different from the inner-sphere mechanism expected in the latter CAOs.

### 3.17 Deprotonation of TPQ_amr_ in pathway (I)

The deprotonation process that could occur in pathway (I) remains elusive in our QM/MM-based simulations. To encompass the deprotonation issue, we can resort to experimental data that is provided by the pH dependency of the equilibrium between TPQ_amr_ and TPQ_sq_. When AGAO is anaerobically reduced by high-affinity substrates such as 2-PEA, the pH dependency shows that two ionizable groups characterized by p*K*_a1_ = 5.96 and p*K*_a2_ = 7.74 are involved in the equilibrium shift.^14^ The p*K*_a1_ and p*K*_a2_ are ascribed to 5-NH_2_ of TPQ_amr_ and O4-TPQ_sq_,^14^ on the basis of the p*K*_a_s of model compounds, 5.88 (ref. 50) and 6.39 (ref. 56), respectively, although ambiguity still remains for the difference between the experimental p*K*_a_ value of O4-TPQ_amr_ and that of the model compound (9.59).^14^

The 5-NH_2_ group and O2-TPQ_amr_ interact with the side-chain carboxyl group of Asp298 and the Cu(ii)-coordinated W_ax_, respectively, in the off-copper conformation (1h) (Table S2†). The ring-rotation to convert 1h to 2h changes the environments of both 5- and 2-groups simultaneously: The 5-NH_2_ group interacts with W_ax_, Tyr284, and Asn381 through hydrogen bonds, whereas the O2-TPQ_amr_ lacks a hydrogen bond and comes closer to the Asp298 side chain (Table S2†). Thus, the electrostatic environments of TPQ_amr_ in 2h would significantly perturb the electronic state of TPQ_amr_. It presumably results in the decrease of the p*K*_a_ of O4-TPQ_amr_, although the O4-TPQ_amr_ forms a hydrogen bond with Tyr284 (Table S2†). We predicted that the p*K*_a_ of O4-TPQ_amr_ in the off-copper conformation (1h) is similar to that indicated by the TPQ_amr_ model compound (p*K*_a_ = 9.59)^50^ and that after the ring-rotation, the p*K*_a_ of O4-TPQ_amr_ (2h) reduces to the experimentally determined value (p*K*_a_ = 7.74), which is comparable to that of the O4-TPQ_sq_ (p*K*_a_ = 6.39) found in the TPQ_sq_ model compound^56^ as described previously.^14^ The present QM/MM calculation also reveals that the ring-rotation promotes the deprotonation of O4-TPQ_amr_. We can infer that the deprotonation of 2h (2h → 2) is more favourable by 4.4 kcal mol^−1^ in comparison with that of 1h (1h → 1) (Fig. 5 and 6).

For state 3h, it is expected that its deprotonation to 3 is preferable in energy if the conformational change of the −S movement from 2h to 3h becomes a lower energy barrier process. The present energy barrier (26.0 kcal mol^−1^) of 2h → 3h suggests an unfavourable pathway (Fig. 5, see Section 3.6). However, the deprotonation of 3h (3h → 3) is more favoured by 7.9 kcal mol^−1^ than that of 1h (1h → 1) (Fig. 5 and 6). The formation of 2h represents the most direct pathway. However, the alternative and more indirect pathway, visiting also the conformations 3h and 3 is still viable along the route of 1h → 2h → 3h → 3 → 2 → 4 → 5 (Fig. 4), although this reaction channel is characterized by a slower kinetics in the 2h → 3h transition. These energy profiles suggest a possibility that the pathway *via*3h may be utilized with different substrates or in other CAOs.

The assignment of p*K*_a2_ = 7.74 to O4-TPQ_sq_ provides an important basis for discussing the overall reaction pathway. States 1h and 5, which are the most stable configurations in TPQ_amr_ and TPQ_sq_, respectively, are the main species detected at pH values around 7.74 ref. 14. Thus, at pH 7.74, we can directly compare the energy profiles of TPQ O4-protonated states (Fig. 5) and TPQ O4-unprotonated states (Fig. 6 and 7), by adjusting the relative energies of 1h and 5, since 1h and 5 + H^+^ have the same chemical potential at this pH. At other pH values, a similar comparison can be done by offsetting Fig. 6 and 7 upward by 1.36 (7.74 − pH) kcal mol^−1^ relative to Fig. 5. Based on the profiles overlaid in this way we can deduce the plausible overall reaction pathway in the WT as described in Section 4.

## 4 Conclusions

The possible reaction pathways characterized by the TPQ conformational change during the TPQ_amr_ to TPQ_sq_ transition were investigated for the AGAO system providing an atomistic insight into the detailed process and a thorough analysis of the associated energetics. From the molecular structures of the surrounding residues and TPQ_amr_, and deprotonation time of TPQ_amr_, we have narrowed down the most viable reaction pathways into three, all of them including the clockwise-ring-rotation of TPQ, *i.e.*, (IA) 1h → 1 → 2 → 4 → 5, (IB) 1h → 2h → 2 → 4 → 5, and (IIIA) 1h → 1 → 6 → 5.

**Fig. 9:**
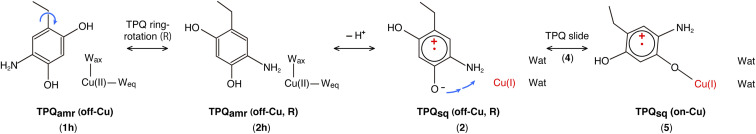
Reaction mechanism of the TPQ_amr_ → TPQ_sq_ transition obtained in the present study.

The first and third pathways (IA and IIIA) have higher energy barriers of more than 30 kcal mol^−1^ for the 1 → 2 and 6 → 5 transitions. Therefore, the most favourable pathway is (IB): the TPQ_amr_ → TPQ_sq_ reaction in the WT AGAO proceeds through the TPQ_amr_ ring-rotation, deprotonation of O4H-TPQ_amr_, and TPQ_sq_ slide. This reaction mechanism is summarized in Fig. 9. The reaction pathway *via*3h (1h → 2h → 3h → 3 → 2 → 4 → 5) can be regarded as a more indirect route in the WT AGAO compared to the direct pathway (IB), and this pathway *via*3h is not preferable, at least under the condition of the present theoretical model. In the N381A mutant AGAO, the main reaction pathway is the indirect route *via*3h^NA^: 1h^NA^ → 2h^NA^ (⇄ 3h^NA^) → 2^NA^ → 4^NA^ → 5^NA^. The most stable state is 3h^NA^ and the highest energy barrier along this pathway is the 3h^NA^ → 2h^NA^ step with an energy barrier of 18.1 kcal mol^−1^ (Δ*E*(2h^NA^, 3h^NA^) − Δ*E*(3h^NA^)). The actual TPQ_amr_ ring-rotation state (3h^NA^) was determined from the X-ray crystal structure of the N381A mutant.

The large conformational change of the TPQ_amr_ ring in the WT AGAO can only be permitted before TPQ_amr_ deprotonation, and this conformational change is essential for stable formation of TPQ_sq_ in AGAO. On the other hand, for TPQ_ox_, the TPQ ring flip partially occurs in the N381A mutant, but it is absent in the WT. The TPQ ring-flipped conformation in TPQ_ox_ (OX_T_^NA^) and the related energetics are determined by a synergy of the X-ray crystal structure and QM/MM calculation for the N381A mutant.

This seems reasonable because the unusually large conformational change taking place in the TPQ ring-rotation and sliding motions in TPQ_ox_ would reduce the probability of the nucleophilic attack of the substrate amines toward the O5 carbonyl of TPQ_ox_ being directed to the substrate-binding pocket. This provides a clear picture of the ingenious role played by Asn381 in directing the reaction pathway starting either from TPQ_ox_ or from TPQ_amr_.

Pathway (IB) is also consistent with the results of transient kinetics experiments of the AGAO catalytic reaction.^14^ Our previous study has demonstrated that the rate constant of the TPQ_amr_ → TPQ_sq_ step (*k*_+4_ = 39 s^−1^ at 4 °C) is much lower than the large value that is predicted for electron transfer from a donor to an acceptor apart from a short distance (the distances from O4 and O2-TPQ_amr_ to the Cu centre = 6.7 and 4.8 Å, respectively, in AGAO and PDB ID: 3X3Z). In fact, temperature-jump relaxation studies have shown that the rate constant of the electron transfer (*k*_ET_) is 20 000 s^−1^ for PSAO.^57^ In addition, the dependence of *k*_±4_ on solvent viscosity in AGAO suggested the presence of a large conformational change.^14^ Therefore, we have predicted that the electron transfer is gated by the conformational change. The present study clearly showed that the TPQ ring-rotation at the off-copper position, followed by TPQ deprotonation, is a relevant change for electron transfer from TPQ_amr_ to TPQ_sq_. Interestingly, the active site of PSAO^9^ contains two dissociable residues Lys296 and Glu412 located close to TPQ, and the former is hydrogen-bonded with O4-TPQ (Fig. S11†). These dissociable side chains may significantly reduce the p*K*_a_ of O4-TPQ_amr_ in the off-copper position, facilitating deprotonation. Furthermore, in PSAO, a less bulky residue, Asn389, is located under the TPQ ring at the position corresponding to Tyr384 that restricts the ring-rotation in AGAO, while the highly conserved Asn residue (corresponding to Asn381 in AGAO), Asn386, is located on the TPQ ring (Fig. S11†). This difference is expected to enable TPQ ring-rotation through a low energy barrier. As a result, PSAO mainly has the ring-rotated conformation of TPQ_ox_ in the X-ray crystal structure^9^ (Fig. S11†). Despite the unreactive conformation, the distinct features of the active-site structure may facilitate the deprotonation of O4-TPQ_amr_ in PSAO, resulting in very fast electron transfer and efficient TPQ_sq_ formation.

The reaction mechanism of AGAO elucidated here implies that the amino acid residues in the active site, Asn381 and Tyr384, as well as the TPQ cofactor are remarkably dynamical and flexible. Nonetheless, they can reorient and rearrange depending on the multistep reaction channel and play a major role in promoting the catalytic reaction. Besides AGAO, other enzymes such as dihydrofolate reductase, flavin-dependent *N*-hydroxylase, cytochrome *c* oxidase, and electron transfer flavoprotein undergo large conformational changes during their own catalytic reactions.^3,58–60^ Their structural changes are utilized in their specific reaction steps to stabilize intermediates, to eject the spent NADP^+^, and to trigger proton transfer and rapid electron transfer. From a general standpoint, important conformational changes are a general paradigm rather ubiquitous for realizing efficient biological functions.

We stress the fact that the present study aims at providing insights into the reaction pathways of TPQ_sq_ formation in AGAO, along with accurate evaluations of their energy profiles. This paves the route to forthcoming studies of the structural flexibilities for the whole AGAO. We are confident that the present work will stimulate additional studies exploiting the joint use of molecular dynamics simulations and experimental reaction kinetics.

## Data availability

Crystallographic data of the N381A AGAO have been deposited in the Protein Data Bank under IDs 7WIR (holo, TPQ_ox_^N381A^) and 7WIS (substrate-reduced, TPQ_amr_^N381A^). The datasets supporting this article have been uploaded as part of the ESI.†

## Author contributions

M. S., T. M., M. B., Y. S., H. H., and T. O. participated in research design. M. S. conducted the theoretical calculations, and T. M. and T. O. conducted the experiments. All authors contributed to perform the data analysis and to write the manuscript.

## Conflicts of interest

There are no conflicts to declare.

## Supplementary Material

SC-013-D2SC01356H-s001
