# Supplementary material for: Molecular mechanism of a large conformational change of the quinone cofactor in the semiquinone intermediate of bacterial copper amine oxidase
Source: Chem Sci. 2022 Aug 23;13(36):10923–38. doi: 10.1039/d2sc01356h (PMC9491219; doi:10.1039/d2sc01356h)
Supplement: SC-013-D2SC01356H-s001 [file SC-013-D2SC01356H-s001.pdf]

Electronic Supplementary Material (ESI) for Chemical Science.
This journal is © The Royal Society of Chemistry 2022

Supplementary Information

Molecular mechanism of a large conformational change of the quinone cofactor in the semiquinone intermediate of bacterial copper amine oxidase

Mitsuo Shoji,* Takeshi Murakawa, Shota Nakanishi, Mauro Boero, Yasuteru Shigeta, Hideyuki Hayashi, Toshihide Okajima

*Corresponding author: Mitsuo Shoji, mshoji@ccs.tsukuba.ac.jp

Table of Contents

Experimental Details

Enzyme Preparation

Measurement of Rate of TPQ biogenesis

Analysis of Transient Kinetics of Catalytic Reaction

Table S1. Statistics of data collection and crystallographic refinement.

Table S2. Most relevant atomic distances between heavy atoms in the simulated structures.

Table S3. Comparison of the QM region in our simulations with respect to the X-ray crystal structures in TPQ_{amr} and TPQ_{sq}.

Table S4. Comparison of the QM region in our simulations with respect to the X-ray crystal structures in the oxidized form.

Figure S1. SDS-PAGE analysis of AGAO.

Figure S2. Variations in the UV/vis spectra during TPQ biogenesis of the N381A mutant and the WT of AGAO.

Figure S3. Transient spectral changes during the reductive half-reaction of the N381A mutant and the WT of AGAO with 2-PEA.

Figure S4. UV/vis absorption spectra of the N381A crystals: $N381A_{holo}$ and $N381A_{holo/PEA}$.

Figure S5. Structure of the cofactor in the N381A mutant of AGAO.

Figure S6. Structure of the active site of the of the TPQ_{amr} intermediate of the N381A mutant formed by reaction with 2-PEA.

Figure S7. Amino acid residues surrounding the TPQ_{amr} (TPQ382).

Figure S8. Steric hindrance acting during the TPQ conformational changes.

Figure S9. Molecular structures of the transition states.

Figure S10. Superimposed structures realized upon the deprotonation of O4H-TPQ: **1**/**1h**, **2**/**2h** and **3**/**3h**.

Figure S11. Active-site structure of the copper amine oxidase in *Pisum savitum*.

References

Atomic coordinates of the QM region in the intermediate and transition states are provided in a standard XYZ format and in Å units.

Experimental Details

Enzyme Preparation.

Site-specific mutagenesis of Asn381 into Ala was obtained by using a QuikChange Site-Directed Mutagenesis Kit (STRATAGENE) following the manufacturer's instructions with the pairs of mutually complementary primers (sense strand: N381A(+), 5'-CTTCACCACTATCGGCGCCTACGACTACGGCTTC-3'; antisense strand: N381A(–); 5'-GAAGCCGTAGTCGTAGGCGCCGATAGTGGTGAAG-3') containing mismatching bases (underlined) for the mutated codons and a plasmid pEPO-02 [S1] encoding the WT enzyme as a template.

The WT and the N381A mutant of AGAO were expressed in *Escherichia coli* CD03 in a Cu-depleted medium and purified to homogeneity in the Cu^{2+}/TPQ-less apo form described in Ref. [S2]. The Cu^{2+}- and TPQ-containing holo form was prepared as reported in Ref. [S3]. In the present study, the TPQ biogenesis in the N381A mutant was done by aerobic incubation for a considerably longer time (3 weeks at 4 °C) than that of the WT (about 15 h at 4 °C). The protein concentrations were spectrophotometrically determined using extinction coefficients at 280 nm of 12.3 and 13.2 for the 1% (w/v) solutions of the apo and holo forms, respectively [S2] and expressed as molar concentrations of the subunit, unless otherwise stated.

Measurement of Rate of TPQ Biogenesis.

The rate of the TPQ biogenesis was spectrophotometrically determined in the reaction mixture containing the 0.1 mM apo AGAO subunit (WT and N381A mutant) and 0.25 mM $CuSO_4$ in 50 mM HEPES buffer at pH 6.8. The reaction was done at 30 °C under atmospheric conditions, and the absorption spectra in the wavelength region 300–700 nm were recorded every 30 s using an Agilent 8453 photodiode-array spectrophotometer (Agilent Technologies, Inc.). The observed rate constants (k_{obs}) for the TPQ biogenesis were calculated by fitting the time-depended absorbance changes at 480 nm or 495 nm for the WT and N381A enzymes, respectively, to single exponential curves using IGOR Pro (Wave Metrics, Inc.).

Analysis of Transient Kinetics of Catalytic Reaction.

Transient changes of the absorption spectra of AGAO during the reductive half-reaction are monitored at 15 °C using a stopped-flow spectrometer (Applied Photophysics, Ltd.) with all lines and reservoir cylinders maintained under fully anaerobic conditions. Typically, equal volumes (about 30 μL each) of the enzyme solution (0.18 mM subunit of the WT or N381A mutant AGAO in 50 mM HEPES buffer, pH 6.8) and substrate solution (8 mM 2-PEA) were mixed in a mixing cell (volume, 20 μL) triggered with an N_2-gas piston; the mixing dead time was 2.3 ms at the N_2-gas pressure of 500 kPa. The UV/visible absorption spectra were recorded every 2.56 ms over the wavelength range of 250–800 nm. To avoid spectral changes induced by the oxidative half-reaction,

both the enzyme and the substrate solutions were kept in an anaerobic SGV-65V glove box (AS ONE corporation) filled with 99.999% Ar gas for at least 12 h before the stopped-flow measurements.

Table S1. Statistics of data collection and crystallographic refinement

Proteins<br>(PDB code)	N381A$_{holo}$<br>(7WIR)	N381A$_{holo/PEA}$<br>(7WIS)
data collection		
wavelength (Å)	0.90	0.90
space group	*C*2	*C*2
unit cell dimensions		
a, *b*, *c* (Å)	192.97, 63.02, 153.01	191.93, 63.47, 185.10
β (deg)	117.63	117.30
no. of observations	964630	492849
no. of unique reflections	265999	130556
resolution range (Å)	22.45 – 1.50<br>(1.58 – 1.50)[a]	26.56 – 1.90<br>(2.00 – 1.90)
multiplicity	3.6 (3.5)	3.8 (3.7)
completeness (%)	99.0 (97.1)	98.0 (97.1)
R_{merge} (%)[b]	9.1 (47.3)	12.9 (46.1)
refinement statistics		
resolution range (Å)	22.45 – 1.50	26.36 – 1.90
no. of solvent atoms	1540	1231
rms deviation from ideal values		
bond length (Å)	0.012	0.007
bond angles (deg)	1.504	1.105
R factor (%)[c]	16.10	16.26
R_{free} (%)[d]	18.10	19.67
Ramachandran plot		
favored/allowed/outlier (%)	96.7/2.9/0.4	95.8/4.0/0.2

[a]The numbers in parentheses indicate the value for the highest resolution shell. [b]$R_{merge} = \Sigma hkl \Sigma_i |I_i(hkl) - \langle I(hkl) \rangle| / \Sigma hkl \Sigma_i I_i(hkl)$, where $I_i(hkl)$ is an individual intensity measurement, and $\langle I(hkl) \rangle$ is the average intensity for this reflection. [c]$R = \Sigma ||F_o| - |F_c|| / \Sigma |F_o|$. [d]$R_{free}$ is an *R* factor of the refinement evaluated for 5% of reflections that were excluded from the refinement.

Table S2. Most relevant atomic distances between heavy atoms in the simulated structures.

State	Atom 1	Atom 2	Distance / Å
**1h**	Oδ1-Asp298	N5-TPQ_{amr}	3.33
	Oη-Tyr284	O4-TPQ_{amr}	2.67 (HB[a])
	O-Wat_{ax}	O2-TPQ_{amr}	2.92 (HB)
**$1h^{NA}$**	Oδ1-Asp298	N5-TPQ_{amr}	3.20 (HB)
	Oη-Tyr284	O4-TPQ_{amr}	2.67 (HB)
	O-Wat_{ax}	O2-TPQ_{amr}	3.02 (HB)
**TS (1h, 2h)**	Oδ-Asn381	N5-TPQ_{amr}	2.71 (HB)
	Nδ-Asn381	N5-TPQ_{amr}	2.75 (HB)
	Oη-Tyr384	Oδ1-Asp298	3.07 (HB)
	Oη-Tyr284	O4-TPQ_{amr}	2.68 (HB)
	O2-TPQ_{amr}	Oη-Tyr384	2.67
**TS ($1h^{NA}$, $2h^{NA}$)**	N5-TPQ_{amr}	Cβ-Ala381	3.75
	Oη-Tyr384	Oδ1-Asp298	2.96 (HB)
	Oη-Tyr284	O4-TPQ_{amr}	2.66 (HB)
	O2-TPQ_{amr}	Oη-Tyr384	2.67
**2h**	O-Wat_{ax}	N5-TPQ_{amr}	3.32
	Oη-Tyr284	O4-TPQ_{amr}	2.63 (HB)
	Oη-Tyr284	N5-TPQ_{amr}	3.17 (HB)
	N5-TPQ_{amr}	Nδ-Asn381	2.88 (HB)
**$2h^{NA}$**	O-Wat_{ax}	N5-TPQ_{amr}	3.14
	Oη-Tyr284	O4-TPQ_{amr}	2.60 (HB)
	N5-TPQ_{amr}	Cβ-Ala381	3.94
**TS (2h, 3h)**	Oη-Tyr284	N5-TPQ_{amr}	2.60
	Oη-Tyr284	O4-TPQ_{amr}	3.31
	Nδ-Asn381	N5-TPQ_{amr}	3.06 (HB)
**TS ($2h^{NA}$, $3h^{NA}$)**	Oη-Tyr284	N5-TPQ_{amr}	2.67
	Oη-Tyr284	O4-TPQ_{amr}	3.69
	Cβ-Ala381	N5-TPQ_{amr}	4.95
**3h**	Oδ1-Asp298	O4-TPQ_{amr}	2.91 (HB)
	Oη-Tyr284	N5-TPQ_{amr}	2.88 (HB)
	Oδ-Asn381	N5-TPQ_{amr}	3.70
**$3h^{NA}$**	Oη-Tyr284	N5-TPQ_{amr}	2.84 (HB)
	Oδ1-Asp298	O4-TPQ_{amr}	2.91 (HB)
**1**	Oδ1-Asp298	N5-TPQ_{sq}	3.59
	Oη-Tyr284	O4-TPQ_{sq}	2.60 (HB)
**1NA**	Oδ1-Asp298	N5-TPQ_{sq}	3.49

	Oδ2-Asp298	Oη-Tyr384	3.25 (HB)
	Oη-Tyr284	O4-TPQ_{sq}	2.61 (HB)
**TS (1, 2)**	Oδ1-Asp298	O2-TPQ_{sq}	3.47
	Oδ2-Asp298	Oη-Tyr384	2.95 (HB)
	Oη-Tyr284	O4-TPQ_{sq}	2.70 (HB)
	Oδ-Asn381	N5-TPQ_{sq}	2.82 (HB)
	Nδ-Asn381	N5-TPQ_{sq}	2.96 (HB)
**TS (1NA, 2NA)**	Oδ1-Asp298	O2-TPQ_{sq}	4.93
	Oδ2-Asp298	Oη-Tyr384	2.98 (HB)
	Oη-Tyr284	O4-TPQ_{sq}	2.66 (HB)
	O2-TPQ_{sq}	Oη-Tyr384	2.55
	Cβ-Ala381	N5-TPQ_{sq}	4.08
**2**	N5-TPQ_{sq}	Nδ-Asn381	3.10
	Oη-Tyr384	Oδ2-Asp298	3.14 (HB)
	Oη-Tyr284	O4-TPQ_{sq}	2.57 (HB)
	Oδ1-Asp298	O2-TPQ_{sq}	3.38
	Oη-Tyr384	N5-TPQ_{sq}	3.30
**2NA**	Oη-Tyr284	O4-TPQ_{sq}	2.57 (HB)
	Oδ1-Asp298	O2-TPQ_{sq}	3.40
	Oδ2-Asp298	Oη-Tyr384	3.16 (HB)
	Cβ-Ala381	N5-TPQ_{sq}	4.69
	N5-TPQ_{sq}	Oη-Tyr384	3.29
**3**	Oδ-Asn381	N5-TPQ_{sq}	3.63
	Oη-Tyr284	N5-TPQ_{sq}	3.00 (HB)
	Oδ1-Asp298	O4-TPQ_{sq}	2.73 (HB)
	Oδ2-Asp298	Oη-Tyr384	3.10 (HB)
**3NA**	Oδ1-Asp298	O4-TPQ_{sq}	2.74 (HB)
	Oδ2-Asp298	Oη-Tyr384	2.98 (HB)
	Oη-Tyr284	N5-TPQ_{sq}	2.97 (HB)
**4**	Oδ2-Asp298	Oη-Tyr384	3.22 (HB)
	Oδ1-Asp298	O2-TPQ_{sq}	4.16
	Oη-Tyr284	O4-TPQ_{sq}	2.50 (HB)
	O-Wat1	N5-TPQ_{sq}	2.96 (HB)
**4NA**	Oη-Tyr284	O4-TPQ_{sq}	2.50 (HB)
	Oδ1-Asp298	O2-TPQ_{sq}	3.97
	Oδ2-Asp298	Oη-Tyr384	3.21 (HB)
	Cβ-Ala381	O2-TPQ_{sq}	5.81
	N5-TPQ_{sq}	O-Wat1	2.92 (HB)

	N5-TPQ_{sq}	Cu	3.29
**TS (4, 5)**	Oη-Tyr284	O2-TPQ_{sq}	5.18
	Oη-Tyr284	O4-TPQ_{sq}	2.73
	Oδ1-Asp298	O2-TPQ_{sq}	4.39
	Oδ-Asn381	O2-TPQ_{sq}	3.15 (HB)
	Oη-Tyr284	O-Wat1	3.29 (HB)
	O2-TPQ_{sq}	O-Wat1	2.68 (HB)
	O4-TPQ_{sq}	Cu	3.79
**TS (4NA, 5NA)**	Oη-Tyr284	O4-TPQ_{sq}	2.71
	Oδ1-Asp298	O2-TPQ_{sq}	4.18
	Cβ-Ala381	O2-TPQ_{sq}	5.64
	Oη-Tyr284	O-Wat1	3.22 (HB)
	O2-TPQ_{sq}	O-Wat1	2.71 (HB)
	O4-TPQ_{sq}	Cu	3.94
**5**	Oδ1-Asp298	O2-TPQ_{sq}	6.00
	Oδ2-Asp298	Oη-Tyr384	3.11 (HB)
	Oδ-Asn381	O2-TPQ_{sq}	2.96
	Nδ-Asn381	O2-TPQ_{sq}	3.04 (HB)
	Oη-Tyr284	O-Wat1	2.75 (HB)
	O2-TPQ_{sq}	O-Wat1	2.88 (HB)
	O4-TPQ_{sq}	Cu	2.27 (CD[b])
**5NA**	Oδ1-Asp298	O2-TPQ_{sq}	6.43
	Oδ2-Asp298	Oη-Tyr384	2.99 (HB)
	Cβ-Ala381	O2-TPQ_{sq}	4.13
	Oη-Tyr284	O-Wat1	2.75 (HB)
	O2-TPQ_{sq}	O-Wat1	2.84 (HB)
	O4-TPQ_{sq}	Cu	2.33 (CD)
**TS (1, 6)**	Oδ1-Asp298	N5-TPQ_{sq}	5.39
	Oη-Tyr284	O4-TPQ_{sq}	2.48 (HB)
	Oη-Tyr284	N5-TPQ_{sq}	3.82
**6**	Oη-Tyr284	O4-TPQ_{sq}	2.52 (HB)
	Oη-Tyr284	N5-TPQ_{sq}	2.82 (HB)
	Oδ1-Asp298	N5-TPQ_{sq}	6.32
	Oδ2-Asp298	Oη-Tyr384	3.29 (HB)
	Oδ-Asn381	N5-TPQ_{sq}	3.26 (HB)
	Nδ-Asn381	N5-TPQ_{sq}	3.47
	O4-TPQ_{sq}	Cu	4.07
**TS (6, 5)**	Oδ1-Asp298	Oη-Tyr384	3.05 (HB)

	O2-TPQ382	Oη-Tyr384	2.57
	Cε1-His433	C3-Tyr384	3.12
	Oη-Tyr284	O4-TPQ_{sq}	3.70
	O4-TPQ_{sq}	Cu	2.62
**TS (6, 7)**	Oη-Tyr284	O4-TPQ_{sq}	3.26 (HB)
	Oη-Tyr284	N5-TPQ_{sq}	2.52 (HB)
	Oδ2-Asp298	Oη-Tyr384	3.32
	Nδ-Asn381	N5-TPQ_{sq}	2.88 (HB)
	O4-TPQ_{sq}	Cu	3.16
**7**	Oη-Tyr284	N5-TPQ_{sq}	3.63
	Oδ2-Asp298	O-Tyr384	2.94 (HB)
	Nδ-Asn381	N5-TPQ_{sq}	3.43
	O4-TPQ_{sq}	Cu	2.12 (CD)
**OX**	Oη-Tyr284	O4-TPQ_{ox}	2.55 (HB)
	O-Wat_{ax}	O2-TPQ_{ox}	2.89
	Oδ1-Asp298	O5-TPQ_{ox}	3.35
**TS (OX, OXrot)**	Oη-Tyr284	O4-TPQ_{ox}	2.50 (HB)
	Oδ-Asn381	O5-TPQ_{ox}	2.78
	Nδ-Asn381	O5-TPQ_{ox}	2.83 (HB)
	Oη-Tyr384	O2-TPQ_{ox}	2.51 (HB)
**OX_{rot}**	Oη-Tyr284	O4-TPQ_{ox}	2.55 (HB)
	O-Wat_{ax}	O5-TPQ_{ox}	2.63 (HB)
	Oδ1-Asp298	O2-TPQ_{ox}	3.43
**OX^{NA}**	Oη-Tyr284	O4-TPQ_{ox}	2.55 (HB)
	O-Wat_{ax}	O2-TPQ_{ox}	2.94 (HB)
	Oδ1-Asp298	O5-TPQ_{ox}	3.34
**TS (OX^{NA}, OX_{rot}^{NA})**	Oη-Tyr284	O4-TPQ_{ox}	2.52 (HB)
	Oη-Tyr384	O2-TPQ_{ox}	2.47 (HB)
	Cβ-Ala381	O5-TPQ_{ox}	3.77
**OX_{rot}^{NA}**	Oη-Tyr284	O4-TPQ_{ox}	2.53 (HB)
	O-Wat_{ax}	O5-TPQ_{ox}	2.62 (HB)
	Oδ1-Asp298	O2-TPQ_{ox}	3.43

[a]A distance is attributed to a hydrogen bond whenever it is shorter than 3.3 Å and labeled as HB.
[b]Coordination bonds are indicated as CD.

Table S3. Comparison of the QM region for the models used in our simulations with respect to the corresponding region in the X-ray crystal structures of the catalytic intermediates during the reductive half-reaction. Root-Mean-Square deviation (RMSD) values are reported for the atoms in the QM subsystem.[a]

	RMSD (Å)		
	WT, TPQ_{amr} (PDB ID: 3X3Z)	N381A holo/PEA (PDB ID: 7WIS)	WT, TPQ_{sq} (PDB ID: 3X3X)
**1h**	0.291	1.070	1.701
**3h**	1.108	0.376	1.879
**1**	0.317	1.069	1.683
**3**	1.104	0.364	1.897
**5**	1.803	1.947	0.516
$\mathbf{1h^{NA}}$	0.287	1.076	1.706
$\mathbf{3h^{NA}}$	1.110	0.392	1.869
$\mathbf{5^{NA}}$	1.817	1.968	0.567

[a]Fifty heavy atoms (non-hydrogen atoms) excluding the water oxygen atoms and carboxamide side chain of Asn381 (CγNδCδO) are used for the RMSD calculation.

Table S4. Comparison of the QM region for the models used in our simulations with respect to the corresponding region in the X-ray crystal structures of the oxidized form. Root-Mean-Square deviation (RMSD) values are reported for the atoms in the QM region.[a]

	RMSD (Å)		
	WT, Neutron structure (PDB ID: 6L9C)	N381A $_{holo}$/Conf. a (PDB ID: 7WIR)	N381A $_{holo}$/Conf. b (PDB ID: 7WIR)
**OX**	0.327	0.574	1.295
**OX_{rot}**	1.244	1.334	0.614
**OX^{NA}**	0.332	0.577	1.297
**OX_{rot}^{NA}**	1.239	1.328	0.610

[a]Fifty heavy atoms (non-hydrogen atoms) excluding the water oxygen atoms and carboxamide side chain of Asn381 (CγNδCδO) are used for the RMSD calculation.

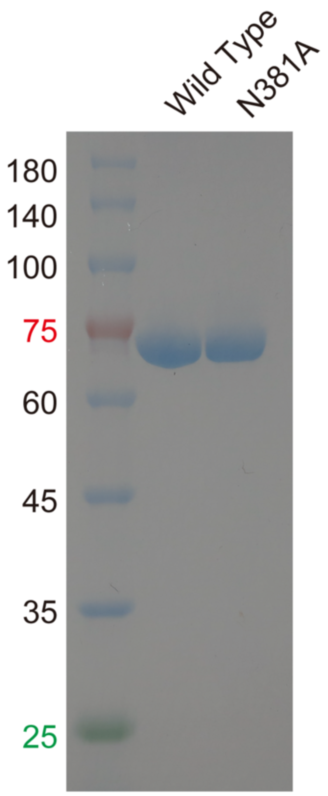

Figure S1. SDS-PAGE analysis of AGAO. Lane 1: molecular weight markers (kDa); Lanes 2 and 3: purified proteins (5 μg each) of wild-type (lane 2) and N381A mutant (lane 3) of AGAO.

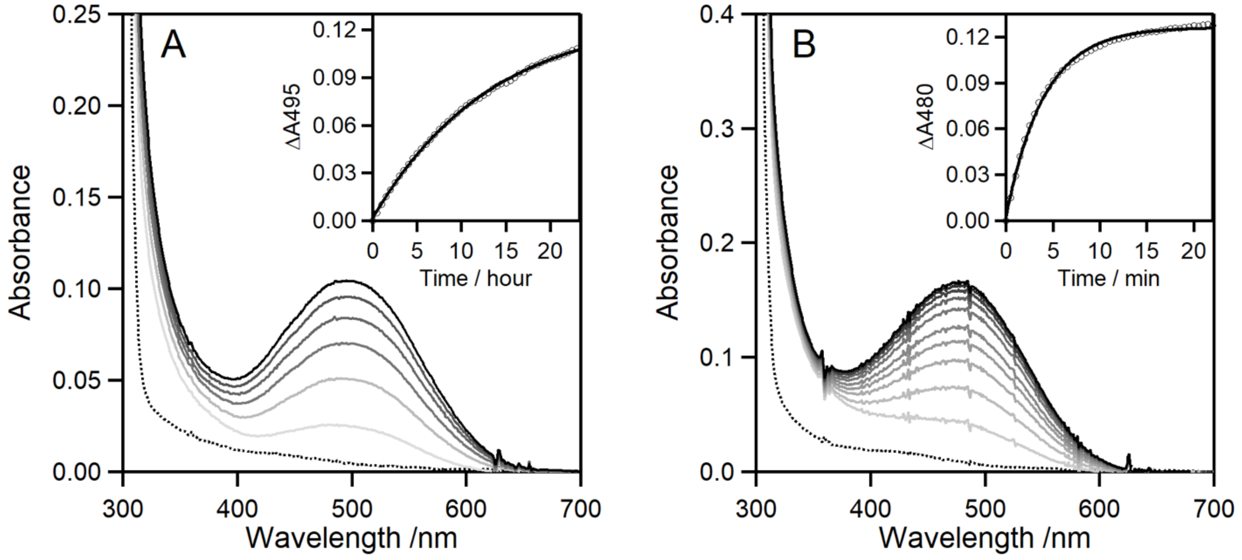

Figure S2. Spectral changes during the TPQ biogenesis of (A) the N381A mutant and (B) the WT AGAO. The UV/vis absorption spectra were recorded after mixing the enzyme (0.1 mM subunit) with 0.25 mM Cu^{2+} ion in 50 mM HEPES buffer, at a pH of 6.8 and a temperature of 30 ºC under aerobic conditions. The shown spectra are the ones obtained at (A) 4, 8, 12, 16, 20, and 24 h, and (B) 1, 2, 3, 4, 5, 7, 9, 11, and 15 min. Darker curves indicate later times. The dashed line represents the initial spectrum of the enzyme (apo form). Insets: open-circle marks indicate the absorbance changes at 495 (A) and 480 (B) nm as a function of time. The theoretical line is obtained by single-exponential kinetics.

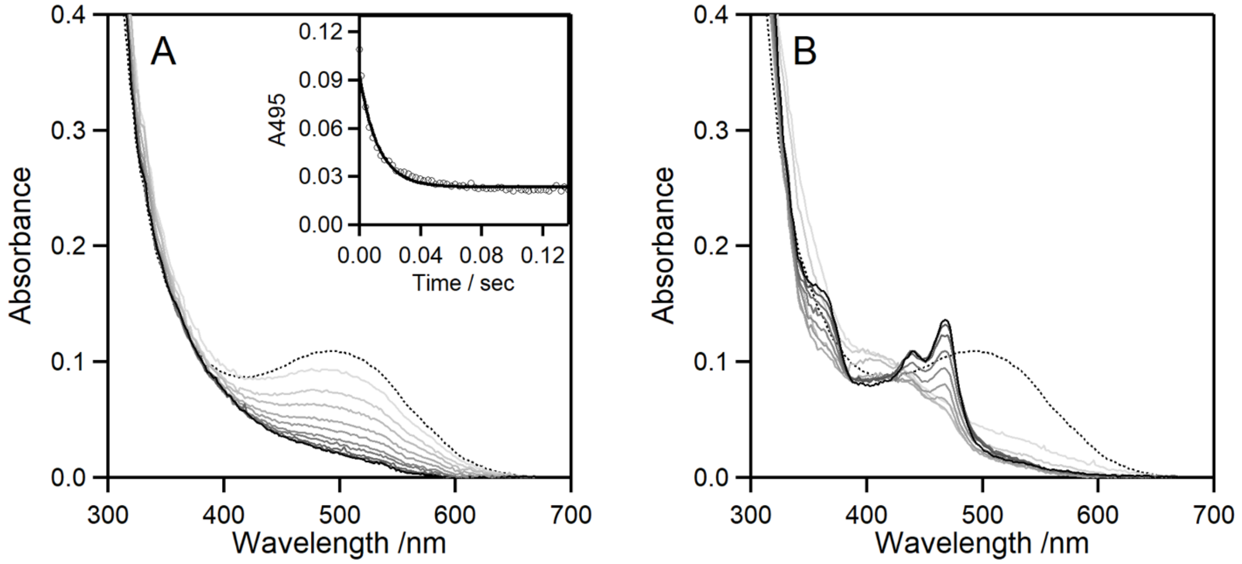

Figure S3. Transient spectral changes during the reductive half-reaction of (A) the N381A mutant and (B) the WT AGAO with 2-PEA. The UV/vis absorption spectra were recorded after mixing the enzyme (0.09 mM subunit) with 4 mM substrate in 50 mM HEPES buffer, at a pH of 6.8 and a temperature of 15 ºC under anaerobic conditions. The shown spectra are obtained at 2.3, 3.8, 6.4, 9.0, 12, 17, 24, 37, 63, 127 and 255 ms. As in the former figure, darker curves indicate later times. Dashed line represents the initial spectrum of TPQ_{ox}. The inset in panel (A) shows the absorbance changes at 495 nm in the N381A mutant, highlighted by open circles. The theoretical line is obtained by single-exponential kinetics.

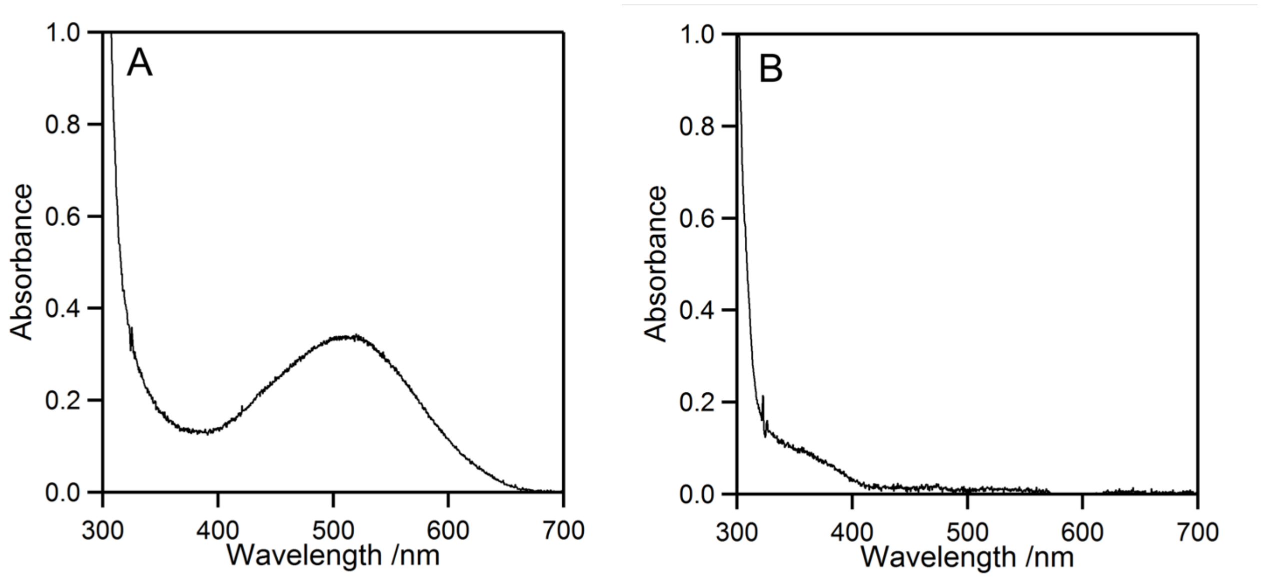

Figure S4. UV/vis absorption spectra of the N381A crystals. (A) N381A$_{holo}$ and (B) N381A$_{holo/PEA}$.

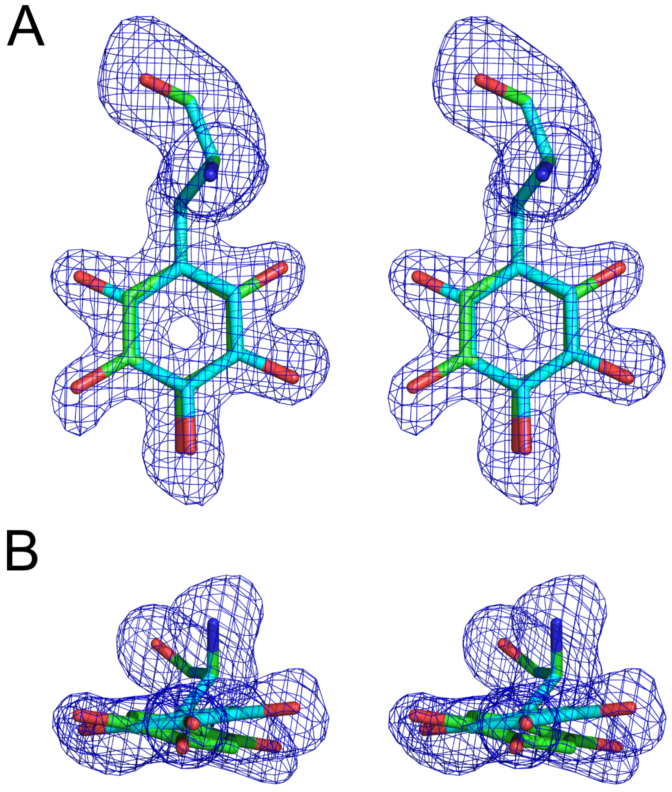

Figure S5. Structure of the cofactor of the N381A mutant of AGAO. (A) Stereo diagram of the refined model of TPQ_{ox}. (B) Identical representation of (A) seen along the direction parallel to the TPQ ring. The normal and flipped conformations of TPQ are shown in green and cyan, respectively. An F_o-F_c omit map, calculated without the contribution of TPQ, is shown as a blue mesh at 3.0 σ.

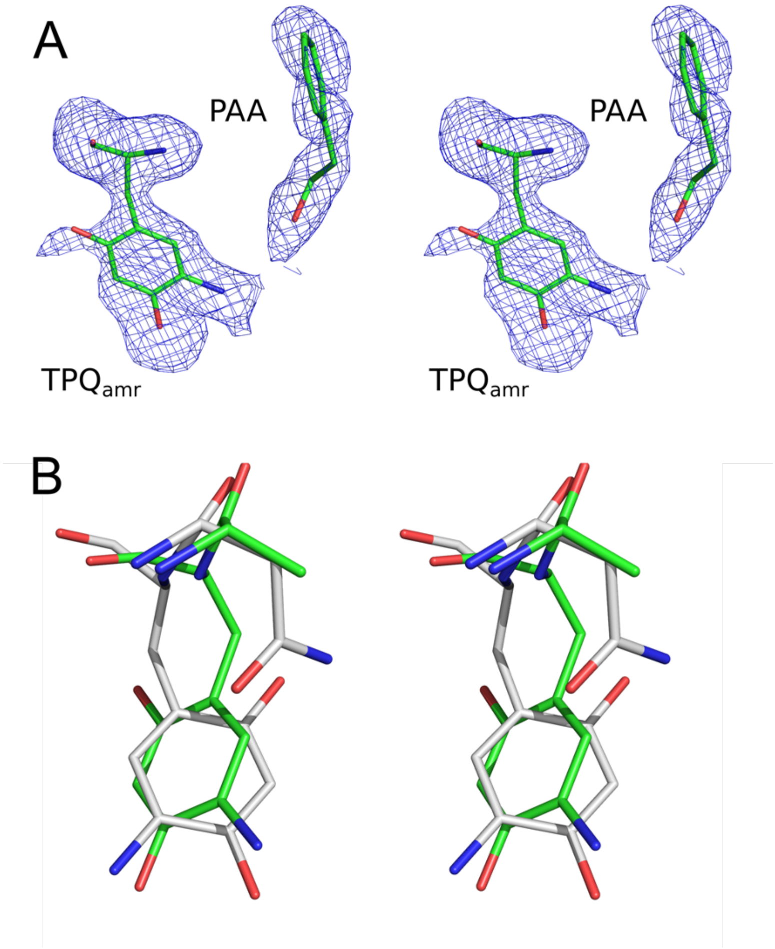

Figure S6. Structure of the active site of the TPQ_{amr} intermediate of the N381A mutant formed by reaction with 2-PEA. (A) Refined model of TPQ_{amr} and phenylacetaldehyde (PAA). An annealed F_o - F_c omit map, calculated without contributions of TPQ and PAA, is shown as a blue mesh at 2.3 σ. (B) The refined model of TPQ_{amr} of the N381A mutant (green) was superimposed to that of the WT AGAO (white) [S4].

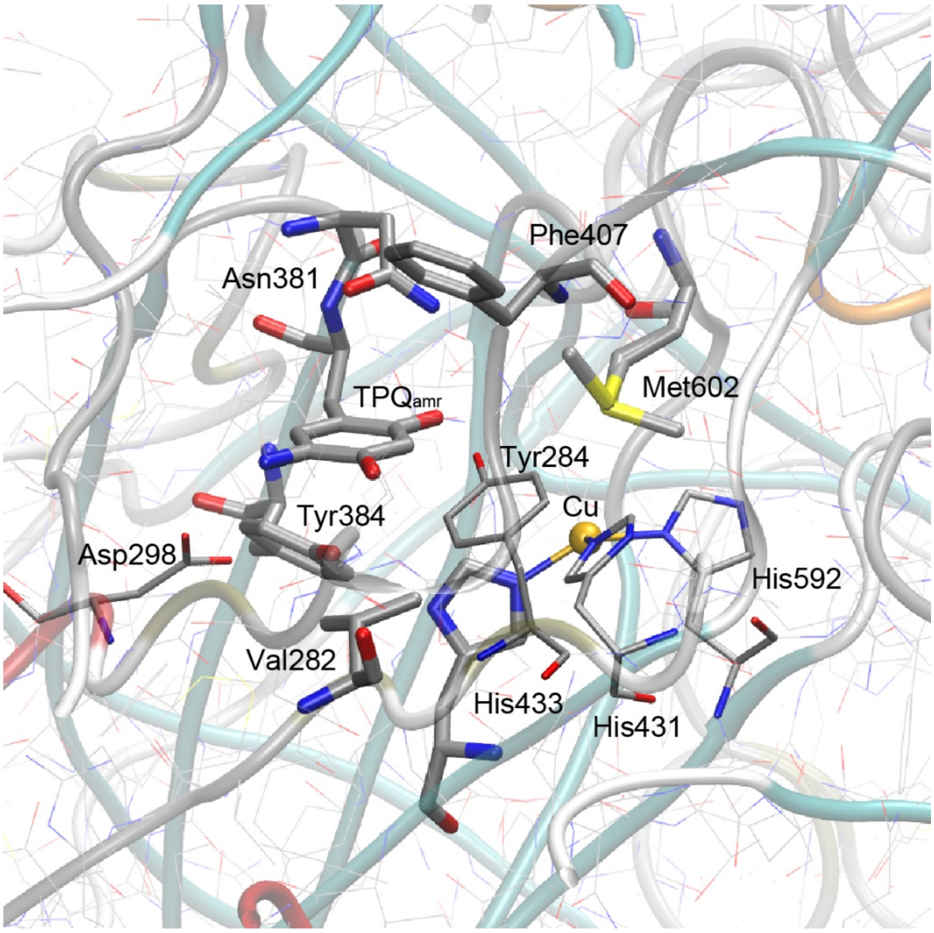

Figure S7. Amino acid residues surrounding the TPQ_{amr} (TPQ382) displayed in a tube representation. Amino acid residues highlighted as thicker tubes are the ones preventing the conformational change of the TPQ ring (see text for details). The molecular structure is taken from the crystal structure of AGAO (PDBID:3x3z) [S4]. An inhibitor Cl^- anion coordinated to the Cu cation is omitted.

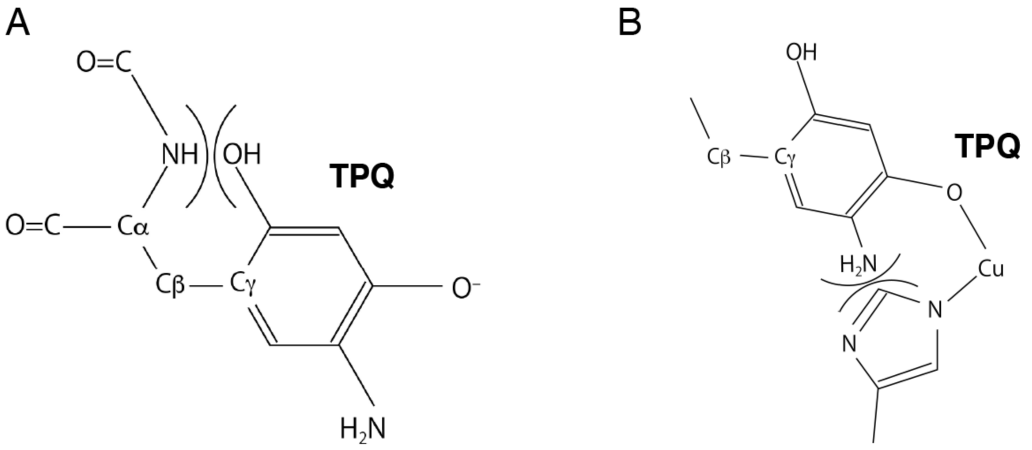

Figure S8. Steric hindrance acting during the TPQ conformational changes. (A) Counterclockwise-ring rotation of TPQ in pathway (II); (B) counterclockwise-ring rotation of TPQ in pathway (IV).

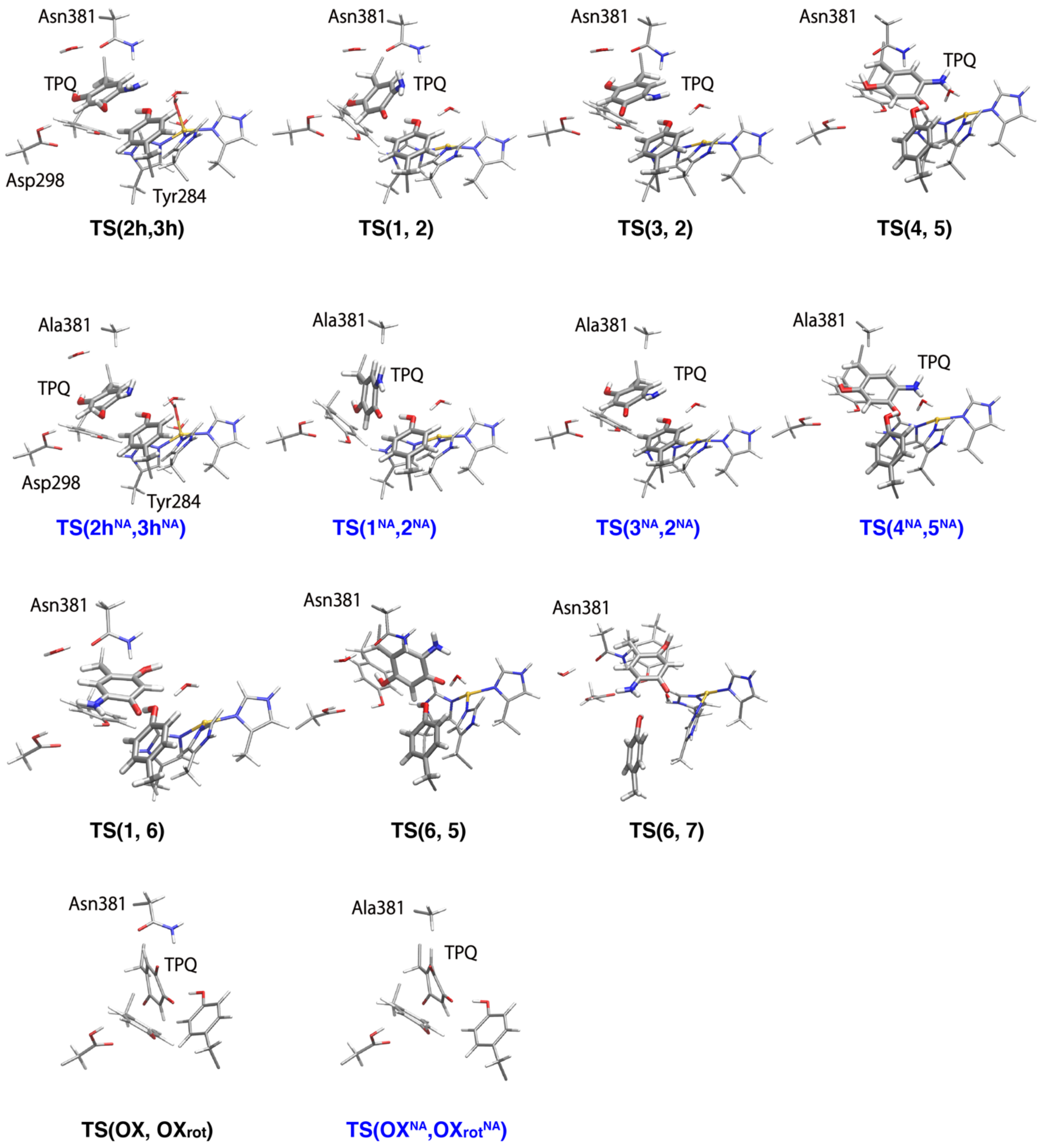

Figure S9. Molecular structures of the transition states. Details are discussed in the main text.

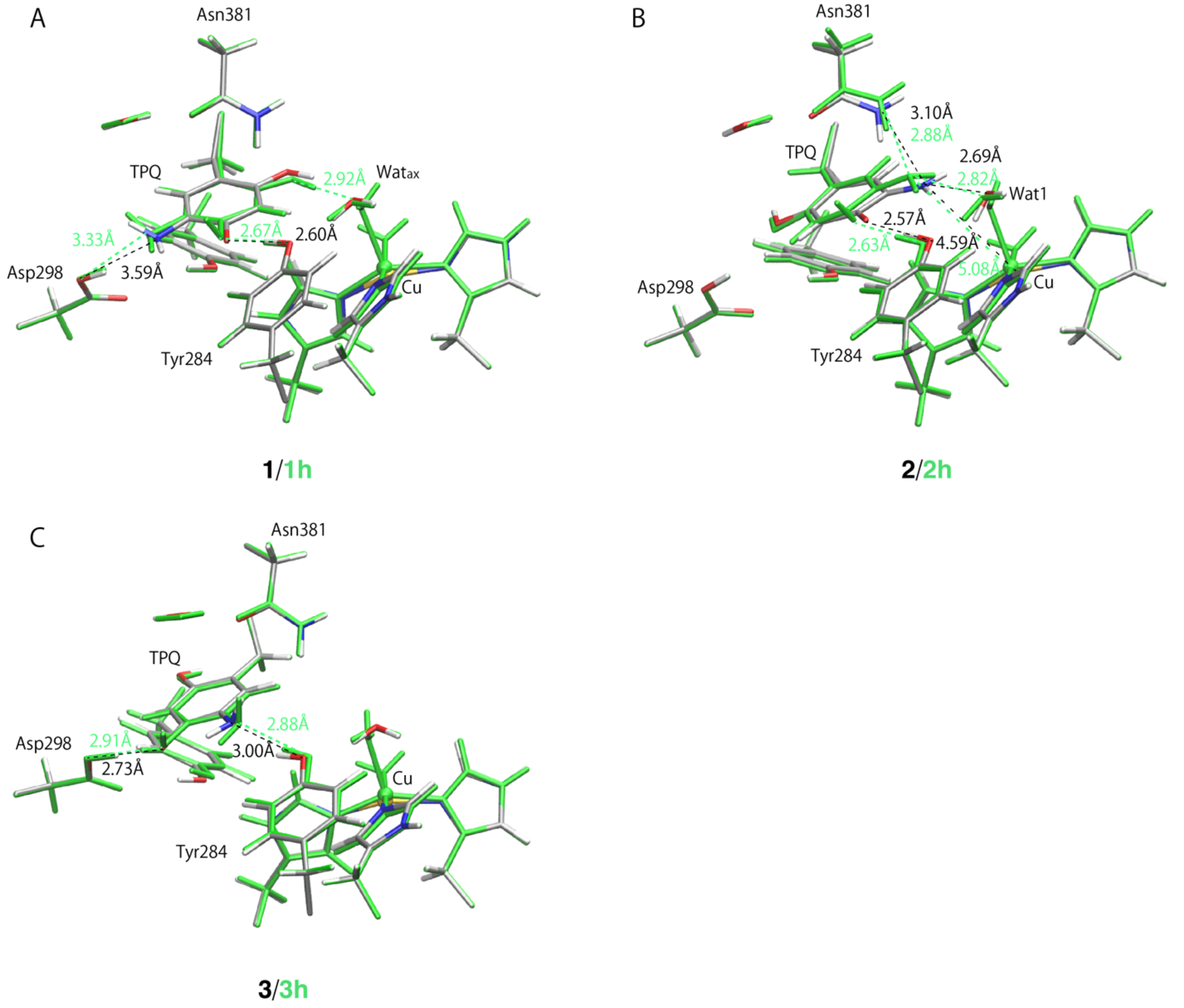

Figure S10. Superimposed structures realized upon the deprotonation of O4H-TPQ. (A) **1**/**1h**, (B) **2**/**2h** and (C) **3**/**3h**. Protonated states of TPQ_{amr} (**1h**, **2h**, **3h**) are colored in green. The corresponding deprotonated states of TPQ_{sq} (**1**, **2**, **3**) are colored according to the standard colors of the chemical elements (H=white, C=gray, N=blue, O=red). The Cu metal atom is highlighted as a green sphere.

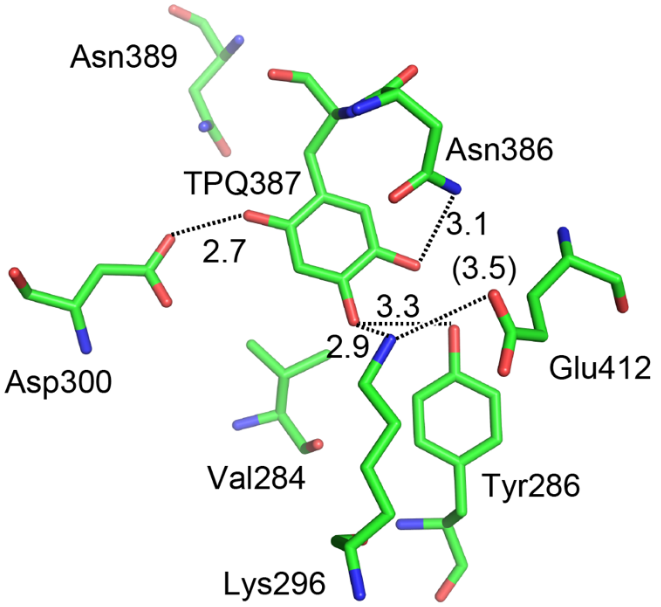

Figure S11. Active-site structure of the copper amine oxidase in *Pisum savitum* (PDB ID:1KSI) [S5]. The active-site residues including TPQ_{ox} are the ones corresponding to the X-ray crystal structure. Dotted lines indicate the hydrogen bonds. The numerical values in the figure show the length of hydrogen bonds in Å. The short O-N distance (3.5 Å) although not representing a hydrogen bond, is reported in parentheses as a complementary piece of information.

References

[S1] Matsuzaki, R.; Fukui, T.; Sato, H.; Ozaki, Y.; Tanizawa K. FEBS Lett 1994, 351, 360–364.

[S2] Kishishita, S.; Okajima T.; Kim, M.; Yamaguchi, H.; Hirota, S.; Suzuki, S.; Kuroda, S.; Tanizawa, K.; Mure M. J. Am. Chem. Soc. 2003, 125, 1041–1055.

[S3] Chiu, Y. C.; Okajima, T.; Murakawa, T.; Uchida, M.; Taki, M.; Hirota, S.; Kim, M.; Yamaguchi, H.; Kawano, Y.; Kamiya, N.; Kuroda, S.; Hayashi, H.; Yamamoto, Y.; Tanizawa, K. Biochemistry 2006, 45, 4105–4120.

[S4] Murakawa, T.; Hamaguchi, A.; Nakanishi, S.; Kataoka, M.; Nakai, T.; Kawano, Y.; Yamaguchi, H.; Hayashi, H.; Tanizawa, K.; Okajima, T. J. Biol. Chem. 2015, 290, 23094–23109.

[S5] Kumar, V;, Dooley, D. M.; Freeman, H. C.; Guss, J. M.; Harvey, I.; McGuirl, M. A.; Wilce, M. C.; Zubak, V. M. Structure 1996, 4, 943–955.

117

1h

C	11.985	-0.662	-21.880
H	12.207	-1.680	-22.204
H	12.439	0.020	-22.599
C	10.483	-0.528	-21.797
C	9.814	0.695	-21.666
H	10.373	1.627	-21.702
C	8.438	0.731	-21.447
H	7.927	1.680	-21.324
C	7.706	-0.460	-21.364
O	6.353	-0.448	-21.104
H	6.018	0.475	-21.209
C	8.344	-1.686	-21.533
H	7.765	-2.605	-21.490
C	9.721	-1.698	-21.740
H	10.258	-2.633	-21.828
C	4.717	9.922	-19.645
H	3.673	10.234	-19.754
H	5.008	10.042	-18.602
C	4.761	8.413	-19.942
O	4.504	7.589	-19.083
O	4.993	8.044	-21.209
H	4.926	7.060	-21.234
C	0.022	0.604	-24.448
H	0.175	0.463	-25.516
H	-0.403	-0.318	-24.049
C	1.374	0.947	-23.816
O	2.113	1.846	-24.245
N	1.727	0.206	-22.749
H	1.051	-0.426	-22.321
H	2.518	0.522	-22.202
C	0.406	3.640	-19.869
H	0.223	3.394	-18.818
H	0.401	4.730	-19.935
C	1.758	3.159	-20.272
C	2.311	1.923	-19.880
C	2.562	4.049	-20.967
H	2.162	5.028	-21.211
C	3.582	1.569	-20.339
H	4.000	0.591	-20.114
C	3.872	3.769	-21.342
C	4.326	2.474	-21.098
O	1.600	1.098	-19.050
H	2.124	0.287	-18.893
N	4.656	4.803	-21.894
H	5.599	4.508	-22.146
H	4.232	5.198	-22.733
O	5.547	2.041	-21.625
H	5.456	2.031	-22.594
C	-0.764	7.618	-15.635
H	-0.454	7.908	-14.622
H	-0.893	8.541	-16.202
C	0.306	6.783	-16.287
C	0.912	5.707	-15.627
H	0.569	5.431	-14.632
C	1.964	5.005	-16.209
H	2.446	4.183	-15.687
C	2.428	5.393	-17.466
O	3.512	4.727	-18.001
H	3.835	5.260	-18.750
C	1.813	6.432	-18.161
H	2.193	6.726	-19.133
C	0.761	7.117	-17.567
H	0.296	7.942	-18.100
C	9.470	-1.139	-18.228
H	9.772	-1.548	-19.194
H	9.570	-0.054	-18.308
C	8.051	-1.485	-17.927
N	7.482	-2.724	-18.191
H	7.932	-3.495	-18.686
C	6.229	-2.738	-17.697
H	5.556	-3.575	-17.762
N	5.954	-1.565	-17.137
C	7.077	-0.772	-17.279
H	7.117	0.236	-16.898
C	6.052	3.707	-14.061
H	5.565	4.677	-14.213
H	5.772	3.368	-13.060
C	5.537	2.708	-15.056
N	4.949	3.037	-16.266
H	4.783	3.959	-16.679
C	4.474	1.918	-16.849
H	3.959	1.917	-17.796
N	4.727	0.865	-16.076
C	5.387	1.346	-14.956
H	5.688	0.690	-14.152
C	6.307	-2.793	-14.036
H	6.842	-2.466	-14.931
H	6.040	-1.889	-13.471
C	5.024	-3.499	-14.417
N	4.109	-2.923	-15.306
C	3.068	-3.753	-15.386
H	2.187	-3.606	-15.992
N	3.254	-4.824	-14.600
H	2.614	-5.631	-14.519
C	4.477	-4.684	-13.979
H	4.827	-5.419	-13.269
CU	4.240	-1.092	-16.239
O	4.529	-2.235	-20.175
H	5.221	-1.600	-20.488
H	4.983	-3.121	-20.213
O	3.365	3.203	-26.299
H	3.138	4.138	-26.133
H	2.805	2.698	-25.675
O	3.074	-1.277	-18.214
H	3.670	-1.675	-18.926
H	2.287	-1.843	-18.177
O	2.342	-0.525	-15.533
H	1.942	0.173	-16.083
H	1.634	-1.166	-15.339
C	12.639	-0.420	-20.513
C	5.612	10.835	-20.532
C	-0.999	1.752	-24.264
C	-0.776	3.097	-20.692
C	-2.130	6.922	-15.555
C	10.449	-1.674	-17.150
C	7.588	3.912	-14.087
C	7.265	-3.615	-13.175

117

TS(1h,2h)

C	12.003	-0.664	-21.891
H	12.225	-1.684	-22.206
H	12.474	0.013	-22.606
C	10.501	-0.529	-21.833
C	9.835	0.687	-21.652
H	10.389	1.622	-21.651
C	8.460	0.714	-21.429
H	7.963	1.661	-21.266
C	7.725	-0.477	-21.386
O	6.372	-0.475	-21.097
H	6.097	0.463	-20.983
C	8.356	-1.693	-21.635
H	7.777	-2.613	-21.637
C	9.732	-1.696	-21.855
H	10.264	-2.622	-22.024
C	4.731	9.932	-19.638
H	3.681	10.202	-19.787
H	4.996	10.091	-18.594
C	4.870	8.438	-19.915
O	4.829	7.591	-19.044
O	4.952	8.099	-21.209
H	4.959	7.123	-21.247
C	-0.332	0.444	-24.564
H	-0.431	0.230	-25.615
H	-0.820	-0.364	-24.023
C	1.142	0.508	-24.308
O	1.862	1.428	-24.749
N	1.624	-0.544	-23.635
H	0.961	-1.122	-23.122
H	2.559	-0.432	-23.240
C	0.567	3.873	-20.181
H	0.412	4.009	-19.102
H	0.641	4.879	-20.620
C	1.871	3.167	-20.458
C	2.975	3.814	-19.897
C	2.146	2.123	-21.348
H	1.375	1.525	-21.816
C	4.276	3.451	-20.178
H	5.079	4.078	-19.875

C	3.453	1.821	-21.724
C	4.504	2.456	-21.088
O	2.839	4.923	-19.142
H	1.940	5.008	-18.791
N	3.737	1.022	-22.833
H	4.655	0.599	-22.867
H	3.410	1.393	-23.727
O	5.798	2.125	-21.435
H	5.795	2.016	-22.402
C	-0.858	7.778	-15.558
H	-0.700	8.212	-14.560
H	-1.015	8.608	-16.246
C	0.354	7.023	-15.955
C	1.005	6.156	-15.074
H	0.557	5.943	-14.106
C	2.244	5.618	-15.400
H	2.798	4.986	-14.712
C	2.829	5.986	-16.605
O	4.096	5.546	-16.870
H	4.437	6.115	-17.586
C	2.166	6.809	-17.521
H	2.650	7.096	-18.443
C	0.921	7.308	-17.193
H	0.403	7.974	-17.881
C	9.473	-1.140	-18.236
H	9.778	-1.548	-19.202
H	9.572	-0.055	-18.314
C	8.055	-1.486	-17.935
N	7.492	-2.730	-18.181
H	7.942	-3.504	-18.673
C	6.246	-2.747	-17.672
H	5.572	-3.585	-17.723
N	5.971	-1.572	-17.119
C	7.086	-0.772	-17.282
H	7.122	0.239	-16.911
C	6.048	3.712	-14.074
H	5.569	4.680	-14.257
H	5.756	3.393	-13.070
C	5.538	2.697	-15.058
N	4.950	3.018	-16.269
H	4.754	3.974	-16.609
C	4.484	1.893	-16.845
H	3.986	1.870	-17.805
N	4.741	0.845	-16.064
C	5.398	1.335	-14.948
H	5.704	0.684	-14.143
C	6.304	-2.799	-14.024
H	6.833	-2.468	-14.921
H	6.032	-1.899	-13.457
C	5.030	-3.519	-14.402
N	4.131	-2.953	-15.306
C	3.090	-3.778	-15.399
H	2.224	-3.634	-16.026
N	3.262	-4.842	-14.598
H	2.620	-5.647	-14.519
C	4.477	-4.700	-13.961
H	4.818	-5.431	-13.243
CU	4.267	-1.114	-16.193
O	4.483	-2.297	-20.090
H	5.179	-1.648	-20.336
H	4.949	-3.176	-20.164
O	3.367	3.118	-26.344
H	3.114	4.017	-26.067
H	2.730	2.527	-25.898
O	3.081	-1.391	-18.012
H	3.586	-1.770	-18.786
H	2.155	-1.655	-18.108
O	2.471	-0.557	-15.253
H	2.061	0.197	-15.709
H	1.771	-1.225	-15.140
C	12.639	-0.421	-20.515
C	5.616	10.843	-20.531
C	-1.068	1.762	-24.280
C	-0.687	3.209	-20.726
C	-2.127	6.932	-15.548
C	10.445	-1.674	-17.152
C	7.586	3.912	-14.087
C	7.267	-3.617	-13.171

117

2h

C	11.988	-0.663	-21.881
H	12.234	-1.671	-22.216
H	12.417	0.040	-22.594
C	10.485	-0.581	-21.779
C	9.773	0.622	-21.690
H	10.295	1.570	-21.786
C	8.406	0.613	-21.419
H	7.854	1.543	-21.325
C	7.733	-0.602	-21.242
O	6.400	-0.619	-20.890
H	6.004	0.215	-21.242
C	8.410	-1.809	-21.383
H	7.870	-2.744	-21.262
C	9.778	-1.779	-21.638
H	10.356	-2.694	-21.671
C	4.734	9.935	-19.632
H	3.686	10.231	-19.749
H	5.025	10.072	-18.592
C	4.806	8.434	-19.924
O	4.659	7.598	-19.052
O	4.917	8.086	-21.213
H	4.754	7.121	-21.259
C	-0.200	0.524	-24.536
H	-0.158	0.352	-25.605
H	-0.679	-0.345	-24.086
C	1.243	0.690	-24.068
O	1.957	1.679	-24.306
N	1.716	-0.397	-23.435
H	1.028	-1.024	-23.023
H	2.548	-0.294	-22.860
C	0.642	4.034	-20.240
H	0.560	4.179	-19.156
H	0.607	5.037	-20.683
C	1.953	3.362	-20.602
C	3.075	4.102	-21.028
C	2.091	1.973	-20.552
H	1.259	1.362	-20.208
C	4.201	3.476	-21.547
H	5.029	4.063	-21.930
C	3.231	1.319	-21.016
C	4.247	2.094	-21.577
O	3.159	5.476	-20.926
H	2.317	5.810	-20.584
N	3.282	-0.086	-21.043
H	4.198	-0.473	-21.203
H	2.854	-0.515	-20.233
O	5.342	1.435	-22.142
H	5.283	1.471	-23.113
C	-0.755	7.690	-15.639
H	-0.461	8.003	-14.628
H	-0.921	8.599	-16.218
C	0.342	6.891	-16.281
C	0.890	5.753	-15.676
H	0.492	5.412	-14.723
C	1.942	5.060	-16.267
H	2.371	4.179	-15.796
C	2.474	5.523	-17.470
O	3.531	4.835	-18.021
H	3.866	5.365	-18.770
C	1.937	6.648	-18.094
H	2.402	7.050	-18.984
C	0.874	7.315	-17.499
H	0.466	8.200	-17.982
C	9.477	-1.134	-18.222
H	9.781	-1.537	-19.187
H	9.575	-0.048	-18.296
C	8.061	-1.482	-17.918
N	7.502	-2.731	-18.155
H	7.952	-3.503	-18.650
C	6.257	-2.751	-17.644
H	5.588	-3.591	-17.692
N	5.975	-1.570	-17.106
C	7.088	-0.767	-17.274
H	7.120	0.248	-16.915
C	6.048	3.724	-14.082
H	5.574	4.698	-14.246
H	5.752	3.394	-13.083
C	5.533	2.723	-15.079
N	4.942	3.037	-16.293
H	4.782	3.957	-16.715

C	4.474	1.908	-16.866
H	3.963	1.887	-17.819
N	4.731	0.865	-16.078
C	5.389	1.362	-14.966
H	5.696	0.716	-14.157
C	6.304	-2.799	-14.023
H	6.833	-2.465	-14.918
H	6.029	-1.902	-13.452
C	5.032	-3.522	-14.402
N	4.137	-2.960	-15.313
C	3.096	-3.787	-15.406
H	2.231	-3.647	-16.036
N	3.266	-4.846	-14.600
H	2.624	-5.651	-14.521
C	4.478	-4.701	-13.958
H	4.817	-5.428	-13.234
CU	4.282	-1.108	-16.169
O	4.486	-2.399	-19.958
H	5.225	-1.762	-20.098
H	4.936	-3.282	-20.123
O	3.332	3.157	-26.268
H	3.115	4.085	-26.060
H	2.781	2.634	-25.650
O	3.035	-1.379	-17.999
H	3.554	-1.893	-18.681
H	2.120	-1.699	-18.019
O	2.537	-0.571	-15.149
H	2.113	0.217	-15.529
H	1.837	-1.237	-15.027
C	12.640	-0.420	-20.513
C	5.615	10.840	-20.532
C	-1.071	1.768	-24.287
C	-0.617	3.278	-20.717
C	-2.088	6.938	-15.559
C	10.452	-1.674	-17.147
C	7.587	3.914	-14.089
C	7.268	-3.617	-13.172

117

TS(2h,3h)

C	11.983	-0.656	-21.884
H	12.202	-1.673	-22.211
H	12.437	0.029	-22.601
C	10.481	-0.520	-21.802
C	9.811	0.706	-21.700
H	10.366	1.638	-21.765
C	8.438	0.746	-21.456
H	7.913	1.691	-21.352
C	7.731	-0.449	-21.307
O	6.405	-0.464	-20.957
H	6.016	0.432	-20.928
C	8.358	-1.679	-21.473
H	7.779	-2.592	-21.369
C	9.727	-1.695	-21.709
H	10.264	-2.633	-21.761
C	4.729	9.931	-19.640
H	3.681	10.219	-19.770
H	5.009	10.071	-18.597
C	4.826	8.434	-19.931
O	4.705	7.584	-19.071
O	4.951	8.100	-21.225
H	4.880	7.129	-21.266
C	-0.122	0.532	-24.532
H	-0.043	0.378	-25.602
H	-0.583	-0.358	-24.105
C	1.294	0.752	-24.016
O	2.006	1.724	-24.303
N	1.753	-0.262	-23.261
H	1.083	-0.907	-22.847
H	2.635	-0.113	-22.791
C	0.366	3.348	-19.733
H	0.252	2.708	-18.850
H	0.240	4.355	-19.318
C	1.713	3.179	-20.322
C	2.492	4.296	-20.628
C	2.209	1.910	-20.574
H	1.578	1.039	-20.418
C	3.790	4.177	-21.098
H	4.385	5.054	-21.325
C	3.487	1.773	-21.074
C	4.282	2.902	-21.254
O	2.074	5.552	-20.376
H	1.136	5.544	-20.137
N	4.056	0.557	-21.419
H	4.656	0.548	-22.230
H	3.612	-0.309	-21.162
O	5.569	2.661	-21.680
H	5.518	2.872	-22.615
C	-0.778	7.668	-15.605
H	-0.510	8.005	-14.594
H	-0.929	8.568	-16.203
C	0.340	6.866	-16.194
C	0.852	5.718	-15.581
H	0.408	5.366	-14.653
C	1.921	5.021	-16.140
H	2.313	4.121	-15.674
C	2.506	5.508	-17.305
O	3.560	4.807	-17.852
H	3.867	5.312	-18.624
C	2.036	6.671	-17.909
H	2.530	7.059	-18.784
C	0.939	7.320	-17.369
H	0.562	8.217	-17.854
C	9.478	-1.137	-18.229
H	9.787	-1.539	-19.195
H	9.573	-0.051	-18.302
C	8.062	-1.491	-17.932
N	7.503	-2.735	-18.190
H	7.956	-3.503	-18.687
C	6.257	-2.761	-17.681
H	5.587	-3.600	-17.744
N	5.977	-1.590	-17.121
C	7.089	-0.786	-17.276
H	7.121	0.224	-16.901
C	6.051	3.711	-14.066
H	5.568	4.678	-14.246
H	5.762	3.397	-13.060
C	5.543	2.691	-15.043
N	4.954	2.994	-16.259
H	4.773	3.913	-16.678
C	4.493	1.860	-16.825
H	3.983	1.826	-17.778
N	4.754	0.824	-16.031
C	5.407	1.329	-14.919
H	5.716	0.689	-14.106
C	6.303	-2.804	-14.026
H	6.831	-2.471	-14.922
H	6.025	-1.905	-13.458
C	5.034	-3.532	-14.405
N	4.143	-2.984	-15.327
C	3.102	-3.810	-15.413
H	2.239	-3.678	-16.047
N	3.268	-4.857	-14.591
H	2.626	-5.661	-14.507
C	4.477	-4.704	-13.946
H	4.813	-5.422	-13.212
CU	4.286	-1.136	-16.176
O	4.504	-2.278	-20.088
H	5.221	-1.628	-20.285
H	4.957	-3.162	-20.181
O	3.373	3.155	-26.289
H	3.155	4.083	-26.082
H	2.826	2.637	-25.667
O	3.125	-1.260	-18.043
H	3.623	-1.725	-18.770
H	2.183	-1.445	-18.162
O	2.527	-0.611	-15.173
H	2.136	0.210	-15.515
H	1.805	-1.261	-15.099
C	12.637	-0.420	-20.514
C	5.615	10.841	-20.532
C	-1.038	1.745	-24.289
C	-0.802	3.042	-20.684
C	-2.111	6.911	-15.540
C	10.449	-1.674	-17.149
C	7.588	3.912	-14.085
C	7.268	-3.618	-13.171

117

3h

C	11.909	-0.651	-21.861
H	12.088	-1.679	-22.186
H	12.365	0.007	-22.602
C	10.402	-0.440	-21.754
C	9.791	0.820	-21.662
H	10.397	1.721	-21.708
C	8.408	0.943	-21.482
H	7.963	1.930	-21.388
C	7.591	-0.189	-21.399
O	6.232	-0.127	-21.181
H	5.838	0.782	-21.295
C	8.177	-1.452	-21.518
H	7.550	-2.338	-21.468
C	9.558	-1.556	-21.689
H	10.016	-2.536	-21.745
C	4.726	9.924	-19.655
H	3.677	10.199	-19.804
H	4.986	10.069	-18.608
C	4.862	8.429	-19.946
O	4.763	7.587	-19.072
O	4.999	8.088	-21.233
H	5.053	7.103	-21.274
C	0.005	0.563	-24.495
H	0.146	0.432	-25.564
H	-0.431	-0.358	-24.108
C	1.388	0.847	-23.895
O	2.112	1.789	-24.246
N	1.805	-0.053	-22.981
H	1.156	-0.727	-22.581
H	2.710	0.080	-22.551
C	0.345	2.555	-19.786
H	0.529	1.474	-19.774
H	0.069	2.812	-18.756
C	1.608	3.251	-20.225
C	1.729	4.650	-20.168
C	2.687	2.517	-20.734
H	2.598	1.436	-20.788
C	2.900	5.265	-20.596
H	2.995	6.341	-20.563
C	3.858	3.113	-21.204
C	3.941	4.508	-21.104
O	0.755	5.481	-19.719
H	0.150	5.021	-19.116
N	4.887	2.326	-21.851
H	4.560	2.066	-22.786
H	5.726	2.891	-21.989
O	5.084	5.191	-21.473
H	5.312	4.912	-22.370
C	-0.797	7.657	-15.554
H	-0.578	8.014	-14.537
H	-0.911	8.544	-16.180
C	0.347	6.840	-16.065
C	0.797	5.668	-15.445
H	0.285	5.304	-14.559
C	1.890	4.966	-15.949
H	2.227	4.039	-15.492
C	2.561	5.476	-17.058
O	3.607	4.758	-17.600
H	3.877	5.218	-18.416
C	2.165	6.668	-17.649
H	2.727	7.070	-18.477
C	1.043	7.320	-17.172
H	0.710	8.232	-17.661
C	9.481	-1.142	-18.235
H	9.796	-1.541	-19.200
H	9.571	-0.055	-18.308
C	8.065	-1.504	-17.950
N	7.505	-2.741	-18.240
H	7.962	-3.503	-18.744
C	6.256	-2.778	-17.737
H	5.585	-3.615	-17.826
N	5.979	-1.622	-17.146
C	7.091	-0.815	-17.279
H	7.121	0.186	-16.880
C	6.056	3.693	-14.043
H	5.559	4.648	-14.245
H	5.779	3.402	-13.026
C	5.558	2.639	-14.987
N	4.978	2.924	-16.206
H	4.767	3.845	-16.613
C	4.523	1.784	-16.760
H	4.015	1.731	-17.712
N	4.787	0.758	-15.955
C	5.433	1.277	-14.846
H	5.746	0.645	-14.027
C	6.302	-2.811	-14.032
H	6.831	-2.479	-14.929
H	6.020	-1.910	-13.469
C	5.037	-3.549	-14.409
N	4.153	-3.023	-15.351
C	3.111	-3.848	-15.423
H	2.252	-3.728	-16.065
N	3.271	-4.875	-14.575
H	2.629	-5.677	-14.484
C	4.476	-4.709	-13.928
H	4.806	-5.413	-13.177
CU	4.294	-1.181	-16.190
O	4.554	-2.120	-20.237
H	5.206	-1.446	-20.546
H	5.035	-2.985	-20.246
O	3.430	3.150	-26.318
H	3.213	4.079	-26.116
H	2.885	2.640	-25.688
O	3.247	-1.077	-18.136
H	3.740	-1.494	-18.898
H	2.310	-1.052	-18.374
O	2.513	-0.676	-15.221
H	2.171	0.193	-15.489
H	1.757	-1.292	-15.213
C	12.627	-0.420	-20.518
C	5.615	10.842	-20.534
C	-0.996	1.725	-24.288
C	-0.879	2.813	-20.688
C	-2.139	6.918	-15.545
C	10.451	-1.674	-17.150
C	7.589	3.911	-14.084
C	7.269	-3.618	-13.172

110

1hNA

C	11.997	-0.660	-21.887
H	12.220	-1.679	-22.207
H	12.459	0.021	-22.602
C	10.495	-0.526	-21.812
C	9.829	0.696	-21.670
H	10.387	1.630	-21.708
C	8.456	0.730	-21.431
H	7.948	1.678	-21.298
C	7.726	-0.460	-21.337
O	6.381	-0.449	-21.034
H	6.059	0.483	-21.066
C	8.357	-1.686	-21.535
H	7.776	-2.604	-21.497
C	9.730	-1.696	-21.764
H	10.265	-2.629	-21.876
C	4.711	9.931	-19.645
H	3.668	10.246	-19.761
H	4.996	10.066	-18.603
C	4.758	8.419	-19.921
O	4.493	7.615	-19.042
O	4.997	8.023	-21.176
H	4.959	7.035	-21.166
C	0.006	0.597	-24.414
H	0.231	0.463	-25.469
H	-0.430	-0.320	-24.022
H	0.916	0.817	-23.843
C	0.434	3.755	-19.943
H	0.265	3.567	-18.877
H	0.426	4.841	-20.074
C	1.784	3.253	-20.326
C	2.265	1.972	-19.986
C	2.656	4.147	-20.931
H	2.311	5.157	-21.127
C	3.541	1.589	-20.402
H	3.895	0.576	-20.239
C	3.973	3.834	-21.247
C	4.364	2.510	-21.047
O	1.482	1.144	-19.236
H	1.963	0.310	-19.087
N	4.839	4.865	-21.692
H	5.796	4.536	-21.819

H	4.551	5.239	-22.597
O	5.625	2.072	-21.476
H	5.619	2.041	-22.447
C	-0.772	7.628	-15.628
H	-0.470	7.923	-14.614
H	-0.906	8.550	-16.196
C	0.304	6.800	-16.275
C	0.912	5.726	-15.614
H	0.564	5.443	-14.623
C	1.974	5.033	-16.190
H	2.461	4.216	-15.664
C	2.440	5.427	-17.443
O	3.531	4.773	-17.975
H	3.861	5.321	-18.711
C	1.821	6.462	-18.141
H	2.200	6.761	-19.110
C	0.763	7.141	-17.550
H	0.301	7.968	-18.084
C	9.471	-1.139	-18.232
H	9.776	-1.548	-19.197
H	9.572	-0.055	-18.311
C	8.051	-1.486	-17.936
N	7.484	-2.726	-18.200
H	7.936	-3.494	-18.699
C	6.230	-2.739	-17.715
H	5.554	-3.574	-17.788
N	5.951	-1.566	-17.157
C	7.075	-0.773	-17.294
H	7.111	0.235	-16.914
C	6.050	3.705	-14.066
H	5.565	4.675	-14.223
H	5.765	3.365	-13.068
C	5.539	2.708	-15.066
N	4.959	3.038	-16.280
H	4.796	3.962	-16.692
C	4.485	1.921	-16.867
H	3.976	1.918	-17.820
N	4.730	0.867	-16.093
C	5.384	1.345	-14.970
H	5.675	0.688	-14.165
C	6.310	-2.792	-14.040
H	6.846	-2.466	-14.935
H	6.041	-1.889	-13.475
C	5.027	-3.498	-14.424
N	4.118	-2.927	-15.322
C	3.077	-3.756	-15.404
H	2.201	-3.614	-16.019
N	3.257	-4.823	-14.609
H	2.615	-5.627	-14.529
C	4.476	-4.680	-13.984
H	4.823	-5.412	-13.268
CU	4.240	-1.095	-16.265
O	4.525	-2.241	-20.160
H	5.221	-1.608	-20.462
H	4.973	-3.129	-20.207
O	3.046	-1.238	-18.224
H	3.636	-1.650	-18.931
H	2.245	-1.787	-18.202
O	2.365	-0.545	-15.498
H	1.963	0.165	-16.029
H	1.665	-1.199	-15.324
C	12.640	-0.420	-20.514
C	5.611	10.835	-20.536
C	-1.002	1.757	-24.257
C	-0.755	3.163	-20.717
C	-2.133	6.923	-15.555
C	10.443	-1.675	-17.150
C	7.588	3.911	-14.085
C	7.265	-3.615	-13.177

113

TS(1hNA,2hNA)

C	11.997	-0.656	-21.888
H	12.229	-1.669	-22.217
H	12.451	0.035	-22.599
C	10.494	-0.544	-21.813
C	9.804	0.664	-21.661
H	10.342	1.608	-21.690
C	8.431	0.670	-21.420
H	7.913	1.610	-21.270
C	7.721	-0.534	-21.333
O	6.378	-0.558	-21.032
H	6.031	0.364	-21.070
C	8.378	-1.745	-21.543
H	7.819	-2.675	-21.498
C	9.751	-1.728	-21.775
H	10.306	-2.649	-21.893
C	4.728	9.937	-19.637
H	3.679	10.223	-19.771
H	5.005	10.082	-18.594
C	4.840	8.443	-19.924
O	4.748	7.597	-19.055
O	4.958	8.103	-21.215
H	4.931	7.128	-21.254
C	-0.009	0.592	-24.435
H	0.178	0.441	-25.496
H	-0.428	-0.320	-24.014
H	0.923	0.839	-23.921
C	0.499	3.828	-20.136
H	0.341	3.936	-19.055
H	0.570	4.849	-20.543
C	1.813	3.159	-20.446
C	2.955	3.867	-20.051
C	2.016	2.044	-21.251
H	1.207	1.409	-21.596
C	4.234	3.459	-20.385
H	5.079	4.052	-20.082
C	3.272	1.734	-21.763
C	4.385	2.384	-21.238
O	2.844	5.064	-19.425
H	2.016	5.109	-18.919
N	3.364	0.876	-22.830
H	2.571	0.327	-23.116
H	4.220	0.752	-23.341
O	5.667	1.947	-21.563
H	5.763	1.918	-22.525
C	-0.874	7.739	-15.547
H	-0.684	8.129	-14.537
H	-1.029	8.601	-16.196
C	0.309	6.981	-16.011
C	0.977	6.080	-15.179
H	0.553	5.835	-14.209
C	2.201	5.548	-15.559
H	2.766	4.879	-14.917
C	2.759	5.965	-16.762
O	4.011	5.529	-17.074
H	4.299	6.061	-17.841
C	2.069	6.814	-17.636
H	2.524	7.134	-18.562
C	0.832	7.298	-17.259
H	0.288	7.971	-17.918
C	9.473	-1.139	-18.232
H	9.779	-1.546	-19.197
H	9.572	-0.054	-18.309
C	8.054	-1.486	-17.935
N	7.484	-2.722	-18.209
H	7.932	-3.490	-18.710
C	6.232	-2.739	-17.713
H	5.551	-3.568	-17.791
N	5.961	-1.571	-17.142
C	7.084	-0.778	-17.279
H	7.124	0.227	-16.891
C	6.048	3.712	-14.066
H	5.567	4.682	-14.240
H	5.760	3.389	-13.063
C	5.532	2.704	-15.054
N	4.949	3.034	-16.266
H	4.766	3.987	-16.617
C	4.483	1.911	-16.850
H	3.992	1.894	-17.813
N	4.732	0.858	-16.073
C	5.384	1.342	-14.951
H	5.685	0.687	-14.147
C	6.302	-2.799	-14.026
H	6.831	-2.469	-14.924
H	6.031	-1.897	-13.459
C	5.027	-3.516	-14.407
N	4.141	-2.953	-15.326
C	3.098	-3.775	-15.429
H	2.250	-3.631	-16.079
N	3.257	-4.833	-14.617

H	2.612	-5.636	-14.540
C	4.464	-4.691	-13.965
H	4.793	-5.419	-13.237
CU	4.260	-1.115	-16.219
O	4.460	-2.292	-20.117
H	5.160	-1.654	-20.387
H	4.922	-3.172	-20.179
O	3.292	3.116	-26.287
H	3.051	4.029	-26.039
H	2.651	2.552	-25.830
O	3.051	-1.542	-17.998
H	3.573	-1.812	-18.807
H	2.173	-1.265	-18.295
O	2.471	-0.579	-15.260
H	2.061	0.203	-15.668
H	1.773	-1.258	-15.206
C	12.639	-0.420	-20.514
C	5.615	10.843	-20.531
C	-1.008	1.757	-24.261
C	-0.748	3.177	-20.738
C	-2.160	6.920	-15.543
C	10.445	-1.674	-17.150
C	7.586	3.912	-14.086
C	7.266	-3.617	-13.172

110

2hNA

C	11.974	-0.639	-21.886
H	12.222	-1.636	-22.243
H	12.405	0.077	-22.587
C	10.465	-0.561	-21.788
C	9.732	0.629	-21.670
H	10.239	1.588	-21.734
C	8.358	0.592	-21.413
H	7.789	1.510	-21.289
C	7.705	-0.639	-21.282
O	6.367	-0.688	-20.961
H	5.912	0.066	-21.406
C	8.402	-1.831	-21.451
H	7.865	-2.775	-21.369
C	9.771	-1.775	-21.696
H	10.361	-2.678	-21.776
C	4.731	9.919	-19.635
H	3.678	10.211	-19.759
H	5.019	10.046	-18.594
C	4.808	8.421	-19.934
O	4.671	7.580	-19.068
O	4.940	8.077	-21.222
H	4.839	7.105	-21.265
C	0.010	0.584	-24.414
H	0.224	0.430	-25.471
H	-0.431	-0.332	-24.021
H	0.929	0.803	-23.855
C	0.552	3.868	-20.130
H	0.455	3.872	-19.036
H	0.554	4.927	-20.428
C	1.842	3.204	-20.563
C	3.014	3.901	-20.927
C	1.870	1.811	-20.691
H	1.003	1.228	-20.383
C	4.114	3.221	-21.450
H	5.004	3.773	-21.742
C	2.903	1.105	-21.308
C	4.042	1.851	-21.672
O	3.181	5.267	-20.762
H	2.325	5.667	-20.548
N	2.699	-0.226	-21.646
H	3.409	-0.918	-21.491
H	1.743	-0.567	-21.693
O	5.130	1.197	-22.253
H	5.231	1.428	-23.188
C	-0.764	7.633	-15.626
H	-0.466	7.928	-14.610
H	-0.900	8.556	-16.191
C	0.321	6.811	-16.273
C	0.850	5.659	-15.675
H	0.450	5.324	-14.721
C	1.896	4.957	-16.266
H	2.314	4.069	-15.796
C	2.450	5.423	-17.461
O	3.505	4.721	-17.996
H	3.782	5.169	-18.819
C	1.943	6.574	-18.071
H	2.429	6.987	-18.948
C	0.878	7.245	-17.477
H	0.494	8.146	-17.951
C	9.470	-1.131	-18.230
H	9.775	-1.539	-19.196
H	9.566	-0.047	-18.303
C	8.052	-1.479	-17.930
N	7.482	-2.711	-18.218
H	7.935	-3.470	-18.729
C	6.234	-2.738	-17.712
H	5.552	-3.565	-17.803
N	5.959	-1.572	-17.139
C	7.080	-0.774	-17.273
H	7.119	0.228	-16.877
C	6.040	3.718	-14.071
H	5.565	4.691	-14.231
H	5.754	3.387	-13.068
C	5.517	2.717	-15.062
N	4.936	3.035	-16.282
H	4.765	3.954	-16.704
C	4.474	1.906	-16.862
H	3.978	1.892	-17.821
N	4.720	0.861	-16.074
C	5.365	1.354	-14.953
H	5.657	0.706	-14.140
C	6.291	-2.801	-14.026
H	6.817	-2.471	-14.926
H	6.012	-1.903	-13.458
C	5.020	-3.524	-14.409
N	4.136	-2.968	-15.335
C	3.090	-3.787	-15.438
H	2.247	-3.651	-16.097
N	3.243	-4.834	-14.613
H	2.601	-5.638	-14.530
C	4.446	-4.690	-13.958
H	4.769	-5.417	-13.228
CU	4.267	-1.117	-16.204
O	4.453	-2.312	-20.091
H	5.200	-1.685	-20.271
H	4.893	-3.203	-20.197
O	3.002	-1.585	-17.954
H	3.537	-1.843	-18.754
H	2.172	-1.192	-18.285
O	2.512	-0.577	-15.178
H	2.098	0.221	-15.548
H	1.815	-1.259	-15.138
C	12.637	-0.419	-20.512
C	5.615	10.838	-20.533
C	-1.002	1.755	-24.259
C	-0.725	3.213	-20.738
C	-2.132	6.924	-15.555
C	10.452	-1.674	-17.147
C	7.585	3.910	-14.089
C	7.266	-3.617	-13.170

113

TS(2hNA,3hNA)

C	11.965	-0.646	-21.879
H	12.180	-1.662	-22.215
H	12.413	0.042	-22.596
C	10.461	-0.509	-21.786
C	9.784	0.713	-21.665
H	10.339	1.647	-21.710
C	8.406	0.748	-21.434
H	7.881	1.693	-21.314
C	7.696	-0.448	-21.318
O	6.367	-0.496	-20.990
H	5.898	0.356	-21.038
C	8.334	-1.673	-21.496
H	7.751	-2.586	-21.423
C	9.705	-1.684	-21.720
H	10.240	-2.621	-21.800
C	4.725	9.927	-19.645
H	3.676	10.210	-19.785
H	4.996	10.068	-18.601
C	4.834	8.433	-19.936
O	4.719	7.577	-19.081

O	4.968	8.100	-21.231
H	4.966	7.127	-21.262
C	-0.003	0.577	-24.437
H	0.221	0.454	-25.495
H	-0.440	-0.347	-24.060
H	0.911	0.791	-23.869
C	0.264	3.043	-19.643
H	0.164	2.209	-18.942
H	0.125	3.926	-19.007
C	1.611	3.064	-20.252
C	2.249	4.285	-20.485
C	2.221	1.876	-20.627
H	1.670	0.942	-20.534
C	3.565	4.321	-20.928
H	4.087	5.261	-21.065
C	3.492	1.904	-21.168
C	4.180	3.113	-21.196
O	1.655	5.464	-20.212
H	0.750	5.330	-19.895
N	4.126	0.769	-21.705
H	4.824	1.062	-22.386
H	3.463	0.157	-22.174
O	5.497	3.039	-21.590
H	5.492	3.367	-22.493
C	-0.788	7.632	-15.579
H	-0.523	7.968	-14.566
H	-0.924	8.535	-16.177
C	0.331	6.824	-16.160
C	0.825	5.669	-15.547
H	0.364	5.316	-14.628
C	1.899	4.970	-16.092
H	2.276	4.061	-15.632
C	2.509	5.466	-17.241
O	3.558	4.754	-17.786
H	3.847	5.225	-18.586
C	2.065	6.641	-17.839
H	2.572	7.026	-18.709
C	0.959	7.289	-17.317
H	0.598	8.194	-17.801
C	9.474	-1.136	-18.229
H	9.782	-1.539	-19.195
H	9.571	-0.050	-18.303
C	8.057	-1.489	-17.936
N	7.489	-2.721	-18.228
H	7.939	-3.482	-18.740
C	6.238	-2.749	-17.730
H	5.559	-3.579	-17.821
N	5.967	-1.592	-17.138
C	7.087	-0.794	-17.266
H	7.125	0.204	-16.861
C	6.050	3.700	-14.052
H	5.560	4.665	-14.228
H	5.770	3.383	-13.044
C	5.540	2.678	-15.026
N	4.975	2.985	-16.250
H	4.793	3.907	-16.662
C	4.508	1.857	-16.821
H	4.015	1.830	-17.782
N	4.744	0.818	-16.024
C	5.385	1.317	-14.902
H	5.672	0.673	-14.084
C	6.298	-2.805	-14.026
H	6.825	-2.471	-14.924
H	6.020	-1.907	-13.458
C	5.029	-3.533	-14.407
N	4.147	-2.988	-15.340
C	3.105	-3.811	-15.434
H	2.257	-3.678	-16.088
N	3.261	-4.853	-14.603
H	2.617	-5.656	-14.520
C	4.464	-4.701	-13.948
H	4.791	-5.417	-13.207
CU	4.272	-1.145	-16.199
O	4.466	-2.260	-20.134
H	5.145	-1.598	-20.398
H	4.943	-3.130	-20.190
O	3.315	2.848	-26.479
H	3.033	3.724	-26.157
H	2.803	2.211	-25.960
O	3.072	-1.463	-18.008
H	3.602	-1.748	-18.807
H	2.226	-1.121	-18.328
O	2.510	-0.624	-15.191
H	2.140	0.216	-15.508
H	1.782	-1.273	-15.201
C	12.635	-0.419	-20.515
C	5.614	10.841	-20.532
C	-1.008	1.739	-24.267
C	-0.887	2.948	-20.659
C	-2.134	6.895	-15.528
C	10.446	-1.674	-17.150
C	7.586	3.910	-14.083
C	7.266	-3.618	-13.172

110

3hNA

C	11.930	-0.655	-21.866
H	12.121	-1.681	-22.192
H	12.386	0.009	-22.602
C	10.425	-0.465	-21.763
C	9.800	0.784	-21.641
H	10.392	1.695	-21.686
C	8.422	0.883	-21.430
H	7.965	1.862	-21.326
C	7.624	-0.263	-21.339
O	6.273	-0.218	-21.079
H	5.904	0.704	-21.047
C	8.220	-1.515	-21.506
H	7.604	-2.409	-21.462
C	9.597	-1.594	-21.711
H	10.073	-2.561	-21.801
C	4.722	9.930	-19.656
H	3.673	10.206	-19.813
H	4.975	10.080	-18.609
C	4.855	8.432	-19.939
O	4.754	7.595	-19.060
O	4.988	8.084	-21.223
H	5.053	7.100	-21.256
C	-0.007	0.562	-24.460
H	0.225	0.452	-25.515
H	-0.452	-0.365	-24.097
H	0.903	0.763	-23.882
C	0.353	2.525	-19.775
H	0.535	1.443	-19.765
H	0.079	2.781	-18.744
C	1.625	3.219	-20.205
C	1.733	4.623	-20.169
C	2.741	2.485	-20.636
H	2.678	1.399	-20.666
C	2.903	5.248	-20.585
H	2.980	6.325	-20.577
C	3.935	3.093	-21.038
C	3.979	4.494	-21.007
O	0.750	5.453	-19.742
H	0.141	4.994	-19.141
N	5.077	2.327	-21.493
H	4.980	2.068	-22.477
H	5.917	2.904	-21.435
O	5.128	5.182	-21.337
H	5.461	4.818	-22.168
C	-0.796	7.660	-15.547
H	-0.577	8.009	-14.527
H	-0.910	8.550	-16.167
C	0.347	6.845	-16.064
C	0.804	5.678	-15.440
H	0.300	5.313	-14.550
C	1.900	4.980	-15.945
H	2.248	4.059	-15.485
C	2.562	5.489	-17.061
O	3.617	4.782	-17.601
H	3.893	5.262	-18.402
C	2.153	6.671	-17.662
H	2.710	7.069	-18.496
C	1.032	7.322	-17.180
H	0.690	8.229	-17.672
C	9.476	-1.139	-18.230
H	9.785	-1.539	-19.196
H	9.569	-0.053	-18.305
C	8.060	-1.498	-17.939
N	7.493	-2.728	-18.242
H	7.944	-3.487	-18.757

C	6.243	-2.763	-17.743
H	5.566	-3.593	-17.846
N	5.971	-1.611	-17.140
C	7.090	-0.811	-17.262
H	7.129	0.183	-16.846
C	6.057	3.685	-14.039
H	5.554	4.640	-14.228
H	5.787	3.380	-13.025
C	5.556	2.642	-14.993
N	5.006	2.939	-16.224
H	4.809	3.865	-16.625
C	4.538	1.810	-16.788
H	4.046	1.776	-17.749
N	4.765	0.778	-15.979
C	5.398	1.282	-14.856
H	5.677	0.642	-14.033
C	6.302	-2.808	-14.028
H	6.830	-2.473	-14.924
H	6.020	-1.910	-13.460
C	5.036	-3.542	-14.408
N	4.154	-3.009	-15.346
C	3.114	-3.834	-15.431
H	2.261	-3.709	-16.080
N	3.274	-4.870	-14.593
H	2.633	-5.674	-14.508
C	4.477	-4.708	-13.939
H	4.808	-5.416	-13.193
CU	4.283	-1.170	-16.192
O	4.531	-2.180	-20.188
H	5.193	-1.511	-20.483
H	5.002	-3.052	-20.231
O	3.147	-1.324	-18.068
H	3.664	-1.648	-18.859
H	2.259	-1.091	-18.373
O	2.507	-0.662	-15.205
H	2.174	0.210	-15.471
H	1.754	-1.279	-15.257
C	12.630	-0.420	-20.518
C	5.615	10.843	-20.534
C	-1.007	1.727	-24.285
C	-0.873	2.785	-20.675
C	-2.137	6.920	-15.544
C	10.450	-1.674	-17.150
C	7.590	3.911	-14.084
C	7.268	-3.618	-13.172

110

1

C	12.002	-0.663	-21.884
H	12.231	-1.679	-22.208
H	12.462	0.021	-22.599
C	10.499	-0.541	-21.814
C	9.818	0.673	-21.671
H	10.369	1.611	-21.689
C	8.441	0.695	-21.462
H	7.919	1.635	-21.330
C	7.712	-0.503	-21.400
O	6.373	-0.517	-21.160
H	6.025	0.419	-21.212
C	8.371	-1.721	-21.577
H	7.798	-2.642	-21.534
C	9.748	-1.719	-21.775
H	10.297	-2.648	-21.864
C	4.693	9.918	-19.653
H	3.647	10.221	-19.763
H	4.982	10.050	-18.610
C	4.759	8.413	-19.938
O	4.511	7.583	-19.087
O	5.017	8.048	-21.206
H	5.025	7.068	-21.202
C	0.062	0.598	-24.450
H	0.224	0.465	-25.518
H	-0.359	-0.329	-24.061
C	1.412	0.943	-23.813
O	2.114	1.887	-24.206
N	1.793	0.143	-22.803
H	1.145	-0.535	-22.402
H	2.639	0.377	-22.297
C	0.479	3.771	-19.978
H	0.328	3.644	-18.900
H	0.485	4.846	-20.176
C	1.804	3.218	-20.370
C	2.264	1.916	-19.953
C	2.665	4.035	-21.070
H	2.329	5.021	-21.374
C	3.527	1.462	-20.277
H	3.862	0.468	-19.984
C	3.973	3.649	-21.337
C	4.426	2.297	-21.010
O	1.378	1.198	-19.231
H	1.741	0.322	-19.018
N	4.875	4.523	-21.862
H	5.757	4.098	-22.152
H	4.524	5.166	-22.565
O	5.570	1.941	-21.412
C	-0.762	7.631	-15.645
H	-0.446	7.930	-14.637
H	-0.901	8.550	-16.219
C	0.307	6.797	-16.302
C	0.900	5.707	-15.653
H	0.552	5.422	-14.663
C	1.940	4.993	-16.242
H	2.406	4.155	-15.733
C	2.407	5.381	-17.498
O	3.464	4.685	-18.042
H	3.796	5.200	-18.798
C	1.810	6.441	-18.179
H	2.184	6.735	-19.154
C	0.769	7.138	-17.576
H	0.317	7.976	-18.103
C	9.480	-1.113	-18.254
H	9.811	-1.501	-19.220
H	9.580	-0.027	-18.310
C	8.056	-1.461	-18.005
N	7.505	-2.701	-18.285
H	7.966	-3.466	-18.776
C	6.219	-2.697	-17.862
H	5.547	-3.531	-17.974
N	5.909	-1.527	-17.333
C	7.044	-0.747	-17.420
H	7.059	0.267	-17.047
C	6.014	3.794	-14.024
H	5.546	4.768	-14.213
H	5.748	3.514	-13.001
C	5.451	2.758	-14.941
N	4.908	3.010	-16.187
H	4.806	3.902	-16.668
C	4.371	1.862	-16.663
H	3.880	1.796	-17.621
N	4.536	0.876	-15.790
C	5.206	1.427	-14.715
H	5.456	0.835	-13.847
C	6.307	-2.800	-14.044
H	6.816	-2.520	-14.973
H	6.073	-1.866	-13.514
C	5.005	-3.500	-14.350
N	4.040	-2.856	-15.117
C	3.007	-3.681	-15.214
H	2.095	-3.483	-15.754
N	3.245	-4.824	-14.541
H	2.615	-5.635	-14.494
C	4.503	-4.726	-13.984
H	4.904	-5.518	-13.368
CU	4.388	-1.095	-15.972
O	4.455	-2.331	-20.171
H	5.152	-1.679	-20.389
H	4.957	-3.179	-20.233
O	3.356	3.207	-26.304
H	3.148	4.137	-26.099
H	2.838	2.695	-25.648
C	12.647	-0.423	-20.512
C	5.581	10.827	-20.549
C	-0.967	1.737	-24.266
C	-0.729	3.149	-20.691
C	-2.124	6.928	-15.549
C	10.434	-1.665	-17.163
C	7.553	3.963	-14.096
C	7.267	-3.609	-13.180

110

TS(1,2)

C	12.007	-0.659	-21.884

H	12.259	-1.666	-22.220
H	12.443	0.044	-22.593
C	10.502	-0.576	-21.801
C	9.800	0.627	-21.678
H	10.330	1.575	-21.724
C	8.428	0.628	-21.445
H	7.890	1.559	-21.322
C	7.724	-0.581	-21.343
O	6.382	-0.599	-21.076
H	6.074	0.340	-21.036
C	8.401	-1.792	-21.499
H	7.855	-2.726	-21.406
C	9.777	-1.767	-21.715
H	10.351	-2.685	-21.762
C	4.714	9.919	-19.639
H	3.663	10.192	-19.777
H	4.988	10.076	-18.597
C	4.844	8.423	-19.911
O	4.803	7.602	-19.017
O	4.908	8.065	-21.198
H	4.854	7.089	-21.226
C	-0.238	0.434	-24.585
H	-0.245	0.278	-25.654
H	-0.770	-0.406	-24.138
C	1.205	0.430	-24.209
O	1.987	1.383	-24.383
N	1.593	-0.784	-23.790
H	0.900	-1.371	-23.328
H	2.538	-0.935	-23.461
C	0.542	3.934	-20.152
H	0.404	4.033	-19.065
H	0.531	4.954	-20.561
C	1.892	3.347	-20.450
C	3.053	4.206	-20.313
C	2.113	2.074	-20.923
H	1.308	1.365	-21.096
C	4.345	3.801	-20.546
H	5.170	4.493	-20.445
C	3.401	1.701	-21.304
C	4.591	2.491	-21.026
O	2.880	5.513	-20.001
H	2.069	5.628	-19.476
N	3.588	0.682	-22.169
H	4.574	0.590	-22.409
H	3.033	0.830	-23.013
O	5.714	2.005	-21.338
C	-0.864	7.748	-15.532
H	-0.674	8.133	-14.519
H	-1.023	8.612	-16.177
C	0.314	6.991	-16.013
C	0.971	6.046	-15.218
H	0.561	5.793	-14.242
C	2.163	5.473	-15.640
H	2.712	4.769	-15.023

C	2.704	5.891	-16.855
O	3.918	5.421	-17.235
H	4.263	6.047	-17.902
C	2.040	6.810	-17.672
H	2.541	7.217	-18.539
C	0.837	7.342	-17.249
H	0.330	8.085	-17.859
C	9.478	-1.112	-18.252
H	9.809	-1.497	-19.218
H	9.574	-0.025	-18.307
C	8.057	-1.467	-17.998
N	7.516	-2.718	-18.249
H	7.978	-3.488	-18.731
C	6.232	-2.715	-17.821
H	5.565	-3.555	-17.910
N	5.914	-1.536	-17.318
C	7.042	-0.749	-17.425
H	7.052	0.273	-17.075
C	6.012	3.779	-13.998
H	5.537	4.752	-14.179
H	5.754	3.490	-12.975
C	5.448	2.746	-14.920
N	4.928	3.015	-16.171
H	4.813	3.937	-16.598
C	4.394	1.873	-16.666
H	3.921	1.808	-17.635
N	4.539	0.878	-15.801
C	5.194	1.415	-14.709
H	5.429	0.811	-13.846
C	6.310	-2.799	-14.047
H	6.820	-2.517	-14.974
H	6.078	-1.866	-13.516
C	5.007	-3.495	-14.356
N	4.043	-2.846	-15.119
C	3.009	-3.670	-15.222
H	2.099	-3.467	-15.763
N	3.247	-4.817	-14.556
H	2.616	-5.628	-14.511
C	4.505	-4.724	-13.998
H	4.906	-5.519	-13.387
CU	4.378	-1.089	-15.982
O	4.363	-2.470	-20.070
H	5.005	-1.744	-20.154
H	4.941	-3.265	-20.185
O	3.311	3.167	-26.285
H	3.116	4.091	-26.039
H	2.796	2.623	-25.661
C	12.652	-0.417	-20.513
C	5.591	10.829	-20.539
C	-1.006	1.727	-24.287
C	-0.687	3.194	-20.720
C	-2.150	6.928	-15.528
C	10.435	-1.663	-17.166
C	7.550	3.957	-14.080

C	7.268	-3.611	-13.184

110

2

C	12.005	-0.636	-21.888
H	12.290	-1.622	-22.256
H	12.406	0.102	-22.581
C	10.499	-0.624	-21.784
C	9.716	0.534	-21.691
H	10.184	1.512	-21.780
C	8.349	0.447	-21.429
H	7.739	1.342	-21.342
C	7.741	-0.806	-21.259
O	6.430	-0.938	-20.915
H	5.956	-0.095	-21.152
C	8.499	-1.968	-21.401
H	8.010	-2.927	-21.275
C	9.863	-1.860	-21.648
H	10.494	-2.742	-21.675
C	4.715	9.902	-19.647
H	3.667	10.206	-19.743
H	5.017	10.018	-18.605
C	4.772	8.405	-19.965
O	4.588	7.560	-19.110
O	4.921	8.078	-21.256
H	4.791	7.109	-21.323
C	0.018	0.558	-24.491
H	0.125	0.403	-25.564
H	-0.416	-0.352	-24.076
C	1.409	0.843	-23.947
O	2.082	1.827	-24.280
N	1.884	-0.113	-23.131
H	1.247	-0.771	-22.684
H	2.804	0.016	-22.736
C	0.575	3.904	-20.179
H	0.450	4.028	-19.097
H	0.580	4.920	-20.594
C	1.877	3.200	-20.464
C	3.026	3.897	-20.999
C	2.009	1.850	-20.201
H	1.178	1.304	-19.758
C	4.144	3.237	-21.429
H	4.967	3.773	-21.889
C	3.163	1.146	-20.566
C	4.246	1.826	-21.295
O	3.060	5.265	-21.058
H	2.199	5.604	-20.776
N	3.271	-0.181	-20.355
H	4.043	-0.726	-20.708
H	2.591	-0.691	-19.811
O	5.213	1.163	-21.770
C	-0.768	7.645	-15.635
H	-0.445	7.929	-14.624

H	-0.917	8.573	-16.190
C	0.296	6.832	-16.320
C	0.850	5.684	-15.739
H	0.469	5.335	-14.782
C	1.891	4.994	-16.352
H	2.324	4.108	-15.898
C	2.407	5.469	-17.559
O	3.466	4.799	-18.120
H	3.822	5.356	-18.837
C	1.847	6.589	-18.174
H	2.278	6.976	-19.089
C	0.798	7.257	-17.552
H	0.379	8.143	-18.024
C	9.474	-1.108	-18.244
H	9.802	-1.488	-19.211
H	9.564	-0.020	-18.293
C	8.057	-1.471	-17.980
N	7.532	-2.735	-18.197
H	7.996	-3.503	-18.680
C	6.253	-2.741	-17.759
H	5.596	-3.591	-17.827
N	5.917	-1.549	-17.293
C	7.034	-0.751	-17.423
H	7.033	0.280	-17.098
C	6.010	3.785	-14.006
H	5.540	4.764	-14.163
H	5.748	3.473	-12.992
C	5.441	2.776	-14.953
N	4.902	3.052	-16.198
H	4.824	3.950	-16.671
C	4.357	1.914	-16.696
H	3.866	1.868	-17.656
N	4.509	0.914	-15.836
C	5.180	1.444	-14.750
H	5.415	0.838	-13.888
C	6.314	-2.794	-14.051
H	6.825	-2.509	-14.976
H	6.083	-1.862	-13.516
C	5.009	-3.486	-14.362
N	4.040	-2.834	-15.119
C	3.008	-3.660	-15.222
H	2.094	-3.459	-15.758
N	3.249	-4.810	-14.562
H	2.617	-5.621	-14.516
C	4.508	-4.717	-14.008
H	4.913	-5.513	-13.401
CU	4.365	-1.065	-15.981
O	4.366	-2.604	-19.938
H	5.083	-1.976	-19.739
H	4.913	-3.402	-20.181
O	3.360	3.205	-26.337
H	3.183	4.138	-26.113
H	2.856	2.699	-25.668
C	12.655	-0.412	-20.515
C	5.591	10.823	-20.540
C	-0.963	1.726	-24.281
C	-0.681	3.191	-20.725
C	-2.124	6.933	-15.541
C	10.435	-1.663	-17.166
C	7.549	3.957	-14.076
C	7.270	-3.610	-13.189

110

TS(2,3)

C	12.023	-0.635	-21.903
H	12.246	-1.650	-22.234
H	12.499	0.052	-22.603
C	10.521	-0.503	-21.843
C	9.841	0.713	-21.696
H	10.385	1.652	-21.754
C	8.475	0.731	-21.414
H	7.940	1.668	-21.283
C	7.788	-0.476	-21.264
O	6.490	-0.526	-20.843
H	6.107	0.365	-20.722
C	8.418	-1.693	-21.512
H	7.848	-2.613	-21.422
C	9.778	-1.686	-21.795
H	10.319	-2.615	-21.923
C	4.714	9.897	-19.659
H	3.661	10.168	-19.789
H	4.986	10.028	-18.612
C	4.847	8.407	-19.976
O	4.752	7.542	-19.128
O	4.988	8.110	-21.277
H	5.051	7.139	-21.352
C	-0.023	0.542	-24.529
H	0.078	0.399	-25.601
H	-0.461	-0.369	-24.118
C	1.368	0.813	-23.986
O	2.068	1.772	-24.335
N	1.819	-0.124	-23.135
H	1.171	-0.767	-22.683
H	2.722	0.048	-22.719
C	0.285	2.937	-19.662
H	0.190	2.036	-19.046
H	0.155	3.752	-18.938
C	1.636	2.971	-20.270
C	2.226	4.242	-20.558
C	2.332	1.802	-20.542
H	1.858	0.840	-20.364
C	3.500	4.359	-21.036
H	3.947	5.331	-21.204
C	3.599	1.882	-21.093
C	4.281	3.179	-21.244
O	1.529	5.361	-20.278
H	0.635	5.147	-19.975
N	4.343	0.799	-21.471
H	4.018	-0.149	-21.326
H	4.985	0.956	-22.240
O	5.499	3.175	-21.501
C	-0.807	7.657	-15.582
H	-0.535	7.983	-14.568
H	-0.965	8.562	-16.171
C	0.306	6.867	-16.190
C	0.828	5.718	-15.589
H	0.382	5.346	-14.670
C	1.916	5.053	-16.145
H	2.324	4.154	-15.694
C	2.498	5.570	-17.300
O	3.574	4.916	-17.842
H	3.921	5.487	-18.551
C	1.992	6.714	-17.915
H	2.458	7.092	-18.812
C	0.887	7.340	-17.366
H	0.482	8.228	-17.847
C	9.476	-1.116	-18.250
H	9.807	-1.500	-19.217
H	9.569	-0.028	-18.304
C	8.056	-1.474	-17.994
N	7.514	-2.724	-18.255
H	7.982	-3.496	-18.728
C	6.230	-2.723	-17.828
H	5.566	-3.566	-17.920
N	5.910	-1.547	-17.321
C	7.038	-0.759	-17.421
H	7.046	0.261	-17.065
C	6.012	3.784	-14.008
H	5.538	4.758	-14.181
H	5.753	3.488	-12.987
C	5.447	2.757	-14.935
N	4.898	3.023	-16.175
H	4.780	3.925	-16.640
C	4.363	1.879	-16.663
H	3.864	1.820	-17.619
N	4.536	0.882	-15.804
C	5.209	1.423	-14.725
H	5.463	0.820	-13.865
C	6.309	-2.800	-14.045
H	6.819	-2.518	-14.972
H	6.076	-1.867	-13.514
C	5.007	-3.498	-14.353
N	4.042	-2.854	-15.119
C	3.009	-3.678	-15.217
H	2.096	-3.479	-15.756
N	3.247	-4.822	-14.545
H	2.617	-5.633	-14.498
C	4.505	-4.725	-13.988
H	4.907	-5.517	-13.372
CU	4.375	-1.088	-15.972
O	4.469	-2.329	-20.183
H	5.217	-1.703	-20.193

H	4.944	-3.193	-20.265
O	3.360	3.220	-26.321
H	3.179	4.153	-26.098
H	2.835	2.711	-25.671
C	12.651	-0.417	-20.518
C	5.588	10.829	-20.540
C	-0.991	1.718	-24.300
C	-0.894	2.948	-20.657
C	-2.137	6.900	-15.519
C	10.436	-1.665	-17.166
C	7.550	3.960	-14.086
C	7.267	-3.611	-13.182

110

3

C	11.932	-0.664	-21.866
H	12.092	-1.702	-22.171
H	12.415	-0.030	-22.612
C	10.434	-0.413	-21.781
C	9.875	0.866	-21.655
H	10.514	1.745	-21.674
C	8.502	1.038	-21.476
H	8.095	2.036	-21.352
C	7.641	-0.062	-21.435
O	6.285	0.058	-21.248
H	6.001	0.998	-21.206
C	8.172	-1.346	-21.583
H	7.506	-2.203	-21.540
C	9.548	-1.499	-21.752
H	9.967	-2.495	-21.834
C	4.703	9.908	-19.664
H	3.651	10.172	-19.817
H	4.956	10.071	-18.617
C	4.847	8.402	-19.927
O	4.733	7.603	-19.010
O	4.988	8.024	-21.188
H	5.031	7.017	-21.215
C	0.056	0.564	-24.491
H	0.224	0.448	-25.560
H	-0.379	-0.367	-24.125
C	1.423	0.849	-23.854
O	2.148	1.800	-24.182
N	1.822	-0.064	-22.949
H	1.167	-0.744	-22.565
H	2.731	0.026	-22.515
C	0.332	2.570	-19.820
H	0.561	1.499	-19.814
H	0.064	2.813	-18.784
C	1.559	3.318	-20.271
C	1.650	4.752	-20.168
C	2.642	2.626	-20.816
H	2.564	1.549	-20.926
C	2.787	5.410	-20.550
H	2.867	6.479	-20.434
C	3.799	3.272	-21.247
C	3.929	4.718	-21.036
O	0.627	5.508	-19.704
H	0.065	5.004	-19.092
N	4.844	2.610	-21.887
H	4.518	2.028	-22.658
H	5.536	3.285	-22.223
O	5.022	5.296	-21.279
C	-0.832	7.689	-15.548
H	-0.610	8.037	-14.528
H	-0.970	8.579	-16.164
C	0.310	6.900	-16.084
C	0.805	5.743	-15.470
H	0.313	5.355	-14.583
C	1.931	5.097	-15.974
H	2.325	4.195	-15.515
C	2.573	5.652	-17.078
O	3.692	5.052	-17.588
H	4.057	5.690	-18.232
C	2.087	6.796	-17.698
H	2.616	7.213	-18.538
C	0.948	7.399	-17.213
H	0.561	8.288	-17.705
C	9.475	-1.121	-18.252
H	9.809	-1.506	-19.218
H	9.569	-0.033	-18.311
C	8.052	-1.477	-18.005
N	7.504	-2.719	-18.292
H	7.972	-3.490	-18.764
C	6.216	-2.717	-17.871
H	5.547	-3.554	-17.985
N	5.902	-1.550	-17.341
C	7.035	-0.767	-17.423
H	7.048	0.246	-17.046
C	6.016	3.781	-14.008
H	5.538	4.749	-14.199
H	5.761	3.501	-12.983
C	5.457	2.736	-14.917
N	4.898	2.996	-16.152
H	4.752	3.906	-16.597
C	4.369	1.847	-16.633
H	3.864	1.779	-17.584
N	4.558	0.852	-15.774
C	5.234	1.399	-14.700
H	5.504	0.799	-13.843
C	6.305	-2.803	-14.043
H	6.812	-2.523	-14.972
H	6.069	-1.871	-13.513
C	5.005	-3.508	-14.346
N	4.042	-2.871	-15.119
C	3.008	-3.696	-15.212
H	2.097	-3.501	-15.754
N	3.245	-4.833	-14.529
H	2.616	-5.644	-14.483
C	4.503	-4.732	-13.971
H	4.903	-5.518	-13.346
CU	4.384	-1.109	-15.966
O	4.596	-2.185	-20.318
H	5.230	-1.456	-20.439
H	5.143	-3.003	-20.331
O	3.343	3.222	-26.278
H	3.163	4.162	-26.081
H	2.815	2.730	-25.618
C	12.636	-0.424	-20.518
C	5.584	10.828	-20.551
C	-0.965	1.710	-24.297
C	-0.916	2.781	-20.702
C	-2.151	6.921	-15.538
C	10.433	-1.666	-17.163
C	7.553	3.962	-14.095
C	7.267	-3.610	-13.179

110

4

C	12.051	-0.639	-21.907
H	12.277	-1.658	-22.225
H	12.547	0.038	-22.604
C	10.548	-0.506	-21.897
C	9.863	0.682	-21.609
H	10.410	1.612	-21.477
C	8.477	0.684	-21.471
H	7.943	1.602	-21.246
C	7.750	-0.504	-21.616
O	6.401	-0.538	-21.460
H	6.099	0.158	-20.814
C	8.410	-1.684	-21.961
H	7.831	-2.594	-22.090
C	9.793	-1.665	-22.102
H	10.331	-2.568	-22.367
C	4.712	9.898	-19.648
H	3.666	10.215	-19.725
H	5.026	9.996	-18.608
C	4.747	8.404	-19.989
O	4.496	7.545	-19.166
O	4.970	8.099	-21.277
H	4.892	7.127	-21.357
C	0.113	0.616	-24.430
H	0.311	0.488	-25.494
H	-0.292	-0.323	-24.054
C	1.428	1.009	-23.747
O	2.103	1.980	-24.123
N	1.799	0.241	-22.709
H	1.165	-0.458	-22.322
H	2.621	0.515	-22.184
C	0.610	3.793	-20.191
H	0.433	4.090	-19.152
H	0.819	4.719	-20.734
C	1.781	2.848	-20.214

C	3.009	3.205	-20.879
C	1.722	1.649	-19.515
H	0.794	1.363	-19.023
C	4.140	2.436	-20.748
H	5.064	2.744	-21.226
C	2.843	0.812	-19.398
C	4.138	1.269	-19.926
O	3.126	4.382	-21.571
H	2.312	4.561	-22.070
N	2.837	-0.375	-18.745
H	3.626	-1.012	-18.877
H	1.957	-0.769	-18.440
O	5.178	0.621	-19.612
C	-0.758	7.636	-15.652
H	-0.429	7.929	-14.646
H	-0.904	8.559	-16.218
C	0.300	6.805	-16.331
C	0.899	5.709	-15.698
H	0.564	5.418	-14.705
C	1.925	4.994	-16.307
H	2.392	4.150	-15.809
C	2.377	5.386	-17.568
O	3.421	4.687	-18.131
H	3.759	5.207	-18.881
C	1.771	6.450	-18.236
H	2.125	6.738	-19.220
C	0.743	7.149	-17.612
H	0.284	7.989	-18.129
C	9.472	-1.121	-18.269
H	9.804	-1.511	-19.236
H	9.567	-0.034	-18.331
C	8.047	-1.475	-18.015
N	7.508	-2.726	-18.277
H	7.970	-3.491	-18.767
C	6.231	-2.734	-17.829
H	5.571	-3.580	-17.919
N	5.914	-1.564	-17.307
C	7.035	-0.768	-17.422
H	7.034	0.255	-17.078
C	6.017	3.791	-13.971
H	5.545	4.767	-14.142
H	5.770	3.501	-12.946
C	5.440	2.764	-14.883
N	4.847	3.039	-16.101
H	4.767	3.932	-16.581
C	4.297	1.900	-16.578
H	3.754	1.860	-17.503
N	4.502	0.896	-15.736
C	5.211	1.428	-14.675
H	5.496	0.820	-13.829
C	6.315	-2.797	-14.056
H	6.826	-2.518	-14.983
H	6.084	-1.862	-13.527
C	5.011	-3.491	-14.364
N	4.042	-2.846	-15.127
C	3.009	-3.672	-15.217
H	2.091	-3.474	-15.747
N	3.249	-4.816	-14.546
H	2.619	-5.627	-14.494
C	4.510	-4.718	-13.996
H	4.914	-5.509	-13.381
CU	4.368	-1.066	-15.995
O	4.517	-2.330	-20.197
H	5.195	-1.693	-20.500
H	5.017	-3.184	-20.219
O	3.340	3.209	-26.312
H	3.179	4.151	-26.111
H	2.846	2.730	-25.615
C	12.657	-0.413	-20.515
C	5.589	10.823	-20.539
C	-0.954	1.725	-24.278
C	-0.689	3.180	-20.732
C	-2.120	6.933	-15.543
C	10.429	-1.662	-17.174
C	7.553	3.959	-14.073
C	7.270	-3.610	-13.190

107

TS(4,5)

C	12.015	-0.639	-21.897
H	12.218	-1.664	-22.216
H	12.508	0.026	-22.607
C	10.513	-0.461	-21.858
C	9.881	0.769	-21.633
H	10.465	1.682	-21.570
C	8.499	0.834	-21.465
H	8.015	1.791	-21.291
C	7.725	-0.328	-21.517
O	6.368	-0.305	-21.293
H	6.142	0.538	-20.876
C	8.323	-1.553	-21.800
H	7.707	-2.446	-21.854
C	9.704	-1.597	-21.969
H	10.200	-2.538	-22.173
C	4.709	9.907	-19.647
H	3.663	10.215	-19.742
H	5.010	10.015	-18.604
C	4.773	8.414	-19.977
O	4.565	7.551	-19.150
O	4.985	8.113	-21.269
H	4.974	7.139	-21.338
C	0.096	0.604	-24.444
H	0.285	0.482	-25.511
H	-0.320	-0.334	-24.074
C	1.424	0.975	-23.775
O	2.078	1.970	-24.124
N	1.821	0.137	-22.800
H	1.183	-0.572	-22.439
H	2.651	0.358	-22.263
C	0.614	3.810	-20.210
H	0.369	4.397	-19.319
H	1.023	4.513	-20.945
C	1.567	2.724	-19.815
C	2.789	2.466	-20.516
C	1.188	1.887	-18.773
H	0.265	2.100	-18.244
C	3.582	1.404	-20.150
H	4.509	1.244	-20.683
C	1.918	0.751	-18.437
C	3.213	0.508	-19.089
O	3.239	3.289	-21.509
H	2.508	3.586	-22.077
N	1.532	-0.114	-17.469
H	2.067	-0.968	-17.377
H	0.582	-0.108	-17.124
O	3.953	-0.407	-18.654
C	-0.758	7.635	-15.646
H	-0.428	7.916	-14.638
H	-0.905	8.564	-16.201
C	0.298	6.814	-16.337
C	0.887	5.698	-15.731
H	0.543	5.383	-14.750
C	1.917	4.998	-16.351
H	2.377	4.138	-15.878
C	2.381	5.426	-17.593
O	3.433	4.752	-18.168
H	3.800	5.325	-18.854
C	1.781	6.508	-18.236
H	2.146	6.825	-19.206
C	0.750	7.192	-17.603
H	0.296	8.046	-18.099
C	9.507	-1.115	-18.286
H	9.853	-1.505	-19.246
H	9.605	-0.028	-18.342
C	8.087	-1.468	-18.043
N	7.533	-2.704	-18.333
H	7.980	-3.464	-18.843
C	6.280	-2.732	-17.816
H	5.593	-3.552	-17.940
N	6.009	-1.592	-17.217
C	7.112	-0.788	-17.366
H	7.132	0.221	-16.979
C	6.023	3.791	-13.946
H	5.550	4.770	-14.093
H	5.788	3.478	-12.926
C	5.448	2.788	-14.876
N	4.934	3.095	-16.118
H	4.889	4.003	-16.567
C	4.411	1.969	-16.653
H	3.945	1.967	-17.619
N	4.550	0.947	-15.822
C	5.198	1.451	-14.711

H	5.443	0.818	-13.871
C	6.315	-2.785	-14.055
H	6.829	-2.503	-14.978
H	6.089	-1.850	-13.523
C	5.005	-3.465	-14.379
N	4.042	-2.816	-15.152
C	3.017	-3.650	-15.260
H	2.110	-3.461	-15.811
N	3.249	-4.797	-14.589
H	2.623	-5.611	-14.546
C	4.502	-4.695	-14.022
H	4.904	-5.487	-13.407
CU	4.376	-1.039	-16.109
O	4.517	-2.426	-20.242
H	5.113	-1.661	-20.251
H	5.114	-3.212	-20.334
C	12.652	-0.414	-20.516
C	5.591	10.827	-20.535
C	-0.956	1.725	-24.279
C	-0.689	3.199	-20.742
C	-2.120	6.933	-15.543
C	10.440	-1.662	-17.174
C	7.556	3.959	-14.068
C	7.266	-3.608	-13.190

110

5

C	11.955	-0.644	-21.871
H	12.128	-1.677	-22.186
H	12.439	0.001	-22.606
C	10.454	-0.415	-21.793
C	9.871	0.850	-21.635
H	10.491	1.742	-21.625
C	8.493	0.986	-21.461
H	8.061	1.973	-21.321
C	7.659	-0.135	-21.462
O	6.298	-0.059	-21.286
H	6.004	0.857	-21.159
C	8.214	-1.401	-21.651
H	7.565	-2.273	-21.647
C	9.593	-1.519	-21.811
H	10.038	-2.499	-21.929
C	4.716	9.895	-19.650
H	3.668	10.204	-19.734
H	5.022	9.993	-18.608
C	4.776	8.405	-19.997
O	4.555	7.534	-19.180
O	4.978	8.119	-21.293
H	4.933	7.148	-21.381
C	0.104	0.607	-24.442
H	0.321	0.506	-25.506
H	-0.319	-0.337	-24.101
C	1.413	0.945	-23.723
O	2.048	1.996	-23.942

N	1.842	0.024	-22.850
H	1.197	-0.686	-22.502
H	2.640	0.268	-22.272
C	0.714	3.603	-20.190
H	0.552	4.425	-19.486
H	1.326	4.016	-21.004
C	1.388	2.460	-19.443
C	2.428	1.615	-19.953
C	0.846	2.123	-18.210
H	0.085	2.771	-17.790
C	2.805	0.458	-19.293
H	3.579	-0.189	-19.693
C	1.160	0.935	-17.541
C	2.172	0.051	-18.082
O	3.146	1.913	-21.096
H	2.649	2.473	-21.718
N	0.519	0.566	-16.394
H	0.666	-0.405	-16.129
H	-0.445	0.884	-16.340
O	2.452	-1.034	-17.448
C	-0.761	7.640	-15.658
H	-0.413	7.902	-14.649
H	-0.917	8.580	-16.190
C	0.285	6.834	-16.386
C	0.862	5.683	-15.834
H	0.517	5.321	-14.867
C	1.887	5.005	-16.485
H	2.337	4.119	-16.052
C	2.360	5.490	-17.706
O	3.418	4.849	-18.305
H	3.834	5.497	-18.902
C	1.758	6.598	-18.303
H	2.116	6.950	-19.265
C	0.734	7.263	-17.638
H	0.286	8.141	-18.097
C	9.497	-1.116	-18.269
H	9.835	-1.511	-19.229
H	9.601	-0.030	-18.330
C	8.073	-1.457	-18.024
N	7.511	-2.696	-18.298
H	7.964	-3.475	-18.773
C	6.230	-2.679	-17.857
H	5.543	-3.503	-17.959
N	5.939	-1.508	-17.327
C	7.073	-0.735	-17.428
H	7.099	0.279	-17.054
C	6.018	3.769	-13.975
H	5.535	4.750	-14.080
H	5.781	3.413	-12.969
C	5.448	2.800	-14.957
N	4.914	3.147	-16.185
H	4.856	4.066	-16.611
C	4.379	2.036	-16.749
H	3.896	2.040	-17.712

N	4.533	0.989	-15.952
C	5.197	1.456	-14.836
H	5.429	0.801	-14.010
C	6.324	-2.773	-14.072
H	6.854	-2.486	-14.986
H	6.096	-1.841	-13.537
C	5.012	-3.445	-14.414
N	4.066	-2.794	-15.203
C	3.033	-3.613	-15.320
H	2.148	-3.401	-15.899
N	3.243	-4.760	-14.639
H	2.607	-5.567	-14.587
C	4.493	-4.668	-14.057
H	4.874	-5.463	-13.434
CU	4.171	-1.027	-16.336
O	4.638	-2.230	-20.330
H	5.301	-1.542	-20.516
H	5.149	-3.074	-20.316
O	3.279	3.260	-26.202
H	3.105	4.211	-26.059
H	2.809	2.817	-25.465
C	12.647	-0.413	-20.517
C	5.592	10.821	-20.538
C	-0.952	1.724	-24.279
C	-0.664	3.168	-20.735
C	-2.119	6.934	-15.545
C	10.437	-1.664	-17.168
C	7.552	3.956	-14.073
C	7.263	-3.603	-13.197

103

1NA

C	12.005	-0.661	-21.889
H	12.234	-1.677	-22.215
H	12.467	0.025	-22.601
C	10.503	-0.539	-21.820
C	9.823	0.675	-21.678
H	10.375	1.612	-21.696
C	8.446	0.697	-21.469
H	7.925	1.638	-21.336
C	7.717	-0.500	-21.408
O	6.376	-0.514	-21.172
H	6.027	0.416	-21.250
C	8.375	-1.719	-21.584
H	7.801	-2.639	-21.541
C	9.752	-1.717	-21.781
H	10.301	-2.646	-21.872
C	4.689	9.926	-19.652
H	3.643	10.227	-19.769
H	4.972	10.069	-18.610
C	4.761	8.420	-19.919
O	4.506	7.600	-19.060
O	5.034	8.037	-21.179
H	5.070	7.057	-21.147

C 0.062 0.606 -24.413
H 0.295 0.478 -25.469
H -0.353 -0.324 -24.027
H 0.966 0.846 -23.842
C 0.489 3.771 -19.995
H 0.359 3.629 -18.916
H 0.490 4.849 -20.176
C 1.809 3.222 -20.416
C 2.210 1.855 -20.165
C 2.714 4.086 -21.001
H 2.418 5.114 -21.189
C 3.467 1.407 -20.509
H 3.751 0.368 -20.356
C 4.014 3.691 -21.290
C 4.420 2.297 -21.093
O 1.280 1.075 -19.580
H 1.551 0.142 -19.597
N 4.951 4.587 -21.727
H 5.826 4.154 -22.026
H 4.630 5.264 -22.413
O 5.576 1.955 -21.473
C -0.762 7.629 -15.638
H -0.451 7.927 -14.628
H -0.898 8.549 -16.210
C 0.308 6.795 -16.292
C 0.895 5.700 -15.647
H 0.539 5.409 -14.661
C 1.937 4.989 -16.234
H 2.398 4.145 -15.731
C 2.414 5.388 -17.483
O 3.475 4.700 -18.025
H 3.808 5.219 -18.777
C 1.822 6.453 -18.161
H 2.206 6.755 -19.128
C 0.777 7.146 -17.561
H 0.327 7.987 -18.084
C 9.478 -1.119 -18.255
H 9.806 -1.512 -19.219
H 9.581 -0.032 -18.318
C 8.055 -1.465 -18.000
N 7.502 -2.703 -18.286
H 7.961 -3.463 -18.786
C 6.221 -2.703 -17.853
H 5.547 -3.534 -17.969
N 5.915 -1.538 -17.310
C 7.049 -0.756 -17.400
H 7.068 0.254 -17.019
C 6.014 3.791 -14.027
H 5.544 4.765 -14.209
H 5.750 3.503 -13.006
C 5.452 2.757 -14.948
N 4.926 3.006 -16.203
H 4.826 3.897 -16.684
C 4.388 1.857 -16.681
H 3.913 1.785 -17.648
N 4.535 0.877 -15.800
C 5.194 1.430 -14.719
H 5.428 0.841 -13.844
C 6.307 -2.799 -14.044
H 6.814 -2.521 -14.973
H 6.075 -1.864 -13.515
C 5.004 -3.499 -14.347
N 4.038 -2.854 -15.112
C 3.006 -3.679 -15.214
H 2.095 -3.482 -15.755
N 3.245 -4.824 -14.543
H 2.616 -5.637 -14.501
C 4.503 -4.727 -13.985
H 4.905 -5.520 -13.371
CU 4.383 -1.094 -15.976
O 4.465 -2.339 -20.158
H 5.160 -1.682 -20.363
H 4.966 -3.187 -20.243
C 12.646 -0.424 -20.515
C 5.579 10.828 -20.551
C -0.976 1.742 -24.261
C -0.728 3.153 -20.692
C -2.125 6.928 -15.550
C 10.434 -1.667 -17.164
C 7.554 3.963 -14.098
C 7.267 -3.608 -13.180

103

TS(1NA,2NA)

C 12.010 -0.651 -21.888
H 12.273 -1.650 -22.235
H 12.437 0.066 -22.589
C 10.506 -0.591 -21.800
C 9.779 0.596 -21.671
H 10.288 1.556 -21.719
C 8.407 0.565 -21.435
H 7.853 1.484 -21.300
C 7.731 -0.660 -21.341
O 6.387 -0.725 -21.095
H 6.034 0.196 -21.097
C 8.436 -1.855 -21.493
H 7.908 -2.799 -21.402
C 9.810 -1.799 -21.711
H 10.404 -2.703 -21.764
C 4.704 9.921 -19.639
H 3.657 10.224 -19.744
H 5.001 10.050 -18.598
C 4.777 8.426 -19.938
O 4.595 7.584 -19.079
O 4.961 8.086 -21.219
H 4.933 7.110 -21.261
C 0.068 0.593 -24.435
H 0.295 0.466 -25.491
H -0.336 -0.335 -24.035
H 0.974 0.835 -23.875
C 0.538 3.853 -20.273
H 0.420 3.979 -19.193
H 0.572 4.862 -20.713
C 1.828 3.162 -20.614
C 2.919 3.593 -19.813
C 2.160 2.309 -21.653
H 1.466 1.867 -22.347
C 4.210 3.201 -19.997
H 4.863 3.606 -19.261
C 3.481 1.958 -21.926
C 4.577 2.325 -21.040
O 2.724 4.425 -18.784
H 1.803 4.702 -18.683
N 3.838 1.327 -23.060
H 4.811 1.043 -23.133
H 3.147 0.874 -23.640
O 5.716 1.840 -21.281
C -0.873 7.760 -15.550
H -0.695 8.177 -14.548
H -1.030 8.603 -16.222
C 0.318 6.994 -15.982
C 0.995 6.145 -15.104
H 0.570 5.943 -14.123
C 2.226 5.611 -15.453
H 2.793 4.973 -14.782
C 2.782 5.971 -16.676
O 4.055 5.581 -16.936
H 4.342 6.092 -17.714
C 2.059 6.718 -17.615
H 2.491 6.938 -18.582
C 0.826 7.229 -17.256
H 0.270 7.857 -17.948
C 9.480 -1.112 -18.251
H 9.810 -1.496 -19.218
H 9.576 -0.025 -18.305
C 8.060 -1.467 -17.996
N 7.519 -2.717 -18.254
H 7.979 -3.479 -18.750
C 6.237 -2.720 -17.823
H 5.567 -3.555 -17.927
N 5.920 -1.545 -17.310
C 7.046 -0.755 -17.414
H 7.054 0.265 -17.058
C 6.013 3.774 -14.006
H 5.536 4.745 -14.181
H 5.757 3.473 -12.986
C 5.450 2.744 -14.934
N 4.896 3.021 -16.169
H 4.757 3.954 -16.568
C 4.351 1.883 -16.659
H 3.857 1.824 -17.616
N 4.526 0.880 -15.807

C	5.208	1.410	-14.729
H	5.465	0.799	-13.877
C	6.311	-2.798	-14.049
H	6.822	-2.519	-14.976
H	6.081	-1.863	-13.520
C	5.007	-3.494	-14.356
N	4.041	-2.844	-15.117
C	3.009	-3.668	-15.221
H	2.098	-3.466	-15.763
N	3.247	-4.817	-14.557
H	2.617	-5.628	-14.514
C	4.506	-4.723	-14.000
H	4.909	-5.519	-13.391
CU	4.376	-1.090	-15.998
O	4.375	-2.487	-20.046
H	5.045	-1.783	-20.105
H	4.933	-3.291	-20.196
C	12.652	-0.418	-20.513
C	5.587	10.828	-20.538
C	-0.968	1.731	-24.273
C	-0.724	3.164	-20.743
C	-2.153	6.929	-15.532
C	10.437	-1.664	-17.166
C	7.551	3.958	-14.083
C	7.267	-3.610	-13.183

103

2NA

C	12.008	-0.639	-21.888
H	12.288	-1.628	-22.251
H	12.414	0.095	-22.583
C	10.503	-0.615	-21.786
C	9.735	0.550	-21.669
H	10.214	1.524	-21.743
C	8.368	0.474	-21.404
H	7.770	1.374	-21.303
C	7.745	-0.774	-21.257
O	6.428	-0.887	-20.917
H	5.977	-0.042	-21.185
C	8.487	-1.943	-21.420
H	7.986	-2.899	-21.311
C	9.851	-1.846	-21.671
H	10.474	-2.732	-21.725
C	4.712	9.905	-19.642
H	3.666	10.219	-19.728
H	5.022	10.016	-18.602
C	4.746	8.405	-19.958
O	4.525	7.567	-19.107
O	4.920	8.069	-21.246
H	4.782	7.101	-21.303
C	0.109	0.619	-24.405
H	0.358	0.490	-25.458
H	-0.286	-0.318	-24.018
H	0.999	0.890	-23.824
C	0.624	3.841	-20.215
H	0.574	3.880	-19.120
H	0.642	4.886	-20.538
C	1.892	3.138	-20.643
C	3.041	3.861	-21.133
C	1.994	1.760	-20.575
H	1.154	1.171	-20.212
C	4.194	3.245	-21.529
H	5.031	3.823	-21.904
C	3.146	1.080	-21.006
C	4.312	1.826	-21.500
O	3.029	5.247	-21.196
H	2.287	5.482	-21.772
N	3.203	-0.259	-20.997
H	4.027	-0.760	-21.296
H	2.445	-0.813	-20.625
O	5.348	1.226	-21.904
C	-0.761	7.642	-15.642
H	-0.436	7.926	-14.632
H	-0.910	8.568	-16.199
C	0.301	6.823	-16.328
C	0.903	5.720	-15.712
H	0.569	5.413	-14.724
C	1.933	5.019	-16.333
H	2.404	4.169	-15.849
C	2.383	5.434	-17.588
O	3.428	4.753	-18.166
H	3.759	5.303	-18.900
C	1.773	6.508	-18.237
H	2.128	6.808	-19.216
C	0.743	7.191	-17.603
H	0.280	8.038	-18.106
C	9.476	-1.107	-18.246
H	9.804	-1.489	-19.213
H	9.570	-0.020	-18.298
C	8.058	-1.467	-17.987
N	7.526	-2.724	-18.225
H	7.989	-3.487	-18.716
C	6.243	-2.728	-17.796
H	5.580	-3.571	-17.885
N	5.914	-1.543	-17.309
C	7.037	-0.750	-17.422
H	7.039	0.276	-17.084
C	6.010	3.787	-14.005
H	5.540	4.766	-14.160
H	5.748	3.473	-12.991
C	5.441	2.776	-14.951
N	4.901	3.050	-16.197
H	4.823	3.945	-16.673
C	4.360	1.909	-16.691
H	3.867	1.861	-17.651
N	4.515	0.910	-15.831
C	5.185	1.445	-14.746
H	5.426	0.840	-13.884
C	6.316	-2.795	-14.054
H	6.828	-2.516	-14.981
H	6.087	-1.860	-13.525
C	5.011	-3.487	-14.362
N	4.043	-2.834	-15.120
C	3.010	-3.659	-15.223
H	2.098	-3.455	-15.761
N	3.250	-4.809	-14.562
H	2.619	-5.620	-14.517
C	4.509	-4.717	-14.008
H	4.913	-5.514	-13.402
CU	4.372	-1.069	-15.988
O	4.351	-2.565	-19.997
H	5.035	-1.886	-19.857
H	4.921	-3.356	-20.196
C	12.655	-0.412	-20.515
C	5.590	10.822	-20.540
C	-0.960	1.730	-24.276
C	-0.677	3.188	-20.729
C	-2.121	6.933	-15.542
C	10.434	-1.663	-17.166
C	7.549	3.957	-14.076
C	7.270	-3.610	-13.189

103

TS(2NA,3NA)

C	12.007	-0.634	-21.899
H	12.230	-1.648	-22.236
H	12.474	0.055	-22.602
C	10.503	-0.504	-21.828
C	9.820	0.712	-21.686
H	10.362	1.652	-21.750
C	8.452	0.731	-21.406
H	7.918	1.670	-21.278
C	7.760	-0.474	-21.254
O	6.456	-0.523	-20.844
H	6.059	0.365	-20.775
C	8.395	-1.691	-21.493
H	7.826	-2.611	-21.403
C	9.756	-1.685	-21.773
H	10.296	-2.615	-21.899
C	4.710	9.906	-19.651
H	3.659	10.186	-19.775
H	4.988	10.040	-18.607
C	4.826	8.414	-19.963
O	4.702	7.554	-19.113
O	4.982	8.112	-21.260
H	5.028	7.140	-21.332
C	0.065	0.595	-24.428
H	0.313	0.476	-25.481
H	-0.344	-0.341	-24.050
H	0.958	0.848	-23.844
C	0.317	2.958	-19.710
H	0.205	2.093	-19.047

H	0.229	3.822	-19.040
C	1.657	2.930	-20.354
C	2.308	4.177	-20.625
C	2.283	1.743	-20.687
H	1.771	0.793	-20.552
C	3.603	4.259	-21.055
H	4.089	5.217	-21.190
C	3.562	1.795	-21.226
C	4.332	3.052	-21.275
O	1.641	5.321	-20.367
H	0.695	5.145	-20.262
N	4.244	0.713	-21.667
H	3.799	-0.195	-21.718
H	4.984	0.906	-22.332
O	5.554	2.974	-21.506
C	-0.810	7.663	-15.584
H	-0.535	7.987	-14.570
H	-0.972	8.569	-16.171
C	0.301	6.878	-16.201
C	0.870	5.767	-15.572
H	0.463	5.424	-14.624
C	1.958	5.109	-16.135
H	2.409	4.247	-15.656
C	2.490	5.593	-17.328
O	3.575	4.957	-17.868
H	3.932	5.549	-18.555
C	1.929	6.692	-17.977
H	2.351	7.037	-18.908
C	0.829	7.315	-17.416
H	0.383	8.169	-17.920
C	9.479	-1.115	-18.251
H	9.809	-1.501	-19.217
H	9.575	-0.027	-18.307
C	8.058	-1.469	-17.995
N	7.513	-2.716	-18.258
H	7.977	-3.485	-18.738
C	6.229	-2.713	-17.832
H	5.560	-3.552	-17.930
N	5.913	-1.538	-17.319
C	7.043	-0.753	-17.417
H	7.052	0.268	-17.064
C	6.013	3.788	-14.011
H	5.542	4.764	-14.181
H	5.753	3.489	-12.992
C	5.446	2.766	-14.944
N	4.890	3.038	-16.181
H	4.779	3.940	-16.646
C	4.353	1.894	-16.670
H	3.850	1.838	-17.624
N	4.528	0.894	-15.815
C	5.207	1.431	-14.737
H	5.467	0.825	-13.882
C	6.311	-2.798	-14.048
H	6.823	-2.519	-14.976

H	6.081	-1.863	-13.520
C	5.008	-3.493	-14.357
N	4.043	-2.844	-15.120
C	3.010	-3.668	-15.223
H	2.099	-3.467	-15.764
N	3.247	-4.815	-14.556
H	2.617	-5.626	-14.512
C	4.505	-4.721	-13.998
H	4.907	-5.515	-13.386
CU	4.376	-1.079	-15.986
O	4.434	-2.362	-20.172
H	5.146	-1.700	-20.231
H	4.940	-3.208	-20.236
C	12.648	-0.418	-20.519
C	5.587	10.829	-20.540
C	-0.987	1.721	-24.287
C	-0.873	2.949	-20.686
C	-2.137	6.903	-15.521
C	10.437	-1.665	-17.165
C	7.552	3.960	-14.087
C	7.267	-3.610	-13.182

103

3NA

C	11.957	-0.662	-21.879
H	12.126	-1.698	-22.186
H	12.445	-0.021	-22.614
C	10.459	-0.426	-21.803
C	9.892	0.848	-21.671
H	10.521	1.733	-21.714
C	8.524	1.001	-21.451
H	8.104	1.995	-21.329
C	7.688	-0.114	-21.361
O	6.340	-0.002	-21.095
H	6.132	0.906	-20.800
C	8.218	-1.390	-21.548
H	7.562	-2.252	-21.481
C	9.590	-1.524	-21.764
H	10.023	-2.512	-21.860
C	4.700	9.909	-19.665
H	3.648	10.173	-19.818
H	4.953	10.073	-18.619
C	4.840	8.400	-19.920
O	4.713	7.610	-18.996
O	4.990	8.008	-21.175
H	5.037	6.999	-21.194
C	0.058	0.582	-24.440
H	0.304	0.466	-25.494
H	-0.358	-0.353	-24.067
H	0.955	0.822	-23.857
C	0.304	2.540	-19.841
H	0.512	1.465	-19.859
H	0.046	2.766	-18.798
C	1.553	3.277	-20.269

C	1.652	4.713	-20.168
C	2.658	2.577	-20.761
H	2.594	1.494	-20.858
C	2.789	5.373	-20.546
H	2.858	6.445	-20.448
C	3.844	3.226	-21.131
C	3.948	4.685	-20.984
O	0.631	5.477	-19.711
H	0.068	4.979	-19.096
N	4.961	2.578	-21.612
H	4.810	1.737	-22.158
H	5.652	3.216	-22.007
O	5.039	5.268	-21.231
C	-0.844	7.706	-15.543
H	-0.626	8.050	-14.521
H	-0.994	8.598	-16.152
C	0.304	6.936	-16.084
C	0.841	5.812	-15.448
H	0.370	5.434	-14.544
C	1.983	5.195	-15.947
H	2.420	4.327	-15.462
C	2.592	5.742	-17.074
O	3.739	5.184	-17.560
H	4.118	5.848	-18.172
C	2.051	6.842	-17.729
H	2.550	7.251	-18.591
C	0.903	7.424	-17.239
H	0.478	8.285	-17.749
C	9.479	-1.121	-18.255
H	9.811	-1.511	-19.219
H	9.580	-0.034	-18.317
C	8.054	-1.468	-18.007
N	7.504	-2.711	-18.282
H	7.969	-3.484	-18.755
C	6.218	-2.705	-17.856
H	5.545	-3.539	-17.968
N	5.908	-1.534	-17.331
C	7.042	-0.753	-17.424
H	7.057	0.262	-17.051
C	6.016	3.790	-14.018
H	5.544	4.763	-14.203
H	5.755	3.505	-12.995
C	5.451	2.755	-14.937
N	4.878	3.026	-16.165
H	4.737	3.941	-16.605
C	4.344	1.880	-16.649
H	3.828	1.821	-17.596
N	4.540	0.878	-15.800
C	5.229	1.417	-14.730
H	5.506	0.810	-13.881
C	6.308	-2.799	-14.045
H	6.818	-2.520	-14.973
H	6.075	-1.865	-13.516
C	5.005	-3.498	-14.353

N	4.043	-2.854	-15.122
C	3.009	-3.678	-15.223
H	2.099	-3.479	-15.767
N	3.245	-4.822	-14.549
H	2.615	-5.633	-14.508
C	4.502	-4.725	-13.989
H	4.902	-5.516	-13.371
CU	4.379	-1.091	-15.978
O	4.578	-2.266	-20.252
H	5.216	-1.540	-20.362
H	5.123	-3.087	-20.308
C	12.638	-0.425	-20.520
C	5.582	10.828	-20.553
C	-0.981	1.718	-24.291
C	-0.946	2.793	-20.712
C	-2.154	6.921	-15.538
C	10.434	-1.667	-17.164
C	7.554	3.963	-14.097
C	7.267	-3.608	-13.180

103

4NA

C	12.058	-0.640	-21.910
H	12.281	-1.661	-22.224
H	12.560	0.032	-22.607
C	10.556	-0.502	-21.909
C	9.876	0.687	-21.611
H	10.429	1.612	-21.466
C	8.490	0.696	-21.483
H	7.961	1.614	-21.244
C	7.757	-0.484	-21.655
O	6.405	-0.507	-21.517
H	6.103	0.198	-20.881
C	8.411	-1.663	-22.012
H	7.827	-2.566	-22.163
C	9.795	-1.653	-22.138
H	10.327	-2.557	-22.416
C	4.709	9.902	-19.648
H	3.664	10.218	-19.733
H	5.017	10.005	-18.607
C	4.746	8.406	-19.981
O	4.502	7.553	-19.151
O	4.963	8.092	-21.268
H	4.889	7.120	-21.338
C	0.105	0.616	-24.409
H	0.346	0.483	-25.463
H	-0.290	-0.319	-24.016
H	1.000	0.884	-23.836
C	0.596	3.819	-20.186
H	0.417	4.101	-19.143
H	0.766	4.765	-20.712
C	1.796	2.909	-20.225
C	3.039	3.338	-20.814
C	1.747	1.674	-19.595
H	0.813	1.337	-19.149
C	4.180	2.582	-20.708
H	5.110	2.938	-21.138
C	2.885	0.859	-19.485
C	4.179	1.362	-19.966
O	3.154	4.565	-21.425
H	2.291	4.845	-21.771
N	2.882	-0.350	-18.875
H	3.676	-0.980	-19.031
H	1.990	-0.779	-18.665
O	5.222	0.706	-19.678
C	-0.761	7.640	-15.648
H	-0.435	7.934	-14.641
H	-0.909	8.563	-16.214
C	0.301	6.813	-16.326
C	0.891	5.711	-15.697
H	0.545	5.412	-14.710
C	1.923	5.001	-16.303
H	2.385	4.150	-15.812
C	2.387	5.406	-17.556
O	3.435	4.715	-18.112
H	3.767	5.229	-18.870
C	1.786	6.475	-18.221
H	2.153	6.772	-19.197
C	0.752	7.169	-17.600
H	0.297	8.014	-18.112
C	9.477	-1.118	-18.274
H	9.811	-1.511	-19.239
H	9.575	-0.032	-18.337
C	8.052	-1.469	-18.025
N	7.513	-2.720	-18.292
H	7.975	-3.482	-18.785
C	6.235	-2.729	-17.849
H	5.574	-3.574	-17.946
N	5.916	-1.560	-17.324
C	7.038	-0.764	-17.434
H	7.036	0.258	-17.087
C	6.016	3.789	-13.976
H	5.544	4.764	-14.146
H	5.767	3.495	-12.953
C	5.441	2.763	-14.894
N	4.856	3.040	-16.115
H	4.780	3.933	-16.597
C	4.307	1.902	-16.595
H	3.772	1.859	-17.526
N	4.503	0.898	-15.752
C	5.207	1.428	-14.687
H	5.488	0.818	-13.841
C	6.316	-2.795	-14.059
H	6.829	-2.516	-14.985
H	6.085	-1.860	-13.531
C	5.011	-3.488	-14.371
N	4.045	-2.842	-15.137
C	3.013	-3.668	-15.232
H	2.098	-3.470	-15.766
N	3.251	-4.813	-14.561
H	2.621	-5.624	-14.512
C	4.510	-4.715	-14.006
H	4.913	-5.507	-13.391
CU	4.369	-1.065	-16.026
O	4.571	-2.297	-20.241
H	5.240	-1.677	-20.601
H	5.059	-3.157	-20.241
C	12.658	-0.413	-20.515
C	5.589	10.823	-20.540
C	-0.961	1.730	-24.276
C	-0.690	3.182	-20.731
C	-2.121	6.933	-15.543
C	10.429	-1.662	-17.174
C	7.553	3.958	-14.073
C	7.270	-3.610	-13.190

103

TS(4NA,5NA)

C	12.017	-0.646	-21.899
H	12.220	-1.671	-22.213
H	12.514	0.017	-22.609
C	10.516	-0.468	-21.867
C	9.881	0.761	-21.642
H	10.464	1.675	-21.578
C	8.499	0.822	-21.475
H	8.009	1.776	-21.299
C	7.728	-0.341	-21.529
O	6.369	-0.316	-21.321
H	6.142	0.464	-20.792
C	8.329	-1.565	-21.811
H	7.717	-2.461	-21.861
C	9.710	-1.606	-21.979
H	10.208	-2.546	-22.181
C	4.703	9.908	-19.651
H	3.656	10.216	-19.745
H	5.003	10.015	-18.609
C	4.760	8.416	-19.987
O	4.529	7.551	-19.164
O	4.988	8.116	-21.274
H	4.958	7.143	-21.351
C	0.075	0.607	-24.410
H	0.321	0.483	-25.463
H	-0.337	-0.326	-24.029
H	0.970	0.853	-23.827
C	0.609	3.805	-20.182
H	0.366	4.373	-19.278
H	0.997	4.549	-20.892
C	1.569	2.720	-19.811
C	2.765	2.457	-20.548
C	1.202	1.876	-18.771
H	0.278	2.076	-18.237
C	3.584	1.418	-20.190

H	4.499	1.256	-20.743
C	1.945	0.742	-18.456
C	3.251	0.537	-19.106
O	3.134	3.238	-21.612
H	2.436	3.886	-21.793
N	1.568	-0.144	-17.503
H	2.111	-0.994	-17.424
H	0.616	-0.158	-17.164
O	4.038	-0.323	-18.642
C	-0.761	7.633	-15.645
H	-0.423	7.895	-14.634
H	-0.911	8.572	-16.181
C	0.288	6.826	-16.363
C	0.873	5.688	-15.794
H	0.530	5.344	-14.821
C	1.901	5.004	-16.436
H	2.362	4.129	-15.990
C	2.369	5.473	-17.665
O	3.422	4.819	-18.256
H	3.822	5.440	-18.892
C	1.765	6.573	-18.275
H	2.125	6.917	-19.239
C	0.737	7.240	-17.620
H	0.284	8.110	-18.091
C	9.513	-1.114	-18.279
H	9.861	-1.504	-19.239
H	9.609	-0.027	-18.335
C	8.093	-1.471	-18.039
N	7.545	-2.711	-18.326
H	7.995	-3.468	-18.837
C	6.297	-2.749	-17.797
H	5.620	-3.579	-17.909
N	6.022	-1.612	-17.194
C	7.117	-0.799	-17.354
H	7.131	0.209	-16.964
C	6.032	3.809	-13.959
H	5.565	4.790	-14.115
H	5.790	3.505	-12.937
C	5.454	2.805	-14.884
N	4.908	3.116	-16.112
H	4.854	4.022	-16.567
C	4.378	1.991	-16.639
H	3.885	1.998	-17.590
N	4.545	0.963	-15.818
C	5.216	1.465	-14.720
H	5.480	0.831	-13.887
C	6.312	-2.782	-14.043
H	6.827	-2.497	-14.965
H	6.083	-1.851	-13.508
C	5.004	-3.465	-14.372
N	4.043	-2.815	-15.147
C	3.019	-3.648	-15.257
H	2.111	-3.458	-15.808
N	3.250	-4.798	-14.589

H	2.621	-5.611	-14.545
C	4.502	-4.696	-14.019
H	4.902	-5.488	-13.404
CU	4.379	-1.026	-16.104
O	4.570	-2.374	-20.273
H	5.192	-1.646	-20.434
H	5.130	-3.189	-20.326
C	12.648	-0.418	-20.516
C	5.584	10.830	-20.540
C	-0.974	1.736	-24.269
C	-0.687	3.202	-20.736
C	-2.123	6.930	-15.545
C	10.445	-1.661	-17.166
C	7.565	3.964	-14.077
C	7.266	-3.605	-13.180

103

5NA

C	11.973	-0.654	-21.880
H	12.155	-1.685	-22.194
H	12.460	-0.002	-22.607
C	10.472	-0.441	-21.814
C	9.876	0.819	-21.668
H	10.488	1.717	-21.677
C	8.499	0.940	-21.480
H	8.055	1.923	-21.347
C	7.684	-0.194	-21.450
O	6.325	-0.133	-21.248
H	6.034	0.780	-21.100
C	8.249	-1.456	-21.629
H	7.609	-2.333	-21.604
C	9.627	-1.557	-21.806
H	10.086	-2.532	-21.910
C	4.700	9.915	-19.671
H	3.652	10.215	-19.771
H	4.995	10.031	-18.627
C	4.774	8.424	-19.996
O	4.556	7.561	-19.166
O	5.006	8.119	-21.280
H	4.990	7.147	-21.356
C	0.041	0.601	-24.409
H	0.298	0.487	-25.461
H	-0.389	-0.331	-24.044
H	0.931	0.824	-23.808
C	0.709	3.562	-20.136
H	0.550	4.361	-19.407
H	1.332	4.013	-20.925
C	1.353	2.389	-19.421
C	2.288	1.481	-20.024
C	0.873	2.079	-18.158
H	0.161	2.752	-17.691
C	2.692	0.323	-19.387
H	3.380	-0.368	-19.860

C	1.183	0.872	-17.522
C	2.149	-0.040	-18.117
O	2.810	1.739	-21.271
H	2.514	2.620	-21.546
N	0.586	0.506	-16.350
H	0.728	-0.470	-16.102
H	-0.368	0.836	-16.247
O	2.456	-1.110	-17.476
C	-0.760	7.625	-15.644
H	-0.408	7.854	-14.629
H	-0.911	8.581	-16.149
C	0.277	6.839	-16.403
C	0.857	5.671	-15.893
H	0.515	5.280	-14.938
C	1.882	5.016	-16.570
H	2.338	4.116	-16.171
C	2.352	5.546	-17.774
O	3.411	4.932	-18.400
H	3.856	5.626	-18.924
C	1.744	6.672	-18.332
H	2.097	7.057	-19.283
C	0.720	7.311	-17.642
H	0.269	8.203	-18.069
C	9.503	-1.110	-18.253
H	9.841	-1.501	-19.215
H	9.605	-0.023	-18.311
C	8.078	-1.455	-18.011
N	7.522	-2.691	-18.302
H	7.983	-3.464	-18.779
C	6.240	-2.686	-17.862
H	5.557	-3.510	-17.982
N	5.943	-1.524	-17.316
C	7.076	-0.746	-17.405
H	7.098	0.262	-17.015
C	6.032	3.797	-14.044
H	5.563	4.778	-14.187
H	5.765	3.469	-13.035
C	5.469	2.804	-15.012
N	4.915	3.125	-16.241
H	4.853	4.037	-16.682
C	4.378	1.999	-16.776
H	3.882	1.977	-17.734
N	4.546	0.970	-15.960
C	5.224	1.461	-14.863
H	5.479	0.821	-14.031
C	6.313	-2.777	-14.039
H	6.833	-2.486	-14.957
H	6.080	-1.848	-13.500
C	5.005	-3.458	-14.381
N	4.062	-2.810	-15.174
C	3.032	-3.633	-15.298
H	2.148	-3.421	-15.880
N	3.243	-4.781	-14.618
H	2.610	-5.590	-14.575

C	4.490	-4.685	-14.028
H	4.875	-5.479	-13.405
CU	4.172	-1.052	-16.321
O	4.626	-2.278	-20.289
H	5.289	-1.589	-20.467
H	5.134	-3.126	-20.321
C	12.639	-0.422	-20.514
C	5.579	10.835	-20.563
C	-0.994	1.742	-24.265
C	-0.664	3.168	-20.718
C	-2.124	6.928	-15.549
C	10.444	-1.661	-17.153
C	7.571	3.969	-14.103
C	7.264	-3.602	-13.171

110

TS(1,6)

C	12.056	-0.631	-21.911
H	12.303	-1.638	-22.243
H	12.535	0.067	-22.599
C	10.552	-0.546	-21.898
C	9.815	0.620	-21.656
H	10.324	1.576	-21.559
C	8.428	0.558	-21.503
H	7.860	1.457	-21.271
C	7.769	-0.674	-21.597
O	6.422	-0.804	-21.415
H	6.038	0.063	-21.155
C	8.481	-1.832	-21.891
H	7.938	-2.768	-21.975
C	9.856	-1.750	-22.041
H	10.443	-2.635	-22.251
C	4.709	9.915	-19.640
H	3.663	10.226	-19.730
H	5.015	10.025	-18.600
C	4.770	8.426	-19.972
O	4.552	7.550	-19.161
O	4.998	8.127	-21.267
H	5.001	7.154	-21.321
C	0.075	0.596	-24.457
H	0.234	0.462	-25.526
H	-0.344	-0.333	-24.069
C	1.429	0.933	-23.828
O	2.130	1.882	-24.215
N	1.816	0.101	-22.848
H	1.165	-0.578	-22.452
H	2.683	0.286	-22.367
C	0.495	3.853	-20.090
H	0.284	4.007	-19.027
H	0.627	4.835	-20.553
C	1.732	3.054	-20.245
C	1.896	1.832	-19.503
C	2.707	3.457	-21.124
H	2.538	4.338	-21.740
C	3.026	1.063	-19.659
H	3.174	0.119	-19.136
C	3.917	2.759	-21.219
C	4.140	1.579	-20.380
O	0.899	1.533	-18.636
H	0.852	0.581	-18.462
N	4.920	3.172	-21.999
H	5.775	2.632	-22.060
H	4.799	3.905	-22.686
O	5.296	1.085	-20.267
C	-0.758	7.622	-15.634
H	-0.440	7.905	-14.627
H	-0.883	8.543	-16.204
C	0.320	6.793	-16.285
C	0.923	5.724	-15.602
H	0.585	5.466	-14.604
C	1.962	4.997	-16.194
H	2.430	4.174	-15.670
C	2.410	5.353	-17.474
O	3.449	4.663	-18.035
H	3.744	5.143	-18.819
C	1.800	6.404	-18.173
H	2.149	6.669	-19.162
C	0.756	7.115	-17.577
H	0.288	7.933	-18.114
C	9.482	-1.118	-18.267
H	9.817	-1.507	-19.233
H	9.579	-0.031	-18.326
C	8.058	-1.470	-18.020
N	7.511	-2.713	-18.295
H	7.969	-3.478	-18.788
C	6.232	-2.717	-17.852
H	5.560	-3.552	-17.960
N	5.923	-1.548	-17.318
C	7.051	-0.762	-17.421
H	7.066	0.253	-17.051
C	6.015	3.788	-13.980
H	5.540	4.761	-14.159
H	5.767	3.503	-12.954
C	5.450	2.751	-14.887
N	5.015	2.984	-16.172
H	4.978	3.871	-16.663
C	4.496	1.838	-16.665
H	4.092	1.754	-17.654
N	4.561	0.872	-15.765
C	5.154	1.433	-14.653
H	5.323	0.855	-13.761
C	6.311	-2.799	-14.049
H	6.819	-2.522	-14.978
H	6.080	-1.864	-13.520
C	5.007	-3.498	-14.353
N	4.043	-2.856	-15.122
C	3.011	-3.683	-15.221
H	2.102	-3.490	-15.767
N	3.250	-4.824	-14.546
H	2.621	-5.636	-14.500
C	4.506	-4.724	-13.986
H	4.907	-5.515	-13.370
CU	4.399	-1.095	-15.980
O	4.425	-2.335	-20.071
H	5.104	-1.701	-20.388
H	4.917	-3.183	-20.207
O	3.338	3.245	-26.285
H	3.142	4.185	-26.108
H	2.812	2.756	-25.620
H	12.480	-0.478	-20.921
H	5.332	10.563	-20.275
H	-0.657	1.401	-24.327
H	-0.369	3.369	-20.533
H	-1.725	7.132	-15.569
H	10.155	-1.505	-17.489
H	7.104	3.911	-14.052
H	6.990	-3.373	-13.436

110

6

C	12.079	-0.642	-21.913
H	12.283	-1.671	-22.213
H	12.606	0.013	-22.609
C	10.583	-0.466	-21.937
C	9.934	0.740	-21.639
H	10.509	1.644	-21.464
C	8.546	0.791	-21.560
H	8.033	1.717	-21.322
C	7.788	-0.357	-21.802
O	6.422	-0.322	-21.781
H	6.042	-0.069	-20.888
C	8.415	-1.555	-22.136
H	7.805	-2.433	-22.326
C	9.802	-1.594	-22.201
H	10.311	-2.515	-22.465
C	4.717	9.898	-19.642
H	3.670	10.211	-19.719
H	5.030	9.995	-18.602
C	4.763	8.408	-19.992
O	4.536	7.534	-19.179
O	4.980	8.122	-21.287
H	4.937	7.151	-21.371
C	0.091	0.593	-24.460
H	0.262	0.466	-25.529
H	-0.331	-0.339	-24.083
C	1.438	0.916	-23.816
O	2.149	1.870	-24.184
N	1.821	0.066	-22.854
H	1.163	-0.607	-22.460
H	2.695	0.226	-22.373
C	0.613	3.804	-20.204

H	0.423	4.160	-19.189
H	0.865	4.672	-20.823
C	1.757	2.836	-20.190
C	1.797	1.815	-19.172
C	2.746	2.847	-21.146
H	2.682	3.553	-21.970
C	2.794	0.863	-19.149
H	2.836	0.104	-18.372
C	3.811	1.916	-21.134
C	3.913	0.954	-20.022
O	0.825	1.905	-18.246
H	0.728	1.086	-17.733
N	4.698	1.873	-22.153
H	5.413	1.141	-22.203
H	4.471	2.353	-23.016
O	4.955	0.248	-19.819
C	-0.753	7.629	-15.648
H	-0.431	7.931	-14.642
H	-0.891	8.546	-16.225
C	0.309	6.786	-16.309
C	0.886	5.681	-15.670
H	0.532	5.392	-14.683
C	1.913	4.954	-16.265
H	2.361	4.099	-15.769
C	2.387	5.346	-17.518
O	3.432	4.638	-18.072
H	3.769	5.158	-18.821
C	1.807	6.422	-18.188
H	2.179	6.713	-19.165
C	0.777	7.130	-17.580
H	0.335	7.976	-18.103
C	9.482	-1.121	-18.280
H	9.820	-1.517	-19.243
H	9.582	-0.035	-18.344
C	8.058	-1.471	-18.032
N	7.511	-2.717	-18.299
H	7.968	-3.486	-18.788
C	6.238	-2.725	-17.840
H	5.566	-3.560	-17.945
N	5.931	-1.555	-17.310
C	7.053	-0.764	-17.426
H	7.063	0.253	-17.063
C	6.017	3.787	-13.939
H	5.539	4.763	-14.091
H	5.788	3.482	-12.915
C	5.440	2.769	-14.855
N	4.951	3.045	-16.116
H	4.873	3.943	-16.584
C	4.432	1.910	-16.631
H	3.981	1.867	-17.605
N	4.549	0.908	-15.770
C	5.176	1.438	-14.656
H	5.391	0.831	-13.789
C	6.315	-2.794	-14.055
H	6.828	-2.511	-14.980
H	6.085	-1.861	-13.522
C	5.011	-3.485	-14.370
N	4.049	-2.835	-15.139
C	3.016	-3.660	-15.245
H	2.109	-3.462	-15.792
N	3.250	-4.807	-14.576
H	2.617	-5.617	-14.529
C	4.505	-4.713	-14.013
H	4.903	-5.507	-13.398
CU	4.395	-1.060	-16.003
O	4.218	-2.465	-19.990
H	4.657	-1.621	-19.776
H	4.936	-3.117	-20.146
O	3.341	3.253	-26.276
H	3.138	4.193	-26.097
H	2.794	2.753	-25.636
C	12.661	-0.413	-20.513
C	5.591	10.823	-20.535
C	-0.948	1.723	-24.281
C	-0.689	3.179	-20.732
C	-2.118	6.934	-15.542
C	10.429	-1.663	-17.176
C	7.551	3.959	-14.068
C	7.270	-3.609	-13.190

110

TS(6,5)

C	12.007	-0.636	-21.899
H	12.226	-1.654	-22.227
H	12.491	0.041	-22.604
C	10.499	-0.490	-21.863
C	9.824	0.707	-21.589
H	10.378	1.636	-21.488
C	8.439	0.723	-21.415
H	7.926	1.659	-21.210
C	7.697	-0.460	-21.492
O	6.349	-0.512	-21.239
H	6.069	0.303	-20.793
C	8.339	-1.647	-21.853
H	7.756	-2.558	-21.947
C	9.719	-1.640	-22.038
H	10.239	-2.552	-22.308
C	4.711	9.912	-19.637
H	3.665	10.221	-19.726
H	5.019	10.023	-18.597
C	4.769	8.421	-19.964
O	4.557	7.563	-19.128
O	4.976	8.110	-21.252
H	4.937	7.136	-21.322
C	-0.143	0.478	-24.539
H	-0.200	0.245	-25.597
H	-0.589	-0.365	-24.010
C	1.326	0.626	-24.229

O	1.987	1.663	-24.332
N	1.876	-0.569	-23.938
H	1.311	-1.393	-23.776
H	2.870	-0.631	-23.775
C	0.678	3.756	-20.244
H	0.500	4.196	-19.252
H	1.004	4.573	-20.903
C	1.729	2.679	-20.094
C	2.921	2.914	-19.314
C	1.514	1.382	-20.477
H	0.658	1.049	-21.053
C	3.759	1.919	-18.853
H	4.606	2.144	-18.217
C	2.175	0.369	-19.797
C	3.377	0.561	-19.004
O	3.240	4.196	-19.034
H	2.418	4.710	-18.968
N	1.500	-0.762	-19.611
H	2.081	-1.546	-19.315
H	0.792	-0.984	-20.317
O	3.920	-0.433	-18.448
C	-0.861	7.749	-15.610
H	-0.642	8.169	-14.619
H	-1.025	8.589	-16.285
C	0.299	6.949	-16.069
C	1.001	6.167	-15.149
H	0.617	6.046	-14.139
C	2.217	5.617	-15.491
H	2.824	5.072	-14.777
C	2.739	5.881	-16.754
O	4.017	5.491	-16.952
H	4.313	5.930	-17.767
C	1.982	6.525	-17.742
H	2.392	6.706	-18.731
C	0.756	7.069	-17.377
H	0.176	7.643	-18.097
C	9.509	-1.113	-18.281
H	9.836	-1.525	-19.239
H	9.627	-0.029	-18.354
C	8.087	-1.437	-18.012
N	7.501	-2.654	-18.316
H	7.925	-3.411	-18.848
C	6.269	-2.676	-17.747
H	5.561	-3.480	-17.859
N	6.039	-1.546	-17.109
C	7.150	-0.759	-17.277
H	7.213	0.233	-16.849
C	6.008	3.838	-14.000
H	5.575	4.770	-14.382
H	5.710	3.769	-12.949
C	5.391	2.674	-14.715
N	4.264	2.886	-15.495
H	4.057	3.756	-15.979
C	3.685	1.689	-15.745

H	2.793	1.558	-16.335
N	4.379	0.711	-15.188
C	5.441	1.313	-14.534
H	6.148	0.728	-13.963
C	6.301	-2.814	-14.036
H	6.806	-2.522	-14.960
H	6.051	-1.887	-13.502
C	5.013	-3.538	-14.345
N	4.053	-2.939	-15.150
C	3.025	-3.769	-15.213
H	2.112	-3.598	-15.761
N	3.263	-4.876	-14.483
H	2.639	-5.688	-14.421
C	4.517	-4.751	-13.925
H	4.918	-5.512	-13.272
CU	4.390	-1.123	-15.963
O	4.279	-2.610	-20.066
H	4.731	-1.827	-19.719
H	4.978	-3.299	-20.205
O	3.338	3.221	-26.292
H	3.147	4.156	-26.080
H	2.849	2.710	-25.619
C	12.647	-0.415	-20.517
C	5.589	10.828	-20.534
C	-0.994	1.728	-24.285
C	-0.673	3.203	-20.739
C	-2.149	6.936	-15.538
C	10.443	-1.661	-17.171
C	7.551	3.960	-14.077
C	7.270	-3.613	-13.177

110

TS(6,7)

C	12.085	-0.654	-21.916
H	12.260	-1.693	-22.198
H	12.632	-0.024	-22.620
C	10.601	-0.428	-21.947
C	10.016	0.817	-21.682
H	10.629	1.704	-21.559
C	8.641	0.922	-21.538
H	8.171	1.877	-21.315
C	7.849	-0.217	-21.666
O	6.509	-0.163	-21.433
H	6.313	0.579	-20.840
C	8.391	-1.445	-22.018
H	7.729	-2.299	-22.125
C	9.769	-1.534	-22.150
H	10.231	-2.485	-22.388
C	4.707	9.915	-19.651
H	3.662	10.229	-19.740
H	5.013	10.024	-18.611
C	4.758	8.424	-19.983
O	4.530	7.559	-19.163
O	4.986	8.125	-21.275
H	4.938	7.152	-21.353
C	0.074	0.598	-24.458
H	0.245	0.473	-25.526
H	-0.350	-0.336	-24.084
C	1.419	0.921	-23.806
O	2.093	1.916	-24.117
N	1.837	0.024	-22.904
H	1.189	-0.667	-22.529
H	2.698	0.195	-22.405
C	0.595	3.815	-20.152
H	0.335	4.326	-19.223
H	0.982	4.552	-20.867
C	1.614	2.770	-19.863
C	1.538	2.014	-18.642
C	2.555	2.435	-20.812
H	2.611	2.988	-21.747
C	2.258	0.865	-18.466
H	2.210	0.309	-17.534
C	3.373	1.325	-20.625
C	3.319	0.553	-19.378
O	0.816	2.559	-17.618
H	0.001	2.069	-17.500
N	4.238	0.918	-21.581
H	4.683	0.009	-21.514
H	4.397	1.466	-22.416
O	4.272	-0.202	-19.062
C	-0.756	7.610	-15.622
H	-0.437	7.873	-14.613
H	-0.865	8.535	-16.183
C	0.333	6.787	-16.267
C	0.948	5.742	-15.550
H	0.622	5.511	-14.544
C	1.985	5.001	-16.145
H	2.457	4.194	-15.607
C	2.413	5.325	-17.450
O	3.428	4.637	-18.029
H	3.683	5.074	-18.847
C	1.788	6.367	-18.168
H	2.112	6.604	-19.169
C	0.742	7.091	-17.577
H	0.260	7.889	-18.123
C	9.496	-1.124	-18.280
H	9.845	-1.510	-19.243
H	9.583	-0.035	-18.335
C	8.075	-1.491	-18.041
N	7.543	-2.744	-18.299
H	8.009	-3.513	-18.778
C	6.281	-2.773	-17.808
H	5.616	-3.616	-17.902
N	5.972	-1.613	-17.265
C	7.072	-0.801	-17.414
H	7.074	0.218	-17.054
C	6.023	3.781	-13.947
H	5.541	4.752	-14.119
H	5.787	3.489	-12.921
C	5.464	2.747	-14.852
N	5.086	2.993	-16.155
H	5.058	3.889	-16.629
C	4.590	1.852	-16.678
H	4.223	1.786	-17.681
N	4.610	0.878	-15.783
C	5.157	1.428	-14.638
H	5.296	0.837	-13.745
C	6.309	-2.793	-14.044
H	6.821	-2.511	-14.970
H	6.079	-1.860	-13.513
C	5.005	-3.488	-14.359
N	4.042	-2.850	-15.138
C	3.014	-3.681	-15.237
H	2.105	-3.492	-15.787
N	3.250	-4.820	-14.555
H	2.624	-5.634	-14.509
C	4.504	-4.714	-13.991
H	4.905	-5.501	-13.369
CU	4.386	-1.086	-16.033
O	4.622	-2.366	-20.353
H	5.063	-1.677	-19.836
H	5.216	-3.160	-20.368
O	3.303	3.265	-26.240
H	3.122	4.216	-26.092
H	2.795	2.808	-25.543
H	12.492	-0.486	-20.922
H	5.331	10.563	-20.286
H	-0.661	1.401	-24.329
H	-0.315	3.374	-20.560
H	-1.725	7.129	-15.564
H	10.163	-1.505	-17.493
H	7.110	3.908	-14.034
H	6.989	-3.371	-13.433

110

7

C	11.982	-0.673	-21.882
H	12.121	-1.722	-22.159
H	12.512	-0.069	-22.623
C	10.497	-0.374	-21.843
C	10.002	0.927	-21.701
H	10.676	1.778	-21.718
C	8.645	1.151	-21.498
H	8.286	2.164	-21.351
C	7.740	0.092	-21.470
O	6.396	0.295	-21.240
H	6.248	1.244	-21.102
C	8.198	-1.210	-21.668
H	7.490	-2.032	-21.663
C	9.566	-1.421	-21.843
H	9.935	-2.434	-21.960
C	4.695	9.924	-19.662

H 3.651 10.240 -19.747
H 5.004 10.034 -18.622
C 4.735 8.430 -19.988
O 4.458 7.577 -19.165
O 5.008 8.116 -21.262
H 4.946 7.146 -21.342
C -0.003 0.568 -24.469
H 0.123 0.418 -25.540
H -0.440 -0.343 -24.059
C 1.381 0.861 -23.894
O 2.081 1.813 -24.270
N 1.816 -0.023 -22.979
H 1.176 -0.674 -22.523
H 2.727 0.123 -22.571
C 0.669 3.609 -20.083
H 0.500 4.513 -19.487
H 1.364 3.905 -20.878
C 1.267 2.492 -19.258
C 0.912 2.260 -17.876
C 2.056 1.547 -19.893
H 2.334 1.712 -20.931
C 1.255 1.085 -17.236
H 0.940 0.911 -16.215
C 2.432 0.355 -19.261
C 2.004 0.074 -17.887
O 0.269 3.188 -17.129
H -0.055 3.926 -17.666
N 3.156 -0.608 -19.877
H 3.515 -1.347 -19.283
H 3.781 -0.380 -20.637
O 2.378 -1.020 -17.339
C -0.789 7.676 -15.621
H -0.485 7.962 -14.606
H -0.957 8.600 -16.175
C 0.306 6.903 -16.297
C 1.057 5.940 -15.616
H 0.808 5.697 -14.586
C 2.124 5.295 -16.232
H 2.698 4.537 -15.710
C 2.445 5.617 -17.549
O 3.504 4.982 -18.153
H 3.883 5.632 -18.775
C 1.692 6.555 -18.257
H 1.958 6.790 -19.283
C 0.632 7.194 -17.626
H 0.055 7.939 -18.169
C 9.485 -1.121 -18.253
H 9.831 -1.502 -19.218
H 9.569 -0.032 -18.307
C 8.057 -1.487 -18.028
N 7.534 -2.753 -18.253
H 8.022 -3.544 -18.669
C 6.229 -2.740 -17.881
H 5.574 -3.593 -17.961

N 5.877 -1.543 -17.452
C 7.006 -0.755 -17.537
H 6.992 0.278 -17.220
C 6.035 3.804 -14.037
H 5.568 4.785 -14.188
H 5.768 3.483 -13.027
C 5.484 2.803 -14.998
N 4.961 3.105 -16.244
H 4.869 4.016 -16.686
C 4.456 1.966 -16.781
H 3.962 1.939 -17.739
N 4.623 0.944 -15.953
C 5.261 1.458 -14.843
H 5.508 0.826 -14.000
C 6.317 -2.783 -14.040
H 6.839 -2.492 -14.958
H 6.081 -1.854 -13.503
C 5.012 -3.466 -14.378
N 4.069 -2.808 -15.160
C 3.027 -3.618 -15.275
H 2.133 -3.385 -15.835
N 3.236 -4.771 -14.603
H 2.596 -5.575 -14.556
C 4.489 -4.690 -14.024
H 4.871 -5.488 -13.404
CU 4.185 -1.049 -16.234
O 5.109 -2.150 -20.611
H 5.776 -1.550 -20.247
H 5.470 -3.071 -20.499
O 3.358 3.186 -26.332
H 3.158 4.115 -26.110
H 2.833 2.668 -25.688
C 12.640 -0.425 -20.515
C 5.578 10.834 -20.561
C -0.991 1.737 -24.270
C -0.672 3.173 -20.710
C -2.128 6.930 -15.546
C 10.439 -1.661 -17.155
C 7.573 3.971 -14.102
C 7.267 -3.604 -13.170

97

OX
C 11.993 -0.666 -21.882
H 12.217 -1.684 -22.207
H 12.446 0.017 -22.600
C 10.492 -0.535 -21.795
C 9.828 0.693 -21.677
H 10.391 1.622 -21.732
C 8.455 0.741 -21.456
H 7.945 1.692 -21.356
C 7.711 -0.445 -21.367
O 6.368 -0.419 -21.127
H 6.015 0.520 -21.309

C 8.350 -1.677 -21.513
H 7.764 -2.591 -21.462
C 9.728 -1.702 -21.716
H 10.261 -2.642 -21.782
C 4.690 9.916 -19.663
H 3.645 10.222 -19.775
H 4.977 10.045 -18.619
C 4.748 8.406 -19.949
O 4.494 7.592 -19.084
O 4.993 8.042 -21.213
H 4.953 7.059 -21.254
C 0.053 0.593 -24.448
H 0.208 0.451 -25.516
H -0.366 -0.330 -24.046
C 1.407 0.949 -23.827
O 2.140 1.819 -24.313
N 1.741 0.261 -22.717
H 1.080 -0.385 -22.289
H 2.573 0.537 -22.207
C 0.503 3.743 -19.969
H 0.373 3.581 -18.892
H 0.497 4.821 -20.146
C 1.849 3.216 -20.375
C 2.419 1.919 -19.808
C 2.647 4.031 -21.099
H 2.298 4.996 -21.454
C 3.692 1.514 -20.281
H 4.064 0.539 -19.979
C 4.083 3.779 -21.238
C 4.529 2.326 -21.072
O 1.776 1.248 -18.948
O 4.884 4.699 -21.336
O 5.621 1.975 -21.570
C 9.452 -1.127 -18.239
H 9.757 -1.525 -19.209
H 9.551 -0.041 -18.313
C 8.032 -1.475 -17.950
N 7.464 -2.707 -18.242
H 7.915 -3.462 -18.759
C 6.194 -2.715 -17.797
H 5.509 -3.536 -17.913
N 5.912 -1.549 -17.230
C 7.046 -0.762 -17.325
H 7.076 0.245 -16.944
C 6.023 3.733 -14.042
H 5.521 4.696 -14.191
H 5.764 3.392 -13.036
C 5.519 2.723 -15.025
N 5.063 3.036 -16.292
H 4.985 3.959 -16.713
C 4.613 1.915 -16.893
H 4.211 1.887 -17.897
N 4.756 0.881 -16.074
C 5.316 1.369 -14.908

H	5.529	0.722	-14.070
C	6.318	-2.769	-14.070
H	6.865	-2.463	-14.967
H	6.069	-1.849	-13.524
C	5.019	-3.451	-14.457
N	4.096	-2.850	-15.325
C	3.065	-3.682	-15.425
H	2.190	-3.522	-16.037
N	3.252	-4.780	-14.670
H	2.616	-5.589	-14.602
C	4.479	-4.649	-14.051
H	4.842	-5.404	-13.368
CU	4.147	-1.044	-16.490
O	4.612	-2.264	-20.268
H	5.278	-1.608	-20.600
H	5.086	-3.133	-20.299
O	3.304	3.170	-26.238
H	3.162	4.087	-25.938
H	2.942	2.635	-25.487
O	2.226	-0.337	-15.910
H	1.990	0.328	-16.592
H	1.524	-1.010	-15.980
O	3.066	-1.266	-18.326
H	3.630	-1.624	-19.067
H	2.610	-0.444	-18.652
C	12.646	-0.425	-20.515
C	5.580	10.827	-20.553
C	-0.970	1.737	-24.265
C	-0.721	3.140	-20.694
C	10.430	-1.667	-17.164
C	7.555	3.957	-14.098
C	7.257	-3.600	-13.189

64

TS(OX, OXrot)

C	11.990	-0.652	-21.888
H	12.234	-1.659	-22.229
H	12.434	0.051	-22.594
C	10.482	-0.564	-21.818
C	9.762	0.625	-21.653
H	10.282	1.581	-21.649
C	8.383	0.604	-21.457
H	7.838	1.527	-21.308
C	7.684	-0.617	-21.425
O	6.340	-0.690	-21.194
H	5.967	0.233	-21.318
C	8.385	-1.807	-21.629
H	7.838	-2.745	-21.617
C	9.762	-1.760	-21.821
H	10.339	-2.669	-21.939
C	4.691	9.939	-19.646
H	3.646	10.243	-19.762
H	4.981	10.075	-18.605
C	4.759	8.437	-19.928
O	4.560	7.614	-19.065
O	4.959	8.084	-21.212
H	4.889	7.109	-21.235
C	-0.223	0.434	-24.531
H	-0.289	0.210	-25.587
H	-0.691	-0.387	-23.991
C	1.244	0.591	-24.259
O	1.890	1.451	-24.857
N	1.778	-0.272	-23.378
H	1.166	-0.831	-22.788
H	2.745	-0.113	-23.125
C	0.556	3.867	-20.278
H	0.588	3.869	-19.187
H	0.497	4.925	-20.560
C	1.870	3.323	-20.804
C	2.963	3.782	-19.908
C	2.259	2.651	-21.912
H	1.658	2.370	-22.761
C	4.225	3.185	-19.920
H	4.815	3.502	-19.081
C	3.630	2.142	-22.062
C	4.634	2.345	-20.944
O	2.678	4.703	-19.119
O	3.977	1.470	-23.023
O	5.716	1.726	-21.057
C	-0.881	7.789	-15.508
H	-0.753	8.223	-14.504
H	-1.033	8.621	-16.197
C	0.353	7.065	-15.888
C	1.106	6.342	-14.963
H	0.750	6.250	-13.938
C	2.308	5.755	-15.345
H	2.915	5.173	-14.657
C	2.760	5.929	-16.650
O	3.923	5.360	-17.037
H	3.785	5.228	-18.011
C	2.015	6.676	-17.576
H	2.389	6.814	-18.577
C	0.811	7.215	-17.195
H	0.219	7.787	-17.908
C	12.641	-0.424	-20.515
C	5.582	10.834	-20.548
C	-1.017	1.729	-24.275
C	-0.723	3.158	-20.735
C	-2.148	6.930	-15.533

97

OXrot

C	11.957	-0.650	-21.874
H	12.222	-1.642	-22.242
H	12.352	0.080	-22.580
C	10.448	-0.606	-21.747
C	9.690	0.569	-21.647
H	10.179	1.537	-21.732
C	8.317	0.517	-21.402
H	7.728	1.426	-21.329
C	7.665	-0.719	-21.257
O	6.343	-0.807	-20.942
H	5.852	0.029	-21.231
C	8.401	-1.898	-21.400
H	7.889	-2.851	-21.316
C	9.773	-1.823	-21.629
H	10.379	-2.721	-21.673
C	4.697	9.930	-19.644
H	3.652	10.238	-19.752
H	4.996	10.067	-18.606
C	4.736	8.420	-19.923
O	4.524	7.610	-19.045
O	4.901	8.053	-21.202
H	4.677	7.098	-21.254
C	0.011	0.563	-24.460
H	0.126	0.400	-25.530
H	-0.423	-0.342	-24.036
C	1.408	0.863	-23.918
O	2.103	1.784	-24.357
N	1.856	0.012	-22.970
H	1.231	-0.666	-22.539
H	2.811	0.103	-22.649
C	0.552	3.882	-20.160
H	0.451	3.979	-19.072
H	0.584	4.894	-20.572
C	1.853	3.199	-20.477
C	2.988	4.033	-21.092
C	2.109	1.921	-20.129
H	1.381	1.303	-19.607
C	4.128	3.358	-21.614
H	4.859	3.944	-22.158
C	3.392	1.314	-20.438
C	4.343	2.000	-21.416
O	2.899	5.272	-21.067
O	3.743	0.244	-19.914
O	5.283	1.311	-21.899
C	9.472	-1.116	-18.240
H	9.784	-1.509	-19.208
H	9.571	-0.030	-18.305
C	8.059	-1.470	-17.955
N	7.502	-2.706	-18.243
H	7.945	-3.444	-18.790
C	6.247	-2.736	-17.758
H	5.568	-3.563	-17.866
N	5.969	-1.580	-17.170
C	7.085	-0.778	-17.292
H	7.115	0.225	-16.899
C	6.022	3.744	-14.052
H	5.527	4.707	-14.224
H	5.752	3.425	-13.042
C	5.520	2.716	-15.019

N	5.009	3.001	-16.272
H	4.891	3.916	-16.704
C	4.574	1.856	-16.841
H	4.132	1.798	-17.823
N	4.778	0.838	-16.019
C	5.364	1.358	-14.881
H	5.634	0.728	-14.046
C	6.310	-2.792	-14.050
H	6.839	-2.482	-14.955
H	6.049	-1.876	-13.502
C	5.029	-3.508	-14.414
N	4.121	-2.931	-15.297
C	3.090	-3.763	-15.403
H	2.227	-3.613	-16.034
N	3.272	-4.844	-14.623
H	2.637	-5.653	-14.557
C	4.491	-4.702	-13.990
H	4.847	-5.446	-13.292
CU	4.240	-1.110	-16.337
O	4.411	-2.537	-20.046
H	5.039	-1.806	-20.256
H	4.973	-3.347	-20.198
O	3.304	3.155	-26.240
H	3.108	4.062	-25.944
H	2.923	2.593	-25.517
O	2.285	-0.603	-15.640
H	1.788	-0.795	-16.461
H	1.940	-1.246	-14.997
O	2.997	-1.492	-18.086
H	3.493	-2.114	-18.693
H	3.033	-0.692	-18.676
C	12.640	-0.422	-20.517
C	5.581	10.829	-20.552
C	-0.973	1.737	-24.264
C	-0.709	3.159	-20.707
C	10.436	-1.667	-17.162
C	7.555	3.959	-14.099
C	7.264	-3.606	-13.182

93

OXNA

C	11.992	-0.665	-21.882
H	12.215	-1.682	-22.208
H	12.447	0.019	-22.600
C	10.490	-0.531	-21.795
C	9.827	0.697	-21.676
H	10.390	1.626	-21.727
C	8.453	0.745	-21.458
H	7.943	1.696	-21.355
C	7.707	-0.441	-21.372
O	6.365	-0.417	-21.134
H	6.006	0.523	-21.308
C	8.347	-1.673	-21.519
H	7.761	-2.586	-21.469
C	9.725	-1.697	-21.720
H	10.257	-2.638	-21.789
C	4.689	9.917	-19.662
H	3.644	10.223	-19.775
H	4.976	10.047	-18.619
C	4.748	8.406	-19.946
O	4.492	7.595	-19.079
O	4.994	8.038	-21.209
H	4.956	7.055	-21.243
C	0.063	0.606	-24.418
H	0.293	0.477	-25.474
H	-0.349	-0.323	-24.028
H	0.968	0.849	-23.849
C	0.496	3.749	-19.960
H	0.359	3.587	-18.884
H	0.488	4.827	-20.138
C	1.847	3.225	-20.349
C	2.414	1.925	-19.778
C	2.654	4.048	-21.055
H	2.312	5.021	-21.396
C	3.687	1.521	-20.248
H	4.038	0.529	-19.979
C	4.087	3.787	-21.196
C	4.520	2.327	-21.042
O	1.769	1.265	-18.913
O	4.895	4.700	-21.314
O	5.602	1.973	-21.566
C	9.452	-1.128	-18.239
H	9.756	-1.528	-19.209
H	9.551	-0.042	-18.315
C	8.031	-1.474	-17.951
N	7.462	-2.707	-18.237
H	7.913	-3.466	-18.748
C	6.189	-2.709	-17.797
H	5.502	-3.529	-17.910
N	5.908	-1.539	-17.239
C	7.044	-0.756	-17.334
H	7.075	0.254	-16.959
C	6.024	3.733	-14.037
H	5.521	4.697	-14.183
H	5.767	3.391	-13.032
C	5.519	2.726	-15.022
N	5.069	3.043	-16.290
H	4.993	3.967	-16.709
C	4.623	1.923	-16.897
H	4.229	1.899	-17.904
N	4.762	0.888	-16.080
C	5.315	1.372	-14.909
H	5.525	0.723	-14.072
C	6.318	-2.766	-14.072
H	6.866	-2.462	-14.969
H	6.072	-1.845	-13.526
C	5.016	-3.443	-14.459
N	4.092	-2.835	-15.321
C	3.061	-3.667	-15.424
H	2.185	-3.503	-16.033
N	3.249	-4.771	-14.676
H	2.613	-5.579	-14.610
C	4.478	-4.644	-14.059
H	4.842	-5.403	-13.382
CU	4.137	-1.025	-16.517
O	4.623	-2.275	-20.270
H	5.284	-1.614	-20.599
H	5.098	-3.145	-20.305
O	2.835	3.576	-25.600
H	2.782	4.540	-25.776
H	3.086	3.530	-24.662
O	2.210	-0.282	-15.991
H	1.996	0.374	-16.690
H	1.505	-0.951	-16.071
O	3.067	-1.313	-18.340
H	3.634	-1.668	-19.083
H	2.600	-0.505	-18.673
C	12.645	-0.424	-20.515
C	5.580	10.827	-20.553
C	-0.975	1.741	-24.262
C	-0.721	3.141	-20.693
C	10.430	-1.667	-17.165
C	7.555	3.958	-14.097
C	7.256	-3.600	-13.189

60

TS(OXNA, OXrotNA)

C	11.990	-0.649	-21.890
H	12.238	-1.654	-22.235
H	12.432	0.057	-22.593
C	10.482	-0.568	-21.817
C	9.755	0.617	-21.644
H	10.268	1.576	-21.649
C	8.380	0.583	-21.422
H	7.819	1.501	-21.294
C	7.695	-0.644	-21.370
O	6.363	-0.712	-21.059
H	5.983	0.186	-21.298
C	8.395	-1.827	-21.609
H	7.856	-2.770	-21.601
C	9.770	-1.769	-21.824
H	10.352	-2.671	-21.959
C	4.691	9.934	-19.644
H	3.646	10.241	-19.757
H	4.985	10.068	-18.605
C	4.735	8.429	-19.921
O	4.511	7.615	-19.055
O	4.923	8.062	-21.204
H	4.788	7.093	-21.225
C	0.024	0.581	-24.489
H	0.059	0.344	-25.548

H	-0.299	-0.299	-23.941
H	1.023	0.877	-24.164
C	0.602	3.711	-20.253
H	0.661	3.651	-19.164
H	0.631	4.783	-20.485
C	1.825	3.084	-20.888
C	3.044	3.478	-20.156
C	2.055	2.294	-21.957
H	1.362	1.796	-22.614
C	4.217	2.723	-20.320
H	4.812	2.807	-19.443
C	3.390	2.097	-22.509
C	4.546	2.114	-21.527
O	2.930	4.391	-19.318
O	3.486	1.862	-23.705
O	5.607	1.545	-21.877
C	-0.849	7.742	-15.569
H	-0.657	8.150	-14.567
H	-1.004	8.593	-16.235
C	0.331	6.963	-16.026
C	1.078	6.198	-15.130
H	0.739	6.096	-14.101
C	2.255	5.590	-15.543
H	2.870	4.995	-14.874
C	2.690	5.771	-16.854
O	3.879	5.243	-17.204
H	3.808	5.048	-18.172
C	1.916	6.484	-17.780
H	2.255	6.581	-18.802
C	0.742	7.072	-17.356
H	0.141	7.651	-18.055
C	12.641	-0.424	-20.516
C	5.581	10.831	-20.548
C	-0.979	1.738	-24.269
C	-0.731	3.134	-20.726
C	-2.146	6.930	-15.540

93

OXrotNA

C	11.968	-0.653	-21.876
H	12.234	-1.647	-22.239
H	12.368	0.074	-22.582
C	10.462	-0.609	-21.754
C	9.709	0.567	-21.637
H	10.202	1.534	-21.713
C	8.339	0.517	-21.385
H	7.755	1.427	-21.300
C	7.683	-0.720	-21.251
O	6.365	-0.805	-20.936
H	5.894	0.071	-21.167
C	8.415	-1.901	-21.410
H	7.899	-2.853	-21.336
C	9.785	-1.827	-21.647
H	10.390	-2.724	-21.705
C	4.696	9.931	-19.644
H	3.651	10.239	-19.752
H	4.995	10.069	-18.605
C	4.735	8.420	-19.919
O	4.525	7.613	-19.038
O	4.894	8.048	-21.197
H	4.663	7.095	-21.244
C	0.064	0.607	-24.416
H	0.298	0.480	-25.472
H	-0.348	-0.324	-24.030
H	0.967	0.851	-23.845
C	0.562	3.864	-20.162
H	0.471	3.947	-19.072
H	0.598	4.881	-20.560
C	1.861	3.180	-20.495
C	3.000	4.039	-21.079
C	2.116	1.890	-20.192
H	1.382	1.250	-19.707
C	4.168	3.389	-21.561
H	4.915	3.998	-22.058
C	3.436	1.321	-20.429
C	4.426	2.045	-21.338
O	2.878	5.278	-21.073
O	3.802	0.285	-19.851
O	5.440	1.406	-21.735
C	9.473	-1.117	-18.242
H	9.784	-1.512	-19.209
H	9.573	-0.031	-18.309
C	8.059	-1.467	-17.955
N	7.497	-2.700	-18.247
H	7.937	-3.436	-18.800
C	6.245	-2.731	-17.757
H	5.562	-3.553	-17.869
N	5.974	-1.578	-17.158
C	7.091	-0.776	-17.282
H	7.126	0.224	-16.883
C	6.021	3.742	-14.053
H	5.526	4.704	-14.226
H	5.751	3.423	-13.043
C	5.521	2.712	-15.020
N	5.018	2.996	-16.277
H	4.903	3.909	-16.711
C	4.588	1.851	-16.848
H	4.157	1.790	-17.835
N	4.786	0.833	-16.022
C	5.364	1.355	-14.881
H	5.628	0.727	-14.043
C	6.310	-2.791	-14.050
H	6.842	-2.479	-14.953
H	6.048	-1.877	-13.501
C	5.030	-3.507	-14.419
N	4.126	-2.932	-15.307
C	3.094	-3.763	-15.415
H	2.234	-3.612	-16.051
N	3.272	-4.842	-14.632
H	2.636	-5.650	-14.566
C	4.488	-4.700	-13.995
H	4.841	-5.443	-13.294
CU	4.246	-1.110	-16.342
O	4.421	-2.534	-20.039
H	5.044	-1.804	-20.261
H	4.978	-3.346	-20.200
O	2.975	3.478	-25.793
H	2.871	4.451	-25.851
H	3.070	3.297	-24.844
O	2.303	-0.608	-15.648
H	1.804	-0.777	-16.473
H	1.947	-1.257	-15.017
O	3.015	-1.488	-18.086
H	3.499	-2.113	-18.701
H	3.056	-0.685	-18.673
C	12.642	-0.423	-20.516
C	5.581	10.829	-20.552
C	-0.975	1.742	-24.262
C	-0.708	3.157	-20.707
C	10.436	-1.667	-17.162
C	7.555	3.958	-14.099
C	7.264	-3.606	-13.182
